# Amphiphilic Polyphenylene Dendron Conjugates for Surface Remodeling of Adenovirus 5

**DOI:** 10.1002/anie.201913708

**Published:** 2020-02-03

**Authors:** Jessica Wagner, Longjie Li, Johanna Simon, Lea Krutzke, Katharina Landfester, Volker Mailänder, Klaus Müllen, David Y. W. Ng, Yuzhou Wu, Tanja Weil

**Affiliations:** ^1^ Max Planck Institute for Polymer Research Ackermannweg 10 55128 Mainz Germany; ^2^ Graduate School Materials Science in Mainz Staudingerweg 9 55128 Mainz Germany; ^3^ Hubei Key Laboratory of Bioinorganic Chemistry and Materia Medica School of Chemistry and Chemical Engineering Huazhong University of Science and Technology 1037 Luoyu Road 430074 Wuhan China; ^4^ Department of Dermatology University Medical Center of the Johannes Gutenberg-University Mainz Langenbeckstr. 1 55131 Mainz Germany; ^5^ University Ulinic Department of Gene Therapy Helmholtzstr. 8/1 89081 Ulm Germany

**Keywords:** amphiphiles, dendrimers, gene technology, proteins, viruses

## Abstract

Amphiphilic surface groups play an important role in many biological processes. The synthesis of amphiphilic polyphenylene dendrimer branches (dendrons), providing alternating hydrophilic and lipophilic surface groups and one reactive ethynyl group at the core is reported. The amphiphilic surface groups serve as biorecognition units that bind to the surface of adenovirus 5 (Ad5), which is a common vector in gene therapy. The Ad5/dendron complexes showed high gene transduction efficiencies in coxsackie‐adenovirus receptor (CAR)‐negative cells. Moreover, the dendrons offer incorporation of new functions at the dendron core by in situ post‐modifications, even when bound to the Ad5 surface. Surfaces coated with these dendrons were analyzed for their blood‐protein binding capacity, which is essential to predict their performance in the blood stream. A new platform for introducing bioactive groups to the Ad5 surface without chemically modifying the virus particles is provided.

## Graphical abstract

**Amphiphilic polyphenylene dendrons** were prepared and bound to gene vector adenovirus 5 (Ad5) through polar and nonpolar surface groups that control its cellular uptake. The new dendron layer at the Ad5 surface provides reactive groups that are accessible for post‐modifications at the virus surface.

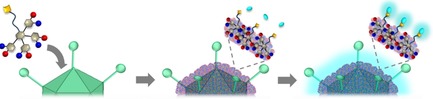

## Introduction

Amphiphilicity plays an important role in the formation of biological architectures such as the structure of proteins, the self‐assembly of peptides, or the build‐up of biological membranes.^1^ Because of the characteristics of amphiphiles to organize into higher ordered structures,^2^ their interactions with other biomolecules is a complex process of high biological relevance, which is still not fully understood. For example, the exposure of nanomaterials like polymers, liposomes, or nanoparticles to biological fluids, such as human blood plasma, gives rise to a protein corona around nanoparticles that also directs their transport in vivo.^3^ It has been demonstrated that either the variation of surface charges^4^ or coating of nanoparticles, for example, with polymers like polyethylene glycol,^5^ has an impact on the protein corona and often controls their aggregation^6^ and biodistribution,^7^ as well as cellular uptake properties.^5^ By employing amphiphilic surface patterns on nanoparticles, their influence on biological systems was studied.^8^ It is still very challenging to control the surface contour of nanoparticles8b and to impart distinct amphiphilic surface patterns with molecular precision that maintains their perfect nanosize definition in various biological environments.^9^ Therefore, highly branched macromolecules with precise structures and molecular weights, such as dendrimers, have emerged as a monodisperse platform providing characteristic features of proteins.^10^ Hence, they are often referred to as “artificial proteins”^11^ and their applications in biomedicine range from drug delivery of serum albumin mimicking polyphenylene dendrimers^12^ to multivalent dendrimers as antiviral drugs^13^ and gene delivery agents.^14^ For example, it has been demonstrated that dendrons bind to a virus capsid by supramolecular interactions, leading to an electrostatically driven self‐assembly into dendron‐virus complexes. These complexes could be disassembled by an optical trigger to release the virus.^15^

Amphiphilic polyphenylene dendrimers (PPDs) are macromolecules with given surface patterns consisting of, for example, alternating sulfonic acid and *n*‐propyl groups.^16^ These dendrimers are internalized into cells while showing low toxicity both in vitro and in vivo and they possess the ability to transport lipophilic drugs within their nonpolar inner cavities.^12^ PPDs are unique because of the rigidity of their sterically demanding and space‐filling pentaphenyl‐benzene scaffold, and therefore provide persistent three‐dimensional structures.^17^ This class of dendrimer has the advantage that surface patterns can be exactly positioned since no backfolding of single dendritic arms (dendrons) can occur.^18^ Furthermore, we have shown previously that out of a set of amphiphilic PPDs, only one type of PPD, with high density of amphiphilic surface patterns, was able to bind to adenovirus 5 (Ad5).^19^ Less‐branched amphiphilic PPDs showed a significantly lower binding to Ad5 and a negatively charged PPD surface did not lead to any binding. These findings indicated that the dense amphiphilic surface motif is required for Ad5 binding.^19^

Adenovirus (Ad) is a non‐enveloped double‐stranded DNA virus with an icosahedral capsid infecting respiratory epithelial cells.^20^ Ads are the most common vectors in gene therapy because of their significant advantages, such as genetic stability, well‐characterized biology, and high transduction efficiency in cells.20a, 21 They enter cells by specific interaction with the coxsackie‐adenovirus receptor (CAR) and integrins, limiting applications to such cell types.^22^ Moreover, the three major capsid proteins—hexon protein, penton base, and fiber—bind to antibodies, which lead to immunogenic responses or neutralization, which needs to be reduced for in vivo applications.20a One strategy focuses on shielding the Ad surface from antibody binding by covalent attachment of polymers like polyethylene glycol.^23^

**Figure 1:**
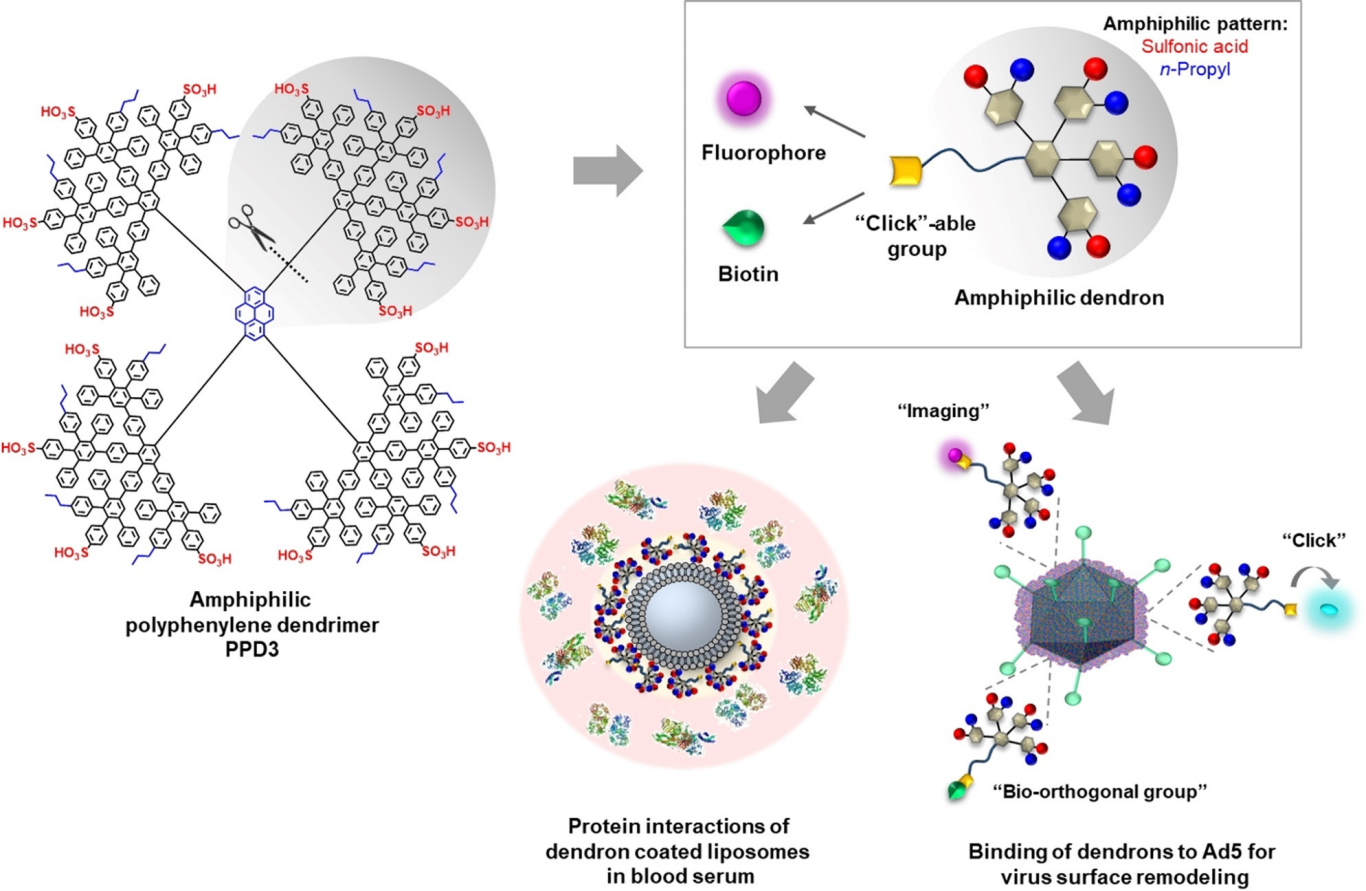
Structural design of an amphiphilic polyphenylene dendron by desymmetrization of amphiphilic dendrimer PPD3. By the employment of a clickable ethynyl group, the introduction of a second function by post‐modification was achieved. The amphiphilic pattern of these dendron‐conjugates interacts with biological structures like proteins and was verified by interactions of dendron‐coated liposomes with blood serum proteins (protein structures: PDB‐files 4NHH,^24^ 1FZC,^25^ 5Z0B^26^) as well as binding to Ad5 for re‐direction into CAR‐negative cells.

We discovered recently that the formation of an amphiphilic PPD corona promotes cellular internalization into CAR‐negative cells, which cannot be intrinsically targeted by Ad5.^19^ In human blood serum, neutralization by antibodies and binding of coagulation factor X (FX), the primary transport mechanism of Ad5 to the liver, were altered significantly after PPD adsorption. We show that there are no electrostatic interactions between the positively charged fibers of Ad5 and negatively charged sulfonic acids of the PPD. In addition, FX could not bind to Ad5 when shielded with the dendrimer, indicating that the PPD blocks the binding site for FX. As the amphiphilic PPDs bind to the virus capsid proteins, they also impart a novel surface pattern onto Ad5, controlling their various interactions with other blood serum proteins.^19^ As a consequence, reduced gene transduction in liver tissue and an enhanced transduction in heart tissue were observed in vivo.^19^ Thus, amphiphilic PPDs provide a novel platform for virus redirection into different cells and tissues because of their ability to coat and protect Ad5 from FX binding, which influences cellular uptake and biodistribution of Ad5 in vivo. Enhanced structural variability of the dendrimer scaffold is a prerequisite to further advance the applicability of amphiphilic PPDs in biomedicine. Therefore, a dendritic structure that enables the incorporation of additional functionalities is required (Figure 1).

Consequently, we combine the defined amphiphilic pattern of PPD3 for biorecognition with a novel synthetic handle to enable post‐modifications (Figure 1). Since PPDs are symmetric macromolecules, the incorporation of an additional feature is challenging while retaining the desired amphiphilic surface structures. Therefore, we desymmetrized the PPD3 structure and synthesized the first amphiphilic polyphenylene dendron having one dendrimer branch, that is, one quarter of the entire PPD3, which combines Ad5 binding features with the potential for post‐modification. We demonstrate a novel multistep synthesis of an amphiphilic polyphenylene dendron with a propargyl‐modified triethylene glycol linker at the core. By applying this linker, high water solubility as well as the possibility to introduce functional units like either a fluorophore for imaging or a bio‐orthogonal group by copper(I)‐catalyzed azide–alkyne cycloaddition (CuAAC) are envisioned. Additionally, upon complexation with Ad5, the alkyne group can act as a functional handle for in situ CuAAC to serve as a versatile platform for introducing chemical modifications on the viral surface. Furthermore, we analyzed the binding of blood proteins to understand the influence of amphiphilic dendron‐coated surfaces in the blood stream.

## Results and Discussion

**Scheme 1:**
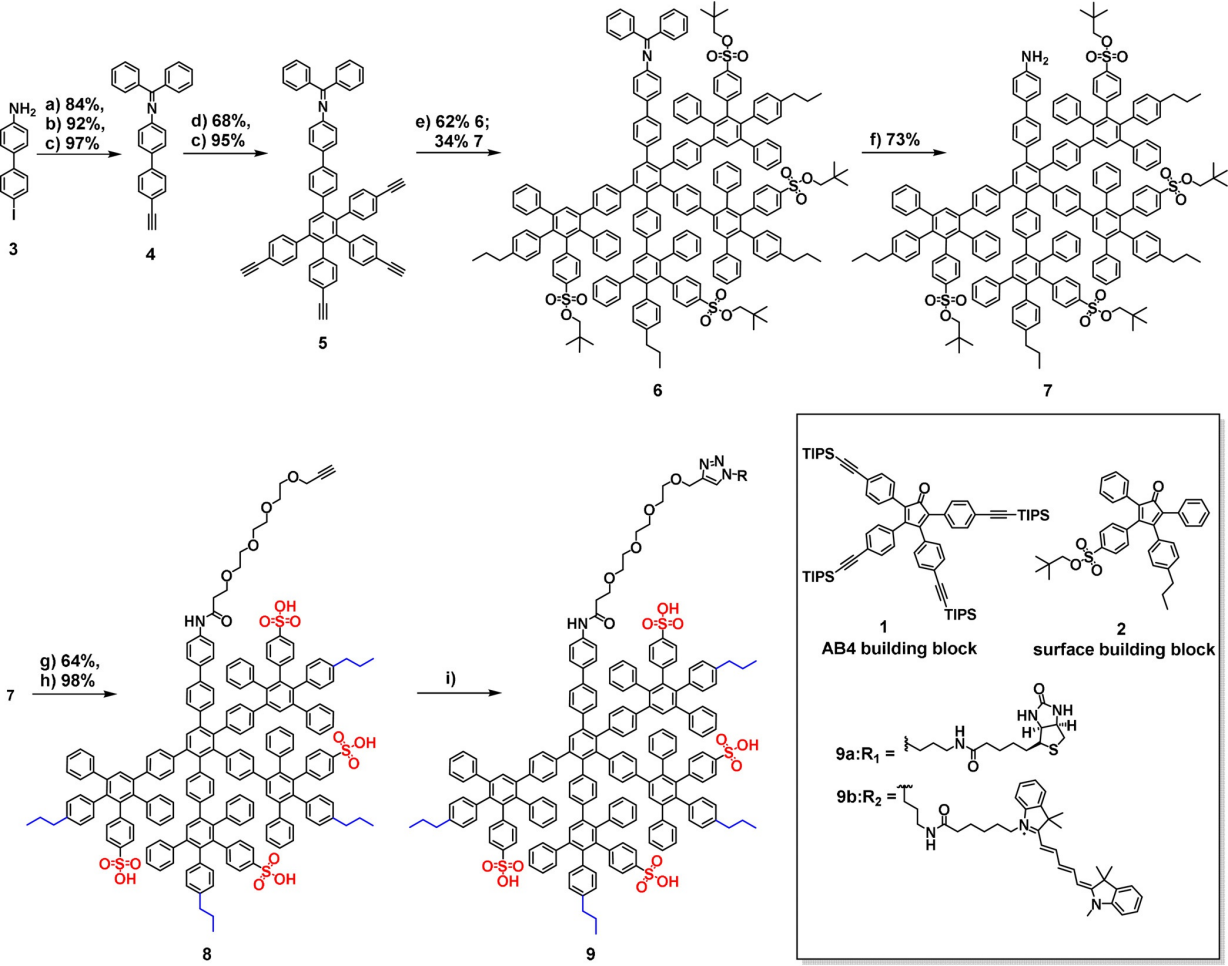
Synthesis of amphiphilic polyphenylene dendron conjugates. a) Benzophenone, toluene, and molecular sieves 4 Å, 15 h; b) TIPS‐acetylene, CuI, Pd(Ph_3_P)_2_Cl_2_, THF/NEt_3_ (5:1), RT, 15 h; c) TBAF, THF, 0 °C, 0.5 h; d) AB4 building block, *o*‐xylene, 160 °C, 24 h; c) TBAF, THF, 0 °C, 0.5 h; e) Surface building block, *o*‐xylene, 145 °C, 48 h; f) HCl, THF, 20 min; g) propargyl‐TEG‐COOH, EDC⋅HCl, DMAP, DMF, RT, 24 h; h) DMF, 180 °C, 36 h; i) RN_3_, CuSO_4_, sodium ascorbate, TBTA, DMF/H_2_O (7:2 for **9a**; 7:1 for **9b**), RT, 24 h.

The divergent synthesis of a second‐generation amphiphilic dendron (Scheme 1) is based on iterative [4+2] Diels–Alder cycloadditions of an ethynylated core with tetraphenylcyclopentadienones (CPs). The CP either determines the branching or serves as an end‐capping unit introducing the surface patterns.^27^ As previously reported, a high density of amphiphilic surface groups resulting from a highly branched dendrimer scaffold leads to Ad5 binding.^19^ To integrate this dense surface pattern into a dendron scaffold, we synthesized a dendritic branch with similar amphiphilic surface groups, representing one quarter of the entire dendrimer. Therefore, the AB4 building block **1**, with four branching points was used, and was synthesized based on modified protocols from Morgenroth et al.^28^ The synthesis scheme of **1**, as well as all reaction conditions are summarized in Figure S1A (see the Supporting Information). The bifunctionalized CP **2**, with an *n*‐propyl group and neopentyl‐protected sulfonic acid group, was applied as an end‐capping unit and was synthesized based on modified protocols from Stangenberg et al.^16^ The syntheses and reaction conditions are summarized in Figure S1B.

In this study, the neopentyl‐protected amphiphilic polyphenylene dendron **7** was synthesized and post‐modified with a propargyl‐TEG‐linker followed by attachment of either a d‐biotin or Cyanine 5 (Cy5) moiety by a CuAAC (Scheme 1). Cy5 was introduced for cellular uptake experiments and co‐visualization of dendron uptake and Ad5 gene delivery. d‐Biotin was attached as an example of a bio‐orthogonal group to study the influence of a functional group on Ad5 binding.

The detailed structure of the dendron core is crucial since its accessibility, as well as peripheral functionality after dendron growth are required. The dendron **7** was synthesized from a bifunctional biphenyl dendron core (**3**), which allowed minimization of the steric hindrance for the post‐modification step. The iodo group of **3** enabled the coupling with an ethynyl group that is required for dendron growth, while the aniline group offered the possibility for post‐modification at the focal point after dendron synthesis. In a condensation reaction, the aniline group was protected with benzophenone, resulting in an imine to prevent side reactions of the amine group during the harsh reaction conditions of PPD synthesis. In the next step, the triisopropylsilyl (TIPS) protected ethynyl group was introduced by Sonogashira–Hagihara coupling followed by removal of the silyl groups to afford the dendron core **4** in good yields (75 % over three steps). Further dendron growth was conducted by utilizing the branching unit **1** (AB4^28^) in a [4+2] Diels–Alder cycloaddition under standard reaction conditions. Since **4** only features one dienophile (ethynyl group), the reaction time was reduced from 48^12^ to 24 hours. After deprotection of the ethynyl groups, the first generation dendron **5** was obtained in 65 % yield over two steps. Subsequently, the next [4+2] Diels–Alder reaction with **2** was performed for 48 hours at reduced temperatures of 145 °C to avoid deprotection of the sulfonic acid groups.^16^ Under these reaction conditions, the imine protective group was partially cleaved so that imine‐dendron **6** (62 %) as well as amine‐dendron **7** (34 %) were isolated by column chromatography. The remaining imine protective group of **6** was removed by acidic treatment to afford **7**. After ligation of **7** to a triethylene glycol (TEG) derivative (propargyl‐TEG‐linker) by amide coupling, the sulfonic acid groups were deprotected thermally to obtain the propargyl‐TEG‐dendron **8**. The removal of the neopentyl group requires high temperatures so that the heat‐sensitive bioactive groups were attached after deprotecting the sulfonic acid groups. d‐Biotin and Cy5 derivatives were ligated by ligand‐accelerated CuAAC, applying tris((1‐benzyl‐4‐triazolyl)methyl)amine (TBTA) as the ligand. The crude products were purified by gel permeation chromatography (GPC) in *N*,*N*′‐dimethylformamide to remove CuAAC reagents and unreacted starting materials to afford the biotinylated dendron **9 a** and Cy5‐dendron **9 b**. A detailed reaction scheme is provided in Figure S2.

Because of the asymmetry of **2**, second‐generation dendrons were obtained as constitutional isomers, as reported previously.^16^, ^29^ The constitutional isomers were confirmed by ^1^H NMR spectroscopy, where they are most notably visible in the spectra of the neopentyl‐protected dendrons (see Figure S5). NMR spectroscopy and MALDI‐TOF spectrometry demonstrate the successful synthesis of the propargyl‐modified dendron **8** (see Figures S6, S7, and S13) and post‐modification by CuAAC to achieve **9 a** and **9 b** (see Figures S8–S12, S14, and S15). The signals in the ^1^H NMR spectrum can be assigned to **8**, **9 a**, and **9 b**. The detailed synthesis description, as well as characterization of dendron intermediates and final products are summarized in the Supporting Information.

**Figure 2:**
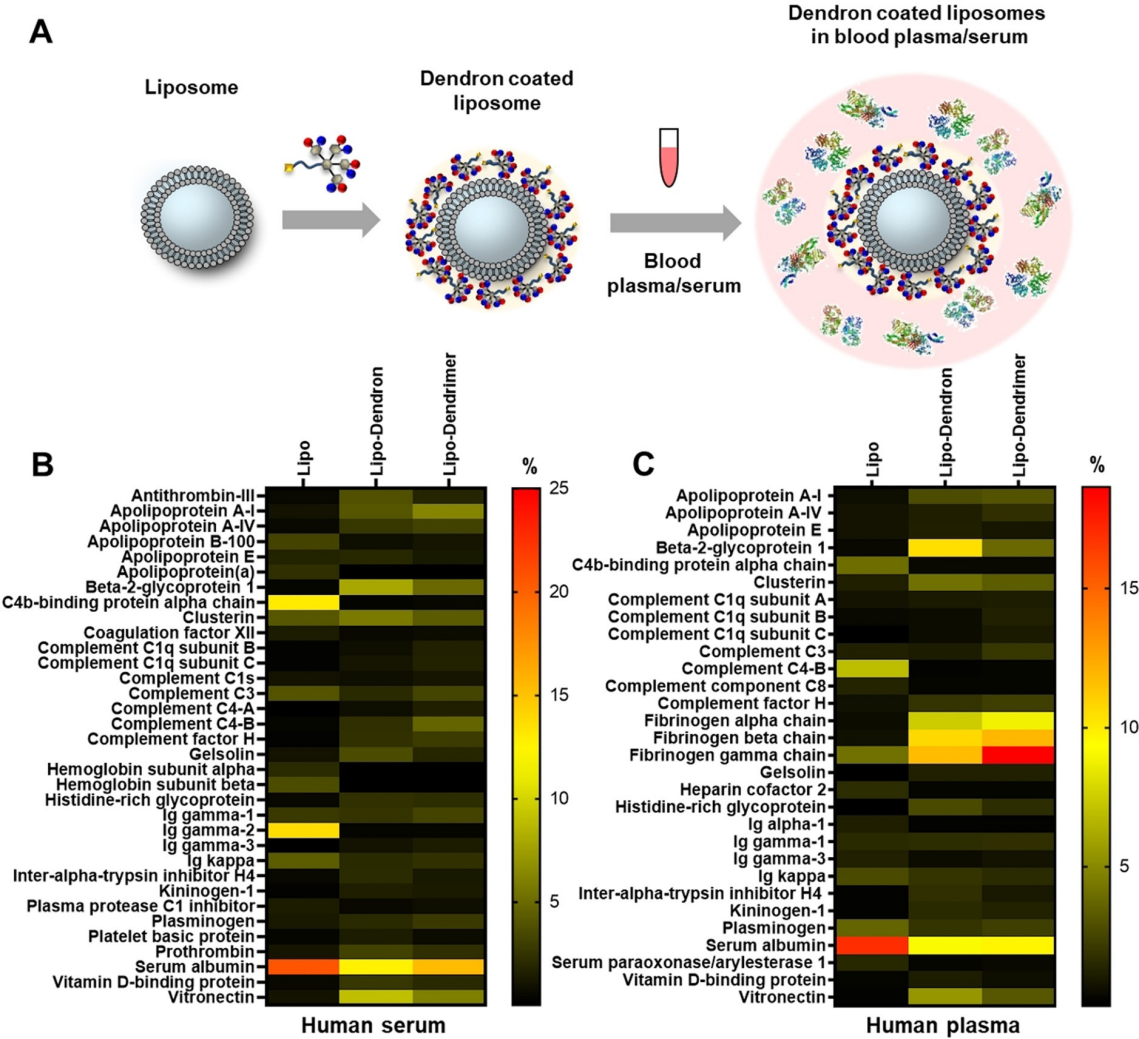
Dendron‐coated liposomes form a protein corona in blood serum/plasma. A) Coating of liposomes with **8** and incubation with blood serum or blood plasma leading to protein corona (protein structures: PDB‐files 4NHH,^24^ 1FZC,^25^ 5Z0B^26^). Heat map of adsorbed proteins to **8** and dendrimer PPD3‐coated liposomes in blood serum (B) and blood plasma (C). The amount of each protein is given in % based on all identified corona proteins. A list of all identified proteins is provided in Figures S34–S37.

It is well known that Ads are involved in several protein interactions in the blood stream.^30^ However, after PPD3 complexation, blood coagulation factor X (FX) could not bind to Ad5.^19^ To shed light on the potential interaction partners of amphiphilic dendrons in the blood stream, we analyzed proteins binding to surfaces coated with **8**. The virus capsid was simplified by applying nanocarriers as already validated model systems with less complexity.^31^ Thus, liposomes31a prepared from 1,2‐dioleoyl‐*sn*‐glycero‐3‐phosphoethanolamine (DOPE), l‐α‐phosphatidylcholine (egg PC) and cholesterol (Chol) (PC:DOPE:Chol=1:1:1, Ø=242±6 nm), as well as polystyrene particles (PS‐NH_2_, Ø=98±10 nm)31b with comparable sizes to Ad5 were coated with the amphiphilic dendrimer PPD3 and **8** (for details see the Supporting Information). Then, the protein adsorption in blood plasma as well as in blood serum was analyzed. As mentioned above, changes of surface charges as well as polymer coatings can influence the protein corona of nanoparticles.4b As previously reported, amphiphilic patterned PPDs seem to bind to a lipid monolayer by electrostatic interactions between negatively charged sulfonic acid groups and positively charged headgroups of the lipid (see Figure S22).^32^ Here, the dendron/dendrimer‐coated liposomes and polystyrene (PS) particles were prepared following a standard protocol.^5^, 31b Briefly, either liposomes or nanoparticles were incubated with excess dendron or PPD3 and purified by centrifugation. The coating was verified by DLS and zeta potential (see Table S1). Then, either blood serum or plasma was added (Figure 2 A). After centrifugation, washing steps, and desorption of corona proteins, the isolated proteins were analyzed qualitatively by SDS‐PAGE (see Figures S17–S20) and quantitatively by a Pierce Assay (see Figure S21) and LC‐MS/MS (Figure 2 B,C; see Figure S16).

Coating with either **8** or PPD3 had a major impact on protein corona formation of liposomes (Figure 2) and PS nanoparticles (see Figure S16). For unfunctionalized liposomes and PS nanoparticles, we detected high amounts of albumin (ca. 26±3 %) and immunoglobulins (e.g. Ig kappa, ca. 6±1 %) in the protein corona after serum incubation (Figure 2 B). However, after coverage with **8** (lipo‐dendron) the amount of Ig kappa is significantly lower (ca. 2±0.2 %). Immunoglobulins belong to the protein class of opsonins and can mediate the interaction with phagocytic cells.^33^ The adsorption of IgG can dramatically reduce the blood circulation time, and thereby also reduce the interaction with targeted cells.^34^ As reported in the literature, the protein source additionally shapes the protein corona formation.^35^ This observation is in line with our findings as we have observed differences in the protein corona composition after serum and plasma incubation (Figure 2 B versus C).

For serum preparation, blood was clotted and then centrifuged to remove the clot. The resulting supernatant no longer contained all proteins. Because of this preparation, fibrinogen and other clotting factors were removed. In contrast, for plasma preparation, an anticoagulant was added to prevent blood clotting and therefore the plasma contained all blood proteins including the clotting factors. For **8**‐ and PPD3‐covered liposomes, the amount of fibrinogen was higher than for the unfunctionalized ones after plasma incubation. Comparable results were obtained for **8**‐ and PPD3‐coated PS (see Figures S16–S18), indicating that the protein interactions are governed by the **8** and PPD3 coating and not by the underlying base material. In addition, minor differences were observed for the protein corona composition of lipo‐dendron and lipo‐dendrimer after serum and plasma incubation. This effect was most prominent for β‐2 glycoprotein 1, also known as Apolipoprotein H (ApoH).4b The corona of lipo‐dendron was enriched with ApoH (8±0.5 % serum, 11±1.5 % plasma), whereas lower amounts were detected for both lipo‐dendrimer (5±0.3 % serum, 4±0.3 % plasma) and unfunctionalized liposomes (0.5±0.2 % serum, 0.3±0.5 % plasma). It has been previously shown that the coating of PS nanoparticles with ApoH leads to a favorable interaction with human mesenchymal stem cells.4b A similar trend was observed for the adsorption of vitronectin, which increased in case of the lipo‐dendron compared to either PPD3‐coated or unfunctionalized liposomes. Here, we found that the protein corona of the lipo‐dendron was enriched with vitronectin (9±0.8 % serum, 6±0.5 % plasma), whereas lower amounts were adsorbed on either the lipo‐dendrimer (6±0.5 % serum, 3±0.2 % plasma) or unfunctionalized liposomes (1±0.7 % serum, 0.2±0.3 % plasma). Interestingly, vitronectin was also detected in the protein corona of DOTAP/DNA lipoplexes and it was demonstrated that vitronectin could mediate a selective uptake of the lipoplexes towards MDA‐MB‐435S cancer cells, which have a high expression level of the vitronectin α_ν_β_3_ integrin receptor.^36^ Taken together, these results indicate that coating with **8** favors the interactions with specific blood proteins, which eventually also determine the interactions with cells and cellular uptake behavior.

**Figure 3:**
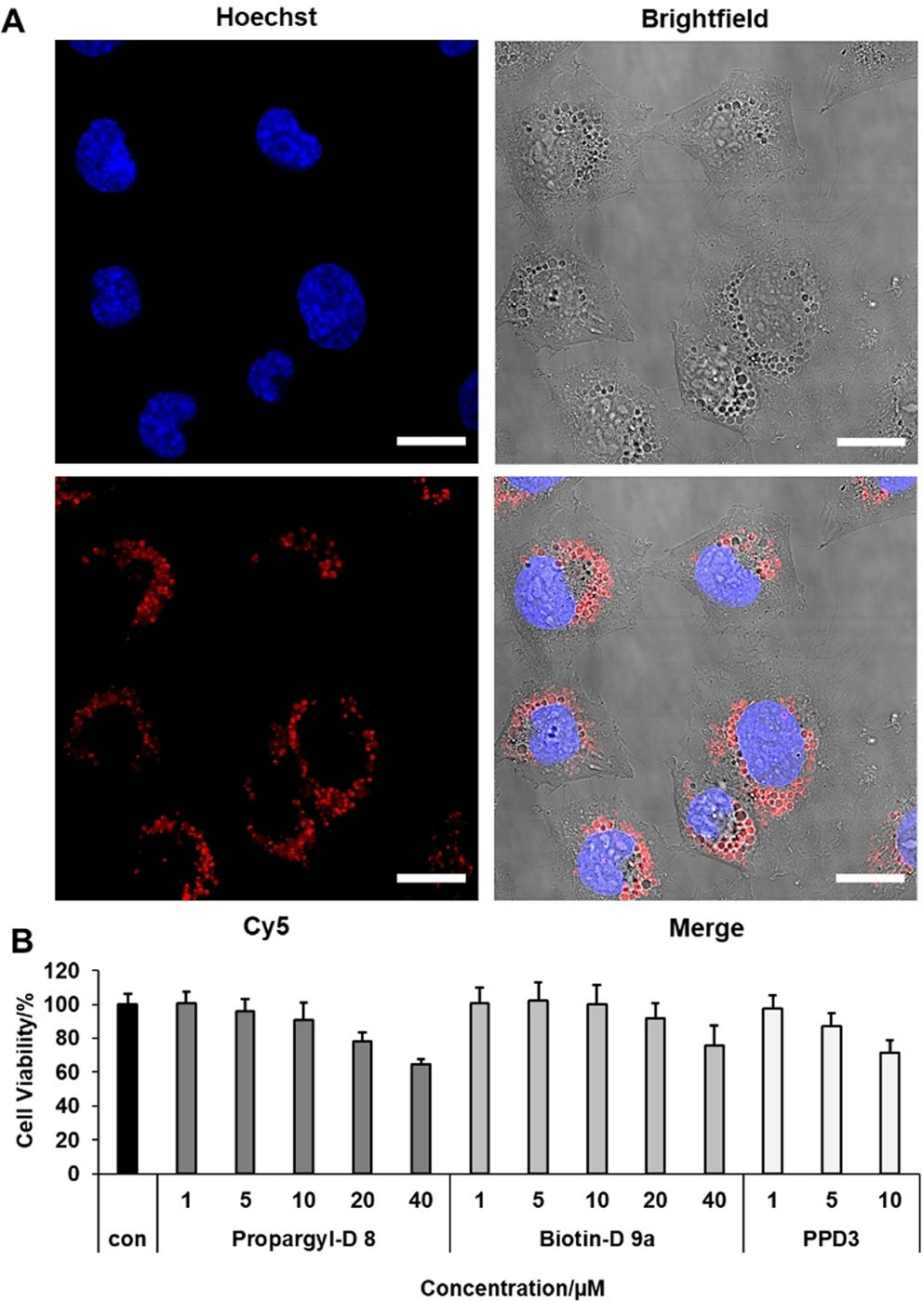
Cellular internalization of amphiphilic dendrons and cell viability on CHO‐K1. A) Confocal image of CHO‐K1 cells incubated with 1 μm Cy5‐Dendron **9 b** for 24 h and nucleus staining with Hoechst 33258 (scale=20 μm). B) Cell viability of **8** and **9 a** compared to PPD3 by applying four times higher dendron concentration to achieve approximate same quantities of surface patterns. Cell viability was tested with CellTiter‐Glo®‐Assay. Data from three independent experiments with quadruplicates (total *n*=12) is shown.

Since amphiphilic dendrimers with alternating sulfonic acid and *n*‐propyl groups are internalized into cells and transported by vesicles,^12^ we tested the cellular uptake of the dendron to assess whether the surface pattern of a desymmetrized dendron is similar to the symmetric PPD3 dendrimer. The Chinese Hamster ovary cell line CHO‐K1 was selected, since it possesses low CAR‐expression, rendering it also suitable for gene transduction experiments within this study. The cells were incubated with 1 μm **9 b** for 24 hours, washed with phosphate buffered saline (PBS), and the nucleus was stained with Hoechst 33258. The cellular internalization was followed by confocal laser scanning microscopy. As depicted in Figure 3 A, **9 b** was internalized into CHO‐K1 cells. We observed that **9 b** was located in vesicles in a similar way reported for amphiphilic PPDs.^12^ The comparison with the blank control is provided in Figure S23.

Cell compatibility of **8** and **9 a** was compared to PPD3 in CHO‐K1 cells by a cell viability assay. Cells were treated with 1–40 μm **8** and **9 a** as well as 1–10 μm PPD3 for 24 hours. We used four equivalents of dendron conjugates compared to PPD3 to adjust the numbers of surface patterns to approximately similar quantities. The cell viability was determined by quantification of the ATP levels by applying the CellTiter‐Glo®‐Assay. Both dendron conjugates and PPD3 displayed no significant cytotoxicity up to 20 and 5 μm, respectively.

**Figure 4:**
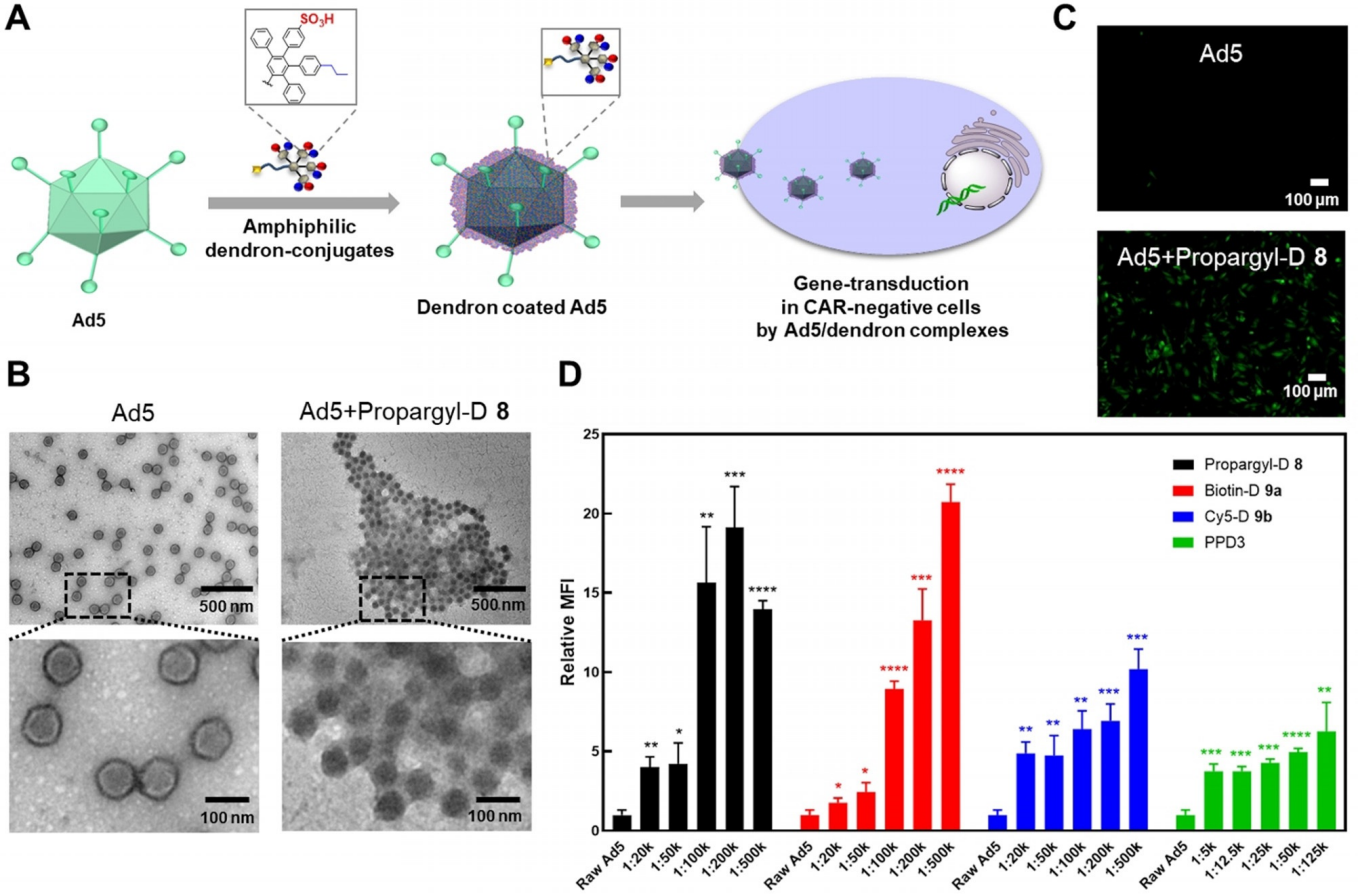
Dendron‐Ad5 complex formation leads to EGFP‐transduction in CAR‐negative cells. A) Coating of Ad5 by dendrons and the potential for cellular uptake into CAR‐negative cells resulting in gene transduction. B) TEM images show binding of dendrons to Ad5 (Ad5: Dendron=1: 100k). C) Fluorescent microscopy image of EGFP‐transduction in CAR‐negative CHO‐K1 cells (Ad5: Dendron=1: 500k). D) Flow cytometry of CHO‐K1 cells incubated with Ad5‐dendron complexes with ratios of Ad5: Dendron=1:20k‐1:500k and Ad5: PPD3=1:5k‐1:125k, respectively (*n*=3, * represents *p*‐value <0.05, ** represents *p*‐value <0.01, *** represents *p*‐value <0.001, **** represents *p*‐value <0.0001).

Next, we studied the performance of the dendron conjugates in Ad5‐assisted gene transduction (Figure 4 A). The formation of a dendron corona was studied by transmission electron microscopy (TEM), dynamic light scattering (DLS), and zeta potential, and we performed a functional assay to assess its capability to transport Ad5 into CAR‐negative cells as monitored by fluorescence microscopy. The influence of surface patterns on gene transduction was compared with PPD3 using flow cytometry. Consequently, **8**, **9 a**, and **9 b**, as well as PPD3, were mixed with Ad5 at the ratios of 1:20k to 1:500k (Ad5/dendron) and 1:5k to 1:125k (Ad5/PPD3), respectively, in PBS and complex formation as well as gene transduction of Ad5 in low CAR expressing cell line CHO‐K1 were tested. For all experiments, four equivalents of dendron related to PPD3 were used to compare their properties while maintaining approximately the same surface pattern. Here we used an eGFP‐expressing Ad5 and analyzed its interaction with the dendrons. TEM analysis of vector particles incubated with the dendron for 40 minutes at a molar ratio of 1:100k was performed. Results clearly confirmed that **8**, **9 a**, and **9 b** bound to and formed complexes with Ad5 (Figure 4 B; see Figure S24). To further analyze this interaction, DLS measurements were conducted. Our data shows that dendrons alone self‐assemble in solution because of their intrinsic amphiphilicity (see Table S5 and Figure S26B). Nevertheless, in the presence of Ad5, all dendrons demonstrably bound to the vector particles, which was clearly indicated by an increase of the vector particle size (see Tables S3 and S4, and Figures S25 and S26A). To further confirm this interaction, we measured the surface charge of dendrons alone, Ad5 alone, and dendron‐coated Ad5 by zeta‐potential analysis. Dendron‐coated vector particles showed a ratio‐dependent increase of the negative surface charge compared to dendrons alone or Ad5 particles alone (see Tables S3–S5), confirming the binding of Ad5 and analyzed dendrons. Additionally, we could show that at ratios beyond 1:1000k for Ad5/**8** and 1:200k for Ad5/**9 a**, saturation of Ad5 particles is reached, which results in free, unbound dendron molecules in solution (see Table S4 and Figure S26).

**Figure 5:**
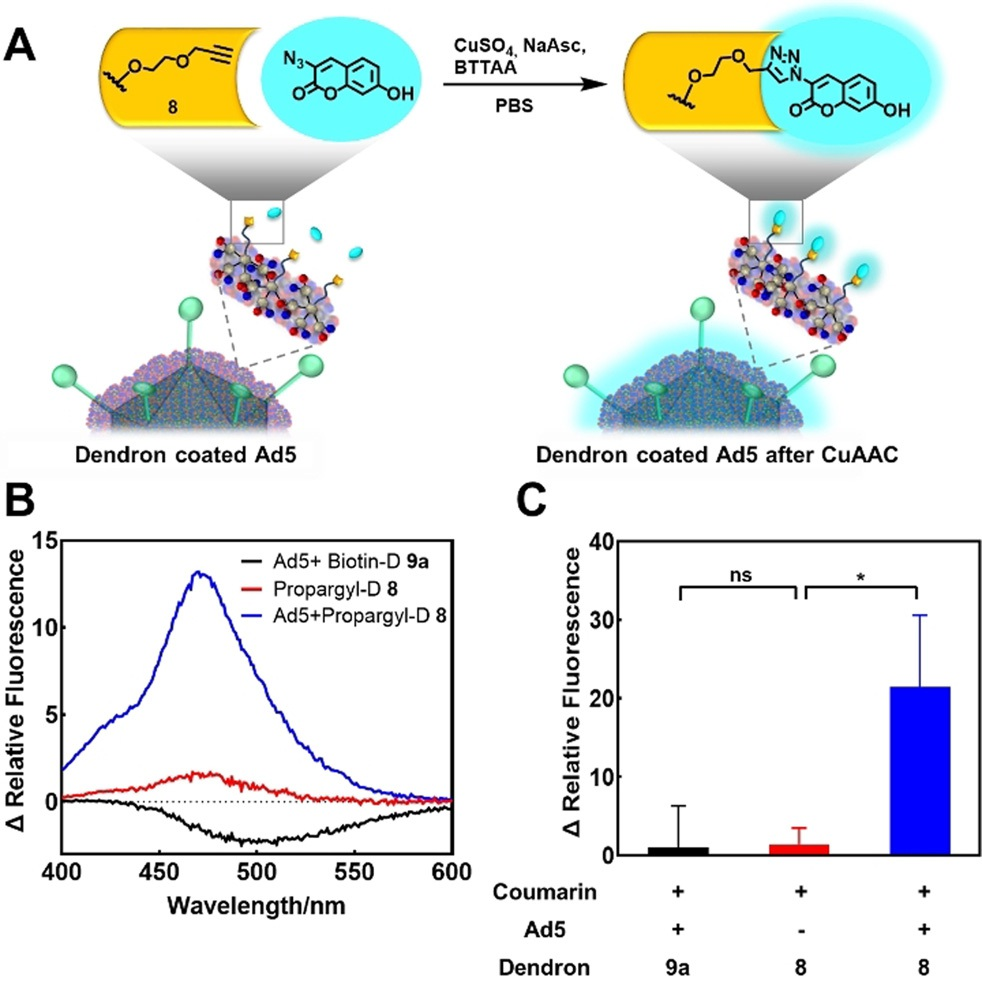
CuAAC on dendrons bound to Ad5. A) CuAAC of **8** with 3‐azido‐7‐hydroxycoumarin on the Ad5 surface leads to fluorescence of the dendron‐fluorophore conjugate (*λ*_abs_=404 nm, *λ*_em_=477 nm after click reaction). B) Fluorescence spectra of dendron conjugates after treatment with CuAAC reagents. Ad5 was mixed with either **8** or **9 a** (negative control). After incubation for 40 min, unbound dendron was removed by ultrafiltration. The dendron **8** alone was treated under the same reaction conditions. Then, CuAAC reagents were added and fluorescence spectra were recorded after incubation for 1 h. 3‐azido‐7‐hydroxycoumarin was subtracted as background. C) The change in relative fluorescence of Ad5+Biotin‐D **9 a** group, Propargyl‐D **8** group (with ultrafiltration), and Ad5+Propargyl‐D **8** group at 477 nm (emission) after treatment are described in (B). 3‐Azido‐7‐hydroxycoumarin was subtracted as background and the change in relative fluorescence of Ad5/propargyl‐dendron after CuAAC (blue column) is relative to the Ad5+Biotin‐D **9 a** group (black column) that serves as negative control (*n*=3, * represents *p*<0.05, ns means not significant).

We previously used biolayer interferometry (BLI) to study the binding strength of an amphiphilic dendrimer binding motif to Ad5.^19^ This method required the immobilization of the dendrimer on a streptavidin‐coated surface (see Figure S32). Thus, we used **9 a** and an equilibrium dissociation constant value of *K*_D_=1.27 pm was determined (see Figure S33 and Table S6). Since the dendron binds to Ad5 in a multivalent way, this value does not present a single binding event of the dendron to Ad5, but point towards strong interactions between Ad5 and dendron.^19^ We observed green fluorescence by fluorescence microscopy for all cells treated with Ad5/dendron complexes whereas Ad5 alone led to low gene transduction (Figure 4 C; see Figure S27). Therefore, all dendron conjugates as well as the control dendrimer PPD3 bound to Ad5, and they transported Ad5 into cells by a CAR‐independent pathway, leading to EGFP‐expression. We quantified the gene transduction of Ad5 in low CAR cell line CHO‐K1 by flow cytometry by measuring the fluorescence intensity of EGFP. We found that gene transduction was enhanced when increasing the molar ratio of the dendron to Ad5 (Figure 4 D, see Figure S28). A double‐positive signal of EGFP and Cy5 was observed when infecting the cell with Ad5/**9 b** (see Figure S29). We found a significantly higher gene transduction of Ad5 when coated with four molar equivalents of **8** and **9 a** compared to coating with one molar equivalent of the sterically more demanding PPD3. The dendron **9 b** showed lower gene transduction efficiency than **8** and **9 a**, and it could be due to the attachment of the fluorophore that might also influence Ad5 binding and its cellular uptake. These results indicate that four molar equivalents of **8** and **9 a** increase Ad5 transport into these CAR‐negative cells compared to one molar equivalent of PPD3. As reported for amphiphilic PPDs,^19^ distinct amphiphilic surface patterns of the dendrons are crucial for biorecognition of Ad5. Even though the amphiphilic dendrons only represent one quarter of the full dendrimer PPD3, they retain both Ad5 binding capacity and gene transduction into CAR‐negative cells, while providing a second functionality for post‐modifications (Figures 4 A and 5 A).

In the next step, we studied whether the functionality at the focal point of the dendron was still accessible after complexation with Ad5. Consequently, we covalently modified the dendrons in situ by CuAAC on the Ad5 surface. The reaction was performed using **8** with 3‐azido‐7‐hydroxycoumarin, which is known to become highly fluorescent when forming a 1,2,3‐triazole product (Figure 5 A).^37^ As negative control, we used **9 a** attached to Ad5, which cannot undergo a CuAAC. We first investigated if the CuAAC proceeded in the micromolar range as well as the necessity of a ligand like TBTA to stabilize the copper(I) species.^38^ After 1 hour of incubation of **8** and 3‐azido‐7‐hydroxycoumarin with click reagents at a concentration of 10 μm dendron, we only found high fluorescence for the TBTA‐treated sample. This observation indicates the importance of adding a ligand like TBTA to the reaction mixture (see Figure S30). Additionally, it is reported that this class of ligands protect biomolecules from reactive oxygen species (ROS) during the ligation.^39^ For CuAAC on the Ad5 surface, we used the water‐soluble (4‐{[bis‐(1‐*tert*‐butyl‐1*H*‐[1,2,3]triazol‐4‐ylmethyl)‐amino]‐methyl}‐[1,2,3]triazol‐1‐yl)‐acetic acid (BTTAA), which is even more efficient in aqueous solutions than TBTA.^40^ Briefly, **8** was incubated with Ad5 for 1 hour, and unbound dendrons were removed by ultrafiltration. Subsequently, the CuAAC reagents were added. The negative control, **9 a**‐coated Ad5, was treated under the same reaction conditions. In addition, ultrafiltered **8** without Ad5 was prepared and click reagents were added to the supernatant to demonstrate that free dendrons could be removed by ultrafiltration. After incubation for 1 hour, fluorescence spectra were recorded (Figure 5 B). Since free 3‐azido‐7‐hydroxycoumarin is slightly fluorescent, we subtracted it as background. We observed a 21‐fold increase in relative fluorescence intensity at 477 nm compared to the biotin‐dendron coated Ad5 that served as negative control (Figure 5 C). These results indicate successful CuAAC at the focal points of **8** after formation of Ad5/dendron complexes. Therefore, we demonstrate that the functionalities of the dendron core are still accessible for post‐modifications even after complexation with Ad5. This model reaction proves that our structural dendron concept represents a promising tool for future applications in terms of in situ attachment of cell targeting groups or drug molecules.

## Conclusion

Ad5 is a common vector in gene therapy but its clinical usage has limitations because of the mistargeting of plasma‐protein‐coated Ad5 and acute toxicity. We present the synthesis of amphiphilic polyphenylene dendrons that bind to the surface of Ad5 through their polar and nonpolar surface groups and facilitate transport of the Ad5/dendron complexes into CAR‐negative cells. In this way, these dendrons maintained the crucial biorecognition features of the full dendrimer PPD3. As the dendrons form a new outer layer at the Ad5 surface, their interaction with blood plasma proteins might be crucial for future applications. Dendron‐coated liposomes were found to interact with specific proteins of the blood serum and plasma proteins such as vitronectin and ApoH, which could promote uptake into cancer and mesenchymal stem cells. In contrast to dendrimer PPD3, the dendrons provide an additional functionality for post‐modifications such as introducing either a fluorophore for imaging or d‐biotin as a bio‐orthogonal group. By desymmetrization of the dendrimer structure, we accessed a new platform for introducing bioactive groups to the Ad5 surface without the needing to covalently modify the virus particles. These reactive groups were accessible at the Ad5 surface as shown by CuAAC reactions. Moreover, this new concept of forming a supramolecular dendron corona at virus surfaces presents exciting opportunities for attaching, for example, cell targeting groups or drug molecules, and paves the way to rational control of Ad5 biodistribution to ultimately improve its capacity in virus‐assisted gene therapy.

## Conflict of interest

The authors declare no conflict of interest.

## Supporting information

As a service to our authors and readers, this journal provides supporting information supplied by the authors. Such materials are peer reviewed and may be re‐organized for online delivery, but are not copy‐edited or typeset. Technical support issues arising from supporting information (other than missing files) should be addressed to the authors.

Supplementary
