# Supplementary material for: Amphiphilic Polyphenylene Dendron Conjugates for Surface Remodeling of Adenovirus 5
Source: Angew Chem Int Ed Engl. 2020 Feb 3;59(14):5712–20. doi: 10.1002/anie.201913708 (PMC7155148; doi:10.1002/anie.201913708)
Supplement: Supplementary file 1 — Supplementary [file ANIE-59-5712-s001.pdf]

Angewandte Chemie
Eine Zeitschrift der Gesellschaft Deutscher Chemiker

Supporting Information

Amphiphilic Polyphenylene Dendron Conjugates for Surface Remodeling of Adenovirus 5

Jessica Wagner, Longjie Li, Johanna Simon, Lea Krutzke, Katharina Landfester, Volker Mailänder, Klaus Müllen, David Y. W. Ng, Yuzhou Wu,* and Tanja Weil**

anie_201913708_sm_miscellaneous_information.pdf

Table of Contents

1 Syntheses....4
1.1 Materials....4
1.2 Instruments....4
1.3 Synthesis of building blocks....5
1.4 Synthesis of PPD3....11
1.5 Synthesis of dendron conjugates....12
1.6 NMR-Spectra of key derivatives and final dendrons....24
1.7 MALDI-TOF mass spectra of final dendrons....30
2 Interaction of dendrons with serum proteins....31
2.1 Synthesis of liposomes....31
2.2 Synthesis of polystyrene nanoparticles....32
2.3 Coating of liposomes and nanoparticles with dendron 8 or dendrimer....32
2.4 Human plasma/serum....33
2.5 Protein corona preparation....33
2.5.1 Pierce assay....33
2.5.2 SDS PAGE....33
2.5.3 In solution digestion....34
2.5.4 Liquid chromatography coupled to mass spectrometry (LC-MS analysis)....34
2.6 Protein Corona: Supplementary figures....35
2.7 Interaction of Amphiphilic PPDs with lipid monolayer....39
3 Cellular uptake and cytotoxicity studies....40
3.1 Materials and Instruments....40
3.2 Methods....40
3.2.1 Cell culture....40

3.2.2 Cellular uptake of Cy5-dendron 9b 40

3.2.3 Cell viability/cytotoxicity–CellTiter-Glo® luminescent cell viability assay 41

4 Adenovirus 5 studies 42

4.1 Materials 42

4.2 Instruments 42

4.3 Methods 42

4.3.1 Complex formation and characterization of dendron/Ad5 interaction 42

4.3.2 Dynamic light scattering (DLS) and zeta potential 43

4.3.3 Transduction in CAR-negative cell line CHO-K1 48

4.3.4 Copper(I)-catalyzed azide-alkyne cycloaddition (CuAAC) on dendron coated Ad5 52

4.3.4.1 Investigating the importance of a ligand in CuAAC 52

4.3.4.2 CuAAC on Ad5/dendron complexes 53

4.3.5 Kinetic binding analysis[14] 54

References 56

Appendix: All identified Corona proteins 57

1 Syntheses

1.1 Materials

All chemicals were purchased from commercial sources (Sigma Aldrich, Acros Organics, Fisher Scientific, Thermo Scientific, TCI, Chempur, PurePEG etc.) and were used without any further purification. The organic solvents (ethyl acetate, dichloromethane (DCM), cyclohexane, *o*-xylene, dimethylformamide (DMF), dimethylsulfoxide (DMSO), 1,4-dioxane, methanol, tetrahydrofuran (THF), toluene) were obtained from Fisher Scientific or Acros Organics and used without further purification (HPLC grade). H_2O for reactions and purification was purified by a Merck Millipore purification system. Thin-layer chromatography (TLC) was performed on Alugram Sil G/UV254 plates from Macherey-Nagel and substances were detected under UV light at 254 nm or 366 nm. Column chromatography was performed applying Macherey Nagel silica gel with particle size of 0.04-0.063 mm or 0.063-0.2 mm. Size-exclusion chromatography was carried out using Sephadex® LH-20 in DMF.

1.2 Instruments

Nuclear Magnetic Resonance Spectroscopy (NMR). ^{1}H-NMR and ^{13}C-NMR were recorded on a Bruker Avance III 300 MHz, Avance III 500 MHz or Avance III 700 MHz spectrometer in deuterated solvents like CD_2Cl_2, MeOD and DMSO-d_6. ^{13}C-NMR were recorded in j-modulated spin-echo (JMOD) mode. Spectra were analyzed in either MestReNova or Topspin.

Matrix-assisted Laser Desorption/Ionization–Time of Flight Mass Spectrometry (MALDI-TOF). MALDI-TOF mass spectra were measured on a Bruker rapifleX MALDI-TOF/TOF and a Waters MALDI Synapt G2-SI. Dendron intermediates (before sulfonic acid deprotection) were dissolved in THF and measured by applying *trans*-2-[3-(4-*tert*-butylphenyl)-2-methyl-2-propenylidene]malononitrile (DCTB) as matrix. Deprotected dendrons were solvated in DMF and diluted in a saturated α-cyano-4-hydroxycinnamic acid (CHCA) solution in water/acetonitrile (1:1) + 0.1% TFA. Processing of data was performed in mMass.

Atmospheric Pressure Chemical Ionization Mass Spectrometry (APCI-MS). APCI mass spectra of precursors were recorded on an Advion expression-L Compact Mass Spectrometer (CMS) (Advion Inc. 61 Brown Rd, Suite 100, Ithaca, NY 14850, USA) by either measuring the sample with an atmospheric solid analysis probe (ASAP) or directly from TLC plates by an automated TLC plate reader (Plate express).

Field Desorption Mass Spectrometry (FD-MS). FD mass spectra of precursors were recorded on a VG Instruments ZAB 2-SE-FPD using an 8 kV accelerating voltage.

1.3 Synthesis of building blocks

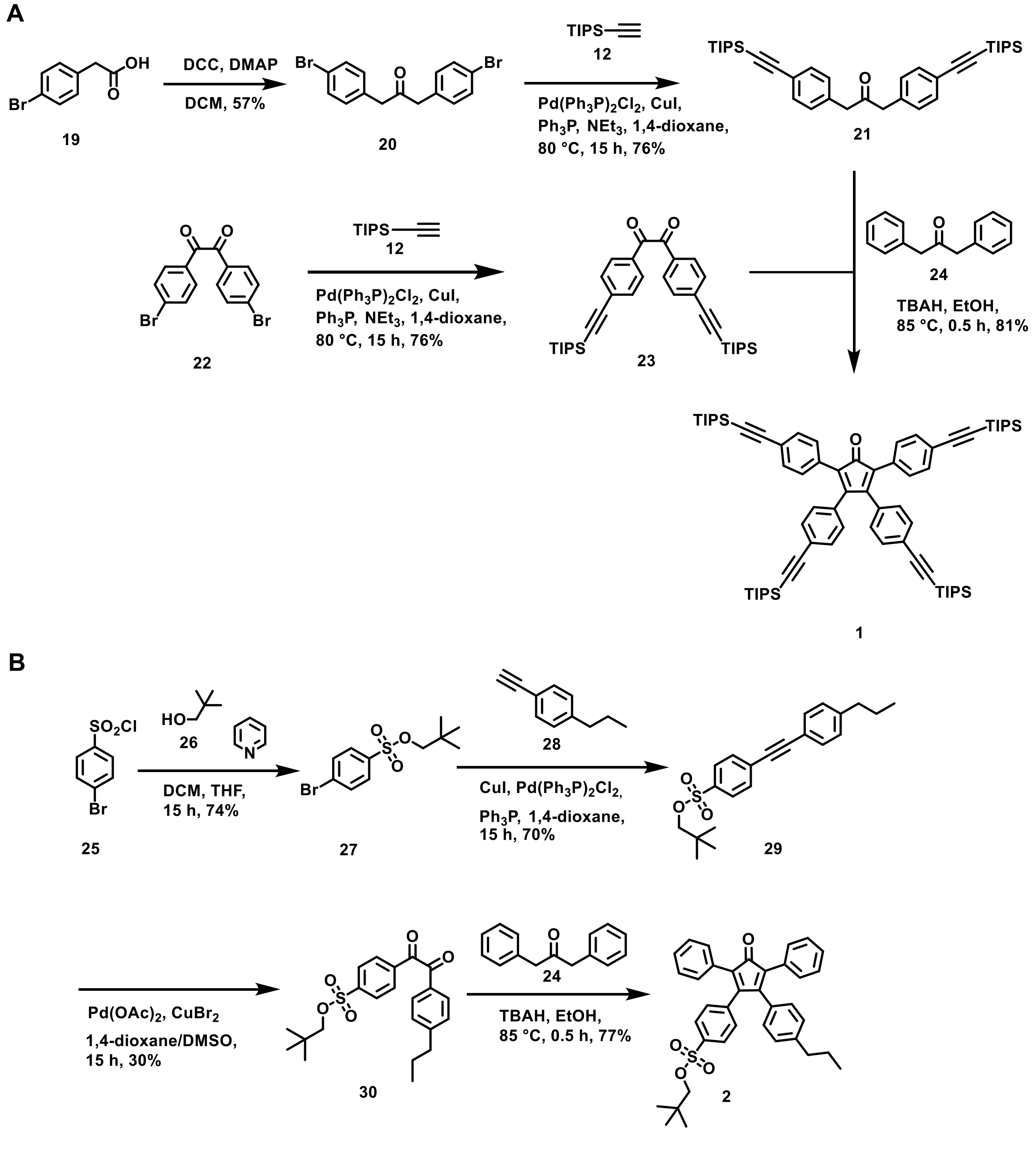

Figure S1. Reaction scheme of building blocks. (**A**) AB4 building block **1**, synthesized based on modified protocols from Morgenroth *et al*.[1] and (**B**) synthesis of surface building block **2** based on modified protocols from Stangenberg *et al*.[2]

1,3-Bis(4-bromophenyl)propan-2-one (**20**)

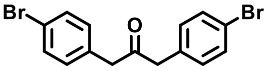

20

1,3-Bis(4-bromophenyl)propan-2-one (**20**) was synthesized according to the literature with modified protocol for purification.[3] Briefly, in a dry two neck round-bottom flask equipped with a dropping funnel dicyclohexylcarbodiimide (DCC) (9.6 g, 46.5 mmol) and 4-(dimethylamino)pyridine (DMAP) (1.42 g, 11.6 mmol) were dissolved in 100 mL dry dichloromethane. After degassing with argon for 30 min, *p*-bromophenylacetic acid (10.0 g, 46.5 mmol) in 100 mL dry dichloromethane was added dropwise and the reaction mixture was stirred for 24 h at room temperature. Then, the resulting *N,N'*-dicyclohexylurea was filtered off and the organic layer was washed with 10% hydrochloric acid and water. The crude product was purified by column chromatography using a mixture of cyclohexane and dichloromethane (1:2) to afford **20** as a white solid (4.85 g, 57%). All spectral data was in agreement with the literature.[3]
^{1}H NMR (300 MHz, CD_2Cl_2): δ(ppm) = 7.52–7.47 (m, 4H), 7.10–7.05 (m, 4H), 3.75 (s, 4H).
^{13}C NMR (75 MHz, CD_2Cl_2): δ(ppm) = 204.60, 133.62, 132.18, 131.90, 121.47, 48.95.
FD-MS: *m/z* calcd. for $C_{15}H_{12}Br_2O$ 368.1, found 369.6 $[M+H]^+$.

General procedure P1 for Sonogashira-Hagihara coupling:

The synthesis of ethynylated aryl compounds was modified from previously reported methods.[2, 4] Aromatic bromo compound (1 equiv), ethynyl derivative (1.1 equiv per bromine on the bromo compound) and triphenylphosphine (0.1 equiv) were dissolved in a mixture of 1,4-dioxane and triethylamine (2:1, 40 mL per gram bromo compound). After degassing with argon for 30 min, bis(triphenylphosphine)-palladium(II)chloride ($Pd(Ph_3)P)_2Cl_2$) (0.05 equiv) and copper iodide (0.1 equiv) were added. The reaction mixture was stirred under reflux at 85 °C and argon atmosphere for 15 h. The reaction mixture was cooled to room temperature, the palladium catalyst was filtered off and the fitration residue was washed with dichloromethane. The solvents were removed *in vacuo* and the residue was dissolved in dichloromethane. The organic layer was washed with water, dried over sodium sulfate and the solvent was evaporated. The crude product was purified by column chromatography.

1,3-bis(4-((tri*iso*propylsilyl)ethynyl)phenyl)propan-2-one (**21**)

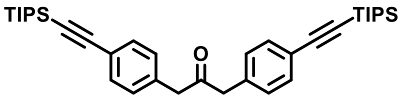

21

4,4'-Bromodiphenylacetone (**20**) (2.0 g, 5.4 mmol) was reacted with tri*iso*propylsilylacetylene (**12**) (2.48 g, 3.1 mL, 13.6 mmol) according to general procedure **P1**. The crude product was purified by silica gel column chromatography using a mixture of cyclohexane and dichloromethane (2:1) and compound **21** was recovered as a yellow solid (2.36 g, 76%). All spectral data was in agreement with the literature.[4]

^{1}H NMR (300 MHz, CD_2Cl_2): δ(ppm) = 7.44 (d, *J* = 8.4 Hz, 4H), 7.10 (d, *J* = 8.2 Hz, 4H), 3.74 (s, 4H), 1.14 (s, 42H).

^{13}C NMR (75 MHz, CD_2Cl_2): δ(ppm) = 204.84, 135.05, 132.66, 130.10, 122.79, 107.29, 91.32, 49.53, 18.99, 11.89.

FD-MS: *m/z* calcd. for $C_{37}H_{54}OSi_2$ 571.0, found 571.3 $[M]^{\bullet+}$.

1,2-bis(4-((tri*iso*propylsilyl)ethynyl)phenyl)ethane-1,2-dione (**23**)

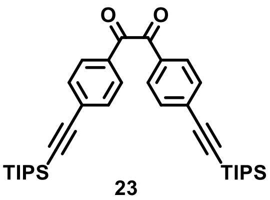

23

1,2-Bis(4-bromophenyl)ethane-1,2-dione (**22**) (10 g, 27.2 mmol) was reacted with tri*iso*propylsilyl-acetylene (**12**) (10.9 g, 13.4 mL, 59.8 mmol) according to general procedure **P1**. The crude product was purified by silica gel column chromatography using a mixture of cyclohexane and dichloromethane (7:3) to obtain compound **23** as a yellow solid (2.36 g, 76%).

^{1}H NMR (300 MHz, CD_2Cl_2): δ(ppm) = 7.95–7.84 (m, 4H), 7.64–7.55 (m, 4H), 1.14 (d, *J* = 2.6 Hz, 42H).

^{13}C NMR (75 MHz, CD_2Cl_2): δ(ppm) = 193.83, 132.96, 132.57, 130.74, 130.22, 106.27, 97.24, 18.94, 11.82.

FD-MS: *m/z* calcd. for $C_{36}H_{50}O_2Si_2$ 571.0, found 570.1 $[M-H]^{-}$.

Neopentyl-4-((4-propylphenyl)ethynyl)benzenesulfonate (**29**)

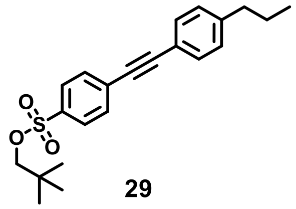

29

Neopentyl-4-bromobenzenesulfonate (**27**) (5 g, 16.3 mmol) was reacted with 1-ethynyl-4-propylbenzene (**28**) (2.58 g, 2.84 mL, 17.9 mmol) according to general procedure **P1**. The crude product was purified by silica gel column chromatography using a mixture of cyclohexane and dichloromethane (3:1) to obtain compound **29** as a white solid (4.21 g, 70%). All spectral data was in agreement with the literature.[2]
^{1}H NMR (300 MHz, CD_2Cl_2): δ(ppm) = 7.86 (d, J = 8.5 Hz, 2H), 7.69 (d, J = 8.5 Hz, 2H), 7.48 (d, J = 8.2 Hz, 2H), 7.21 (d, J = 8.2 Hz, 2H), 3.68 (s, 2H), 2.62 (t, J = 7.3 Hz, 2H), 1.65 (h, J = 7.3 Hz, 2H), 0.96 (t, J = 7.3 Hz, 3H), 0.89 (s, 9H).
^{13}C NMR (75 MHz, CD_2Cl_2): δ(ppm) = 145.01, 135.44, 132.57, 132.23, 129.81, 129.29, 128.46, 119.88, 94.15, 87.54, 80.51, 38.49, 32.09, 26.25, 24.93, 14.09.
FD-MS: *m/z* calcd. for $C_{22}H_{26}O_3S$ 370.5, found 370.7 $[M]^{\bullet+}$.

Neopentyl-4-bromobenzenesulfonate (**27**)

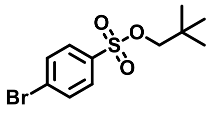

27

The synthesis of neopentyl-4-bromobenzenesulfonate (**27**) was modified from previously reported methods.[2] Briefly, in a dry two-neck flask neopentyl alcohol (**26**) (15.5 g, 176 mmol) was dissolved in dry dichloromethane and pyridine (7.66 g, 7.8 mL, 96.9 mmol) was added. The solution was cooled down to 0 °C and 4-bromobenzenesulfonyl chloride (**25**) dissolved in dry tetrahydrofuran (THF) (25 mL) was added dropwise. The resulting suspension was stirred at room temperature under argon atmosphere for 15 h. Pyridinium chloride was filtered off and the filtrate was reduced *in vacuo*. The crude product was purified by silica gel column chromatography using a mixture of cyclohexane and dichloromethane (1:2) to afford **27** as a white solid (19.9 g, 74%). All spectral data was in agreement with the literature.[2]

^{1}H NMR (300 MHz, CD_2Cl_2): δ(ppm) = 7.79–7.70 (m, 4H), 3.67 (s, 2H), 0.89 (s, 9H).
^{13}C NMR (75 MHz, CD_2Cl_2): δ(ppm) = 135.64, 133.12, 129.95, 129.27, 80.63, 32.08, 26.22.
FD-MS: *m/z* calcd. for $C_{11}H_{15}BrO_3S$: 307.2, found 308.7 $[M+H]^+$.

Neopentyl-4-((2-oxo-2(-4-propylphenyl)acetyl)benzenesulfonate (**30**)

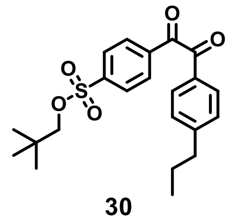

30

Neopentyl-4-((4-propylphenyl)ethynyl)benzenesulfonate (**29**) (2 g, 5.40 mmol) was solvated in 2 mL 1,4-dioxane and 15 mL dimethylsulfoxide were added. Subsequently, copper(II) bromide (121 mg, 0.54 mmol) and palladium(II) acetate (121 mg, 0.54 mmol) were added and the reaction mixture was stirred at 90 °C under argon atmosphere for 8 h. Then, water was added followed by extraction with ethyl acetate. The organic layer was dried over sodium sulfate and concentrated *in vacuo*. The crude product was purified by silica gel column chromatography using a mixture of cyclohexane and DCM (3:4) to afford **30** as a yellow oil (642 mg, 30%). All spectral data was in agreement with the literature.[2]
^{1}H NMR (300 MHz, CD_2Cl_2): δ(ppm) = 8.18–8.12 (m, 2H), 8.06–8.01 (m, 2H), 7.92–7.86 (m, 2H), 7.39–7.34 (m, 2H), 2.69 (t, J = 7.5 Hz, 2H), 1.75–1.58 (m, 2H), 0.95 (t, J = 7.3 Hz, 3H), 0.90 (s, 9H). ^{13}C-NMR (75 MHz, CD_2Cl_2): δ(ppm) 193.50, 152.23, 141.75, 137.34, 131.05, 130.79, 130.62, 129.90, 128.98, 81.04, 38.75, 32.15, 26.21, 24.71, 14.05.
FD-MS: *m/z* calcd. for $C_{22}H_{26}O_5S$: 402.5, found 403.2 $[M+H]^+$.

General procedure P2 for Knoevenagel reaction:

1,3-Diphenylacetone derivative (1 equiv) and benzil derivative (1 equiv) were dissolved in ethanol (~15 mL per 500 mg benzil) and heated up to 85 °C. Subsequently, 1 M methanolic tetrabutylammonium hydroxide (TBAH) solution (0.25 equiv) was added. After stirring at 85 °C for 0.5 h, the reaction mixture was diluted in dichloromethane. The organic layer was washed with water, dried over sodium sulfate and concentrated *in vacuo*. The crude product was purified by silica gel column chromatography.

2,3,4,5-tetrakis(4-((tri*iso*propylsilyl)ethynyl)phenyl)cyclopenta-2,4-dien-1-one (AB4 building block, **1**)

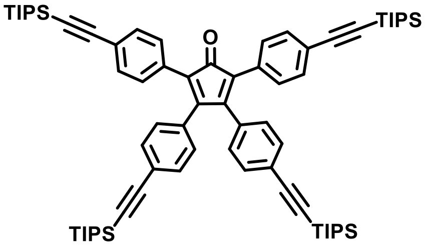

1

1,3-bis(4-((tri*iso*propylsilyl)ethynyl)phenyl)propan-2-one (**21**) (4 g, 7.01 mmol) was reacted with 1,2-bis(4-((tri*iso*propylsilyl)ethynyl)phenyl)ethane-1,2-dione (**23**) (4 g, 7.01 mmol) in 120 mL ethanol according to general procedure **P2**. The crude product was purified by silica gel column chromatography using a mixture of cyclohexane and THF (20:1) to afford **1** as a dark red solid (6.3 g, 81%). All spectral data was in agreement with the literature.

^{1}H NMR (300 MHz, CD_2Cl_2): δ(ppm) = 7.38–7.32 (m, 4H), 7.32–7.27 (m, 4H), 7.19–7.13 (m, 4H), 6.91–6.85 (m, 4H), 1.12 (d, J = 1.4 Hz, 84H).

^{13}C NMR (75 MHz, CD_2Cl_2): δ(ppm) = 199.52, 154.57, 132.31, 132.19, 131.15, 130.53, 129.85, 125.95, 124.52, 107.46, 107.02, 93.17, 92.51, 18.99, 11.87.

FD-MS: *m/z* calcd. for $C_{73}H_{100}OSi_4$ 1106, found 1108 $[M+2H]^{2+}$.

Neopentyl 4-(3-oxo-2,4-diphenyl-5-(4-propylphenyl)cyclopenta-1,4-dien-1-yl)benzenesulfonate (surface building block, **2**)

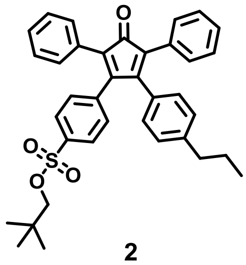

2

1,3-Diphenylacetone (**24**) (308 mg, 1.47 mmol) was reacted with neopentyl-4-((2-oxo-2(-4-propylphenyl)acetyl)benzenesulfonate (**30**) (590 mg, 1.47 mmol) in 10 mL ethanol according to general procedure **P2**. The crude product was purified by silica gel column chromatography using a mixture of cyclohexane and THF (4:1) to afford **2** as a dark purple solid (653 mg, 77%). All spectral data was in

agreement with the literature.[2]

^{1}H NMR (300 MHz, CD_2Cl_2): δ(ppm) = 7.70 (d, *J* = 8.5 Hz, 2H), 7.31–7.22 (m, 8H), 7.20–7.12 (m, 4H), 7.01 (d, *J* = 8.3 Hz, 2H), 6.83 (d, *J* = 8.2 Hz, 2H), 3.64 (s, 2H), 2.54 (t, *J* = 7.8, 7.2 Hz, 2H), 1.61 (dt, *J* = 13.7, 7.4 Hz, 2H), 0.92 (t, *J* = 7.3 Hz, 3H), 0.87 (s, 9H).

^{13}C NMR (75 MHz, CD_2Cl_2): δ(ppm) = 200.22, 155.08, 152.76, 144.50, 139.78, 135.83, 131.36, 130.72, 130.66, 130.32, 129.56, 128.84, 128.74, 128.56, 128.09, 127.61, 125.62, 80.56, 38.26, 32.05, 26.27, 24.77, 14.02.

FD-MS: *m/z* calcd. for $C_{37}H_{36}O_4S$: 576.7, found 576.8 $[M]^{\bullet+}$.

1.4 Synthesis of PPD3

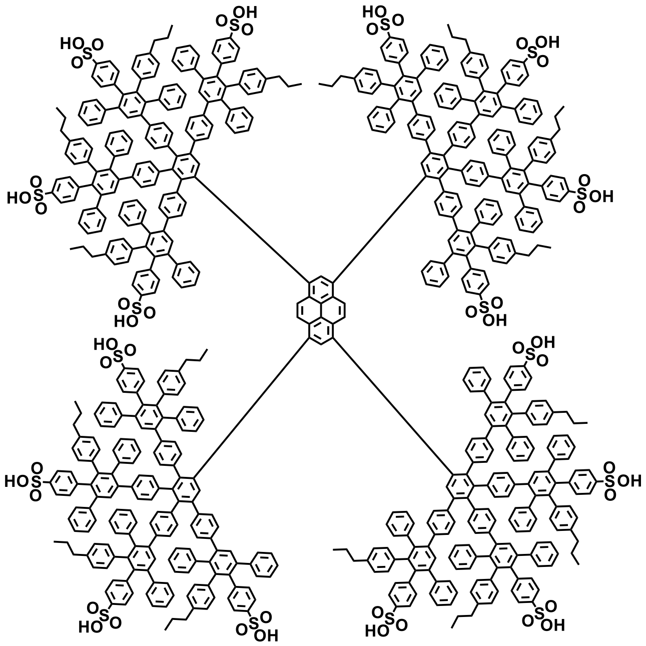

PPD3

PPD3 was synthesized in a divergent way as previously reported. All spectral data was in agreement with the literature.[2, 5]

^{1}H NMR (700 MHz, DMSO): δ(ppm) = 7.50 – 6.06 (m, 378H), 2.44 – 2.16 (m, 32H), 1.56 – 1.20 (m, 32H), 0.79 – 0.46 (m, 48H).

^{13}C NMR (176 MHz, DMSO): δ(ppm) = 162.80, 145.06–124.19, 120.10, 34.37, 23.53, 13.14.

MALDI-TOF: *m/z* calcd. for $C_{664}H_{506}O_{48}S_{16}$ 9766.26, found 9766.10 $[M]^{\bullet+}$.

1.5 Synthesis of dendron conjugates

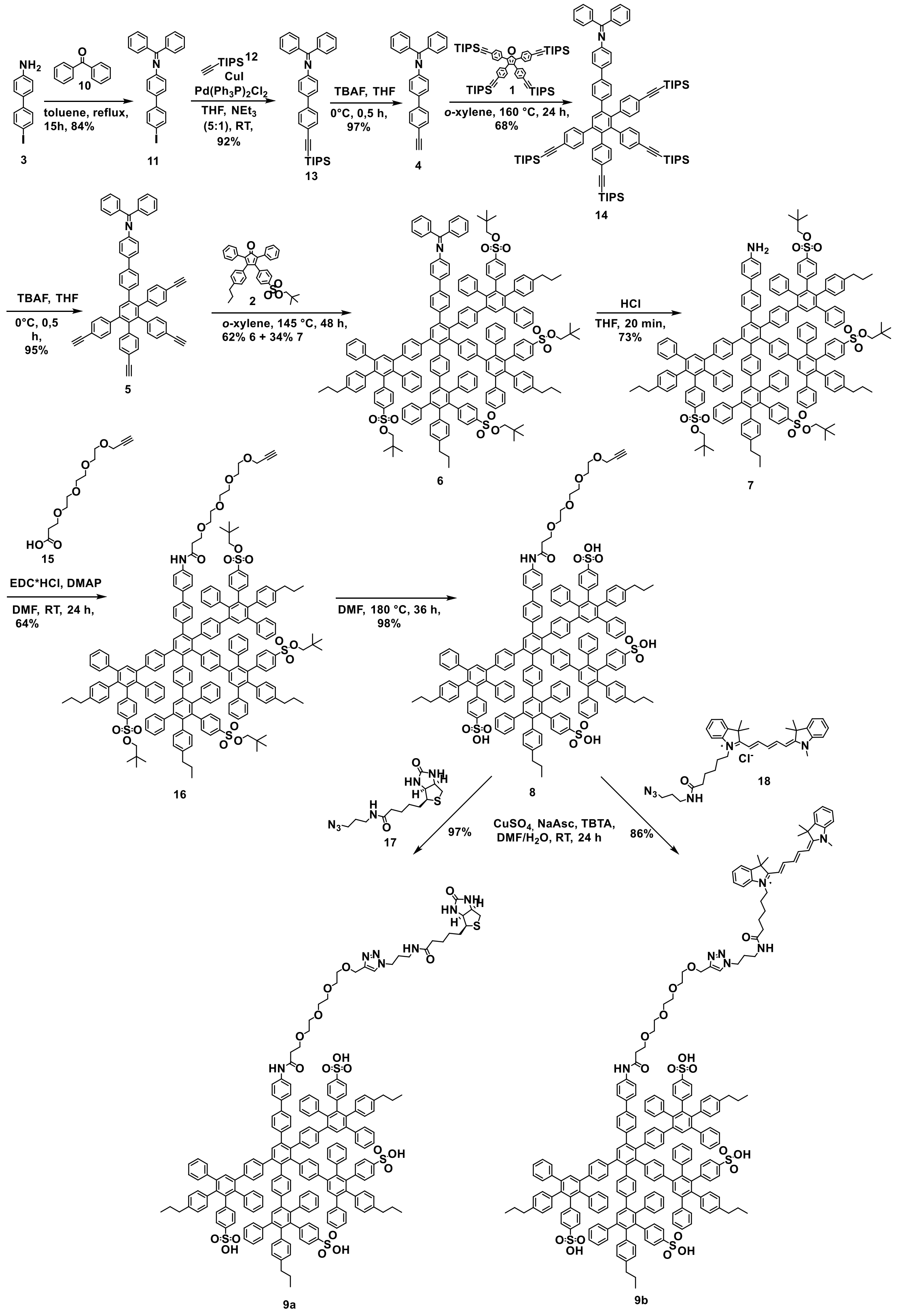

Figure S2. Detailed reaction scheme of dendron synthesis

N-(4'-iodo-[1,1'-biphenyl]-4-yl)-1,1-diphenylmethanimine (**11**)

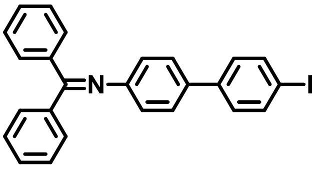

11

In a dry 25 mL Schlenk tube benzophenone (**10**) (100 mg, 549 µmol) and 4'-iodo-[1,1'-biphenyl]-4-amine (**3**) (178 mg, 604 µmol) were dissolved in 2 mL dry toluene and a 4 Å molecular sieve was added. The reaction mixture was heated under reflux for 24 h. Then, the molecular sieve was filtered off and washed with diethyl ether. Product **11** precipitated as a yellow solid (212 mg, 84%).
^{1}H NMR (500 MHz, CD_2Cl_2): δ(ppm) = 7.79–7.68 (m, 4H), 7.52–7.47 (m, 1H), 7.47–7.35 (m, 4H), 7.34–7.26 (m, 5H), 7.21–7.11 (m, 2H), 6.84–6.73 (m, 2H).
^{13}C NMR (126 MHz, CD_2Cl_2): δ(ppm) = 168.77, 151.76, 140.68, 140.06, 138.32, 136.78, 134.91, 131.36, 129.93, 129.88, 129.81, 129.16, 128.97, 128.71, 128.55, 127.34, 121.91, 92.77.
APCI-MS: *m/z* calcd. for $C_{25}H_{18}IN$ 459.1, found 459.5 $[M]^{\bullet +}$.

N-(4'-((tri*iso*propylsilyl)ethynyl)-[1,1'-biphenyl]-4-yl)1,1-diphenylmethanimine (**13**)

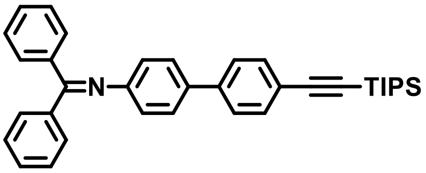

13

Imine **11** (300 mg, 0.65 mmol) and TIPS-acetylene (**12**) (137 mg, 169 µL, 0.75 mmol, 1.15 equiv) were dissolved in 10 mL THF and 2 mL triethylamine. After degassing, $Pd(Ph_3)P)_2Cl_2$ (45.8 mg, 65.3 µmol, 0.1 equiv) and copper iodide (24.9 mg, 131 µmol, 0.2 equiv) were added. The reaction mixture was stirred at room temperature under argon atmosphere for 15 h. Then, it was filtered and the filtrate was diluted with dichloromethane. The organic layer was washed with water, dried over sodium sulfate and purified by column chromatography using a mixture of cyclohexane and dichloromethane (1:2) to obtain **13** as a yellow solid (309 mg, 92%).
^{1}H NMR (500 MHz, CD_2Cl_2): δ (ppm) = 7.78–7.71 (m, 2H), 7.53–7.46 (m, 5H), 7.46–7.38 (m, 4H), 7.31 (m, 3H), 7.20–7.12 (m, 2H), 6.79 (d, J = 8.1 Hz, 2H), 1.14 (s, 21H).

^{13}C NMR (126 MHz, CD_2Cl_2): δ (ppm) = 168.19, 151.14, 140.43, 139.50, 136.23, 134.63, 132.28, 130.77, 129.36, 129.23, 128.59, 128.13, 127.98, 126.22, 121.85, 121.33, 107.00, 91.10, 18.42, 11.34.
APCI: m/z calcd. for $C_{36}H_{39}N_{Si}$ 513.3, found 513.8 $[M]^{•+}$.

N-(4'-ethynyl-[1,1'-biphenyl]-4-yl)-1,1-diphenylmethanimine (**4**)

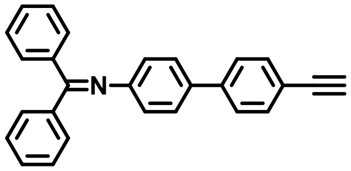

4

To an ice-cooled solution of **13** (155 mg, 302 µmol) in 24 mL dry THF 453 µL of a 1 M tetrabutyl-ammonium fluoride (TBAF) solution in THF (118 mg, 453 µmol, 1.5 equiv) were added. The reaction mixture was stirred at 0 °C under argon atmosphere for 0.5 h. The reaction was quenched by water addition, extracted with dichloromethane and dried over sodium sulfate. After evaporation of the solvents compound **4** was obtained as a yellow solid (105 mg, 97%).
^{1}H-NMR (500 MHz, CD_2Cl_2) δ(ppm) = 7.78–7.70 (m, 2H), 7.56–7.46 (m, 5H), 7.45–7.38 (m, 4H), 7.35–7.26 (m, 3H), 7.19–7.14 (m, 2H), 6.84–6.75 (m, 2H), 3.17 (s, 1H).
^{13}C-NMR (126 MHz, CD_2Cl_2) δ(ppm) = 168.80, 151.83, 141.54, 140.05, 136.78, 135.06, 133.01, 131.36, 129.94, 129.82, 129.17, 128.71, 128.55, 127.52, 126.93, 121.91, 120.95, 84.00, 78.07.
MALDI-TOF: *m/z* calcd. for $C_{27}H_{19}N$ 357.15, found 357.22 $[M]^{•+}$.

Terms for following dendron nomenclature

R-Core-Gx-$(abcd)_y$

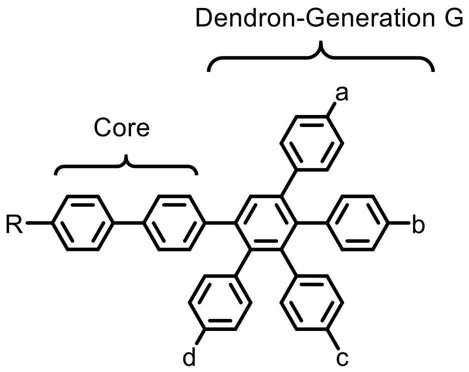

R = Functionality at the core
a,b,c,d = Surface functionalities
G = Generation
x = Generation number
y = Number of surface functions

Imine-biphenyl-G1-(ethinyl-TIPS)$_4$ **14**

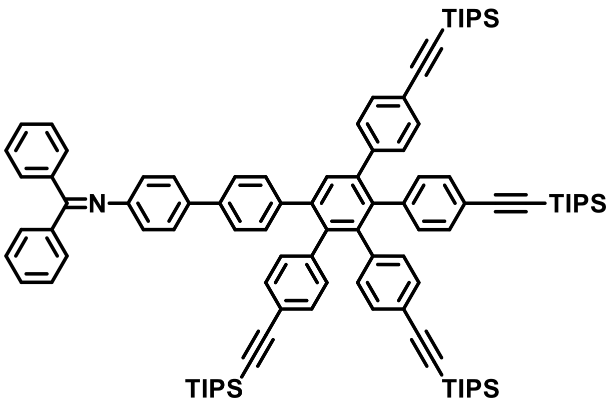

14

Dendron core **4** (30.0 mg, 83.9 µmol) and AB4-building block **1** (139 mg, 126 µmol, 1,5 equiv) were dissolved in 3 mL *o*-xylene and stirred at 160 °C in a sealed microwave tube under argon atmosphere for 24 h. After concentration *in vacuo* the crude product was purified by column chromatography using a mixture of cyclohexane and DCM (3:2) to obtain compound **14** as a yellow solid (82.4 mg, 68%).

^{1}H NMR (500 MHz, CD_2Cl_2): δ (ppm) = 7.77–7.70 (m, 2H), 7.52–7.45 (m, 2H), 7.44–7.36 (m, 6H), 7.34–7.25 (m, 5H), 7.18–7.03 (m, 12H), 6.84 (m, J = 8.2, 1.8 Hz, 4H), 6.79–6.73 (m, 4H), 1.15–1.04 (m, 84H).

^{13}C NMR (126 MHz, CD_2Cl_2): δ (ppm) = 168.66, 151.43, 142.10, 141.47, 141.40, 140.98, 140.83, 140.64, 140.48, 140.36, 140.13, 139.33, 139.13, 138.96, 136.83, 135.33, 132.23, 131.93, 131.84, 131.44, 131.40, 131.29, 131.19, 130.78, 130.34, 129.95, 129.80, 129.12, 128.69, 128.52, 127.32, 126.31, 122.24, 121.85, 121.69, 121.59, 121.46, 107.47, 107.40, 107.36, 91.66, 91.36, 91.24, 91.22, 18.97, 11.89.

MALDI-TOF: *m/z* calcd. for $C_{27}H_{19}N$ 1433.84, found 1433.73 $[M]^{\bullet+}$, 1456.72 $[M+Na]^+$, 1472.69 $[M+K]^+$.

Imine-biphenyl-G1-(ethinyl)$_4$ **5**

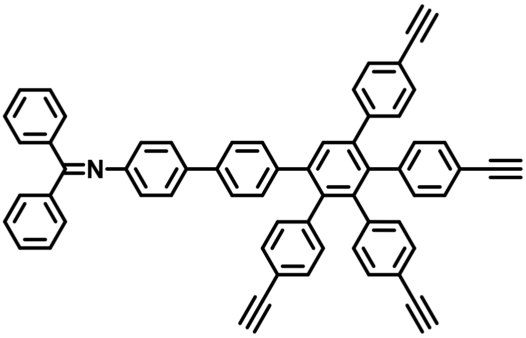

5

To an ice-cooled solution of dendron **14** (130 mg, 90.6 µmol) in 20 mL dry THF, 543 µL of a 1 M TBAF solution in THF (142 mg, 543 µmol, 6 equiv) were added. The reaction mixture was stirred at 0 °C under argon atmosphere for 0.5 h. The reaction was quenched by water addition, extracted with dichloromethane and dried over sodium sulfate. After evaporation of the solvents compound **5** was obtained as a yellow solid (69.6 mg, 95%).

^{1}H NMR (500 MHz, CD_2Cl_2): δ (ppm) = 7.77–7.71 (m, 2H), 7.56 (s, 1H), 7.48 (d, J = 7.4 Hz, 1H), 7.45–7.36 (m, 5H), 7.34–7.26 (m, 6H), 7.18–7.13 (m, 4H), 7.11 (dd, J = 8.3, 2.7 Hz, 6H), 7.07–7.03 (m, 2H), 6.88–6.80 (m, 4H), 6.78–6.73 (m, 4H), 3.15–2.99 (m, 4H).

^{13}C NMR (126 MHz, CD_2Cl_2): δ (ppm) = 168.68, 151.45, 142.39, 141.42, 141.39, 141.21, 141.04, 140.88, 140.84, 140.10, 139.28, 139.23, 138.89, 136.83, 135.31, 132.08, 132.01, 132.00, 131.91, 131.55, 131.53, 131.28, 130.79, 130.44, 129.95, 129.79, 129.13, 128.69, 128.53, 127.32, 126.33, 125.97, 121.85, 120.86, 120.28, 120.20, 120.02, 83.96, 83.90, 83.88, 83.80, 78.03, 77.77, 77.70, 77.66.

MALDI-TOF: *m/z* calcd. for $C_{63}H_{39}N$ 809.31, found 809.37 $[M]^{\bullet+}$.

Imine-biphenyl-G2-(PSpen)$_4$ **6**

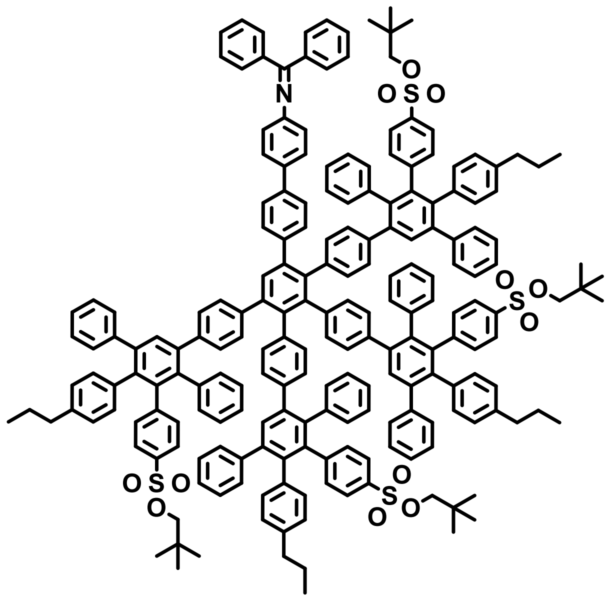

6

Imine-biphenyl-G1-(ethinyl)$_4$ dendron **5** (110 mg, 136 µmol) and surface building block **2** (627 mg, 1.09 mmol, 8 equiv) were dissolved in 10 mL *o*-xylene and stirred at 145 °C in a sealed Ace pressure tube under argon atmosphere for 48 h. After concentration *in vacuo* the crude product was purified by column chromatography using a mixture of cyclohexane and THF (3:1) to obtain imine-protected dendron **6** as a light brown solid (237 mg, 62 %) and amine dendron **7** as a brown solid (130 mg, 34%).

^{1}H NMR (700 MHz, CD_2Cl_2): δ (ppm) = 7.75 (d, J = 7.7 Hz, 2H), 7.56–7.31 (m, 21H), 7.28–7.22 (m, 4H), 7.20–7.02 (m, 30H), 7.01–6.95 (m, 6H), 6.91–6.63 (m, 42H), 6.58–6.52 (m, 2H), 6.50–6.40 (m, 4H), 3.44–3.31 (m, 8H), 2.43–2.31 (m, 8H), 1.51–1.45 (m, 8H), 0.85–0.72 (m, 48H).

^{13}C NMR (176 MHz, CD_2Cl_2): δ (ppm) = 151.37–125.82, 121.71, 80.25, 80.03, 79.96, 37.15, 25.73, 24.65, 13.56.

MALDI-TOF: *m/z* calcd. for $C_{207}H_{183}NO_{12}S_4$ 3002.26, found 3001.97 $[M]^{\bullet+}$, 3024.95 $[M+Na]^+$, 3040.95 $[M+K]^+$.

Amine-biphenyl-G2-(PSpen)$_4$ **7**

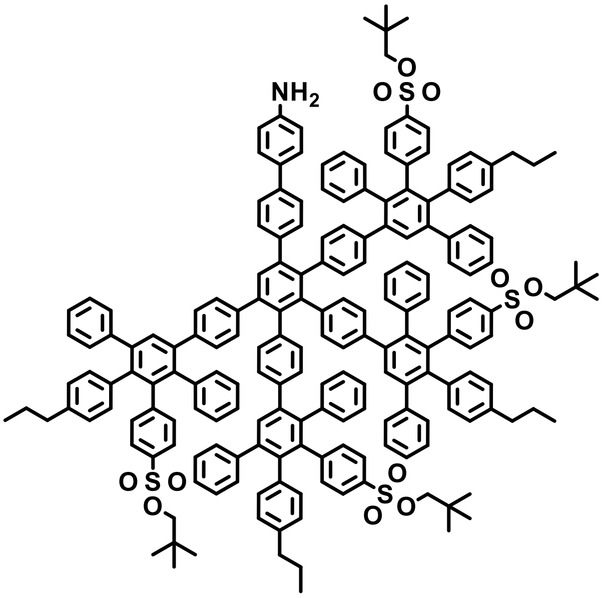

7

Imine-biphenyl-G2-(PSpen)$_4$ **6** (110 mg, 36.6 µmol) was dissolved in 3 mL THF and 1 mL 2 N hydrochloric acid were added. After stirring at room temperature under argon atmosphere for 5 min, 0.5 mL of concentrated hydrochloric acid were added and stirred for further 20 min. Then, ethyl acetate and water were added. The organic layer was separated, washed twice with water and dried over sodium sulfate. After concentration *in vacuo* the crude mixture was purified by column chromatography using a mixture of cyclohexane and THF (3:1) to afford compound **7** as a light brown solid (76 mg, 73%).

^{1}H NMR (700 MHz, CD_2Cl_2): δ (ppm) = 7.58–7.31 (m, 17H), 7.20–6.97 (m, 32H), 6.91–6.81 (m, 14H), 6.79–6.62 (m, 32H), 6.59–6.54 (m, 2H), 6.45 (dd, J = 13.1, 4.1 Hz, 4H), 3.46–3.31 (m, 8H), 2.43–2.28 (m, 8H), 1.52–1.44 (m, 8H), 0.85–0.71 (m, 48H).

^{13}C NMR (176 MHz, CD_2Cl_2): δ(ppm) = 151.99–125.64, 115.10, 80.04, 79.98, 37.67, 26.18, 24.63, 13.42.

MALDI-TOF: *m/z* calcd. for $C_{194}H_{175}NO_{12}S_4$ 2838.20, found 2838.01 $[M]^{\bullet+}$, 2860.97 $[M+Na]^+$, 2876.9625 $[M+2Na]^{2+}$.

Propargyl-TEG-amide-biphenyl-G2-(PSpen)$_4$ **16**

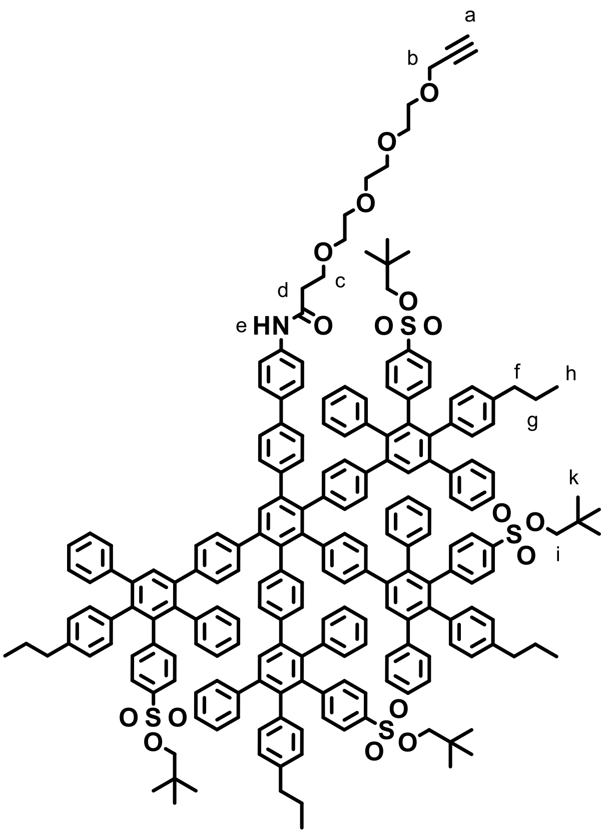

16

Amine-biphenyl-G2-(PSpen)$_4$ **7** (57.0 mg, 20.0 µmol) was dissolved in 3 mL DMF and propyne-*O*-(2-carboxyethyl)-*O′*-propargyl-triethylene glycol (**15**) (26.1 mg, 100 µmol, 5 equiv) in 0.5 mL DMF was added. Then, *N*-(3-dimethylaminopropyl)-*N′*-ethylcarbodiimide hydrochloride (EDC*HCl) (19.23 mg, 100 µmol) and 4-dimethylaminopyridine (DMAP) (7.35 mg, 60.2 µmol) were added. The reaction mixture was stirred at room temperature under argon atmosphere for 24 h. DMF was evaporated and the crude product was purified by silica gel chromatography using a mixture of DCM and methanol (20:1) to obtain Propargyl-TEG-amide-biphenyl-G2-(PSpen)$_4$ **16** as a light brown solid (40.1 mg, 64%).

^{1}H NMR (700 MHz, CD_2Cl_2): δ (ppm) = 8.60 (s, 1H,H_e), 7.64 (d, J = 8.3 Hz, 2H, H_{arom}), 7.58–7.30 (m, 17H, H_{arom}), 7.20–6.93 (m, 32H, H_{arom}), 6.92–6.79 (m, 14H, H_{arom}), 6.79–6.60 (m, 30H, H_{arom}), 6.57 (d, J = 7.0 Hz, 2H, H_{arom}), 6.45 (d, J = 17.4 Hz, 4H, H_{arom}), 4.15–4.08 (m, 2H, H_f), 3.82 (t, J = 5.6 Hz, 2H, H_c), 3.72–3.55 (m, 12H, H_{TEG}), 3.44–3.28 (m, 8H, H_i), 2.64 (t, J = 5.6 Hz, 2H, - H_d), 2.48–2.28 (m, 9H, H_a, H_f), 1.51–1.37 (m, 8H, H_g), 0.86–0.69 (m, 48H, H_h, H_k).

^{13}C NMR (176 MHz, CD_2Cl_2): δ (ppm) = 162.57, 147.74–120.66, 80.19, 80.12, 74.75, 74.41, 71.08, 70.97, 70.90, 70.88, 69.74, 67.65, 58.78, 38.61, 37.83, 26.29, 24.81, 13.64.

MALDI-TOF: *m/z* calcd. for $C_{206}H_{193}NO_{17}S_4$ 3080.32, found 3080.60 $[M]^{\bullet+}$, 3103.58 $[M+Na]^+$, 3119.41 $[M+2Na]^{2+}$.

Propargyl-TEG-amide-biphenyl-G2-(PS)$_4$ **8**

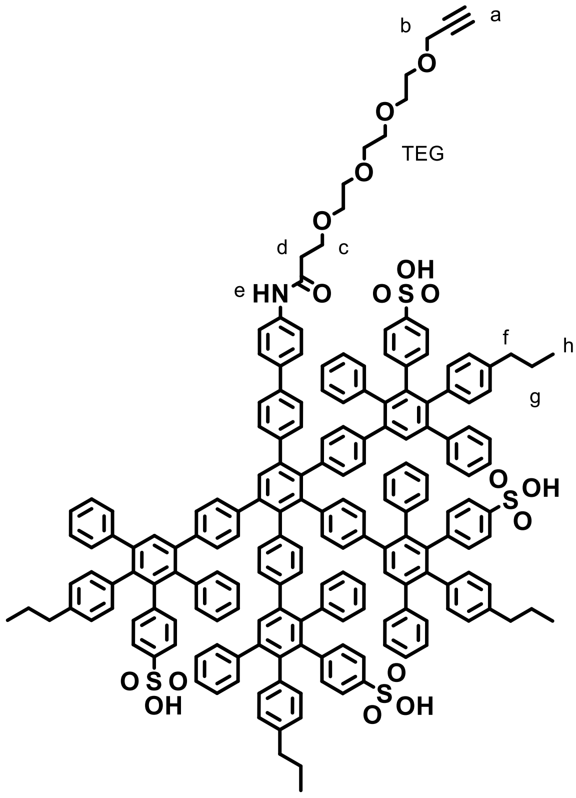

8

Propargyl-TEG-amide-biphenyl-G2-(PSpen)$_4$ (180 mg, 58.4 µmol) **16** was dissolved in 20 mL dry DMF, degassed with argon and stirred in a sealed Ace pressure tube at 180 °C for 36 h. Then, DMF was evaporated *in vacuo*, the residue was dissolved in methanol and precipitated in diethyl ether. After filtration propargyl-TEG-amide-biphenyl-G2-(PS)$_4$ **8** was obtained as a light brown solid (160 mg, 98%).

^{1}H NMR (500 MHz, DMSO-*d*$_6$): δ(ppm) = 10.03 (s, 1H, H_e), 7.75–7.56 (m, 4H, H_{arom}), 7.50–7.23 (m, 7H, H_{arom}), 7.22–6.54 (m, 84H, H_{arom}), 6.52–6.30 (m, 6H, H_{arom}), 4.11 (m, 2H, H_f), 3.71 (t, J = 5.6 Hz, 2H, H_c), 3.55–3.44 (m, 12H, H_{TEG}), 2.57 (t, J = 4.9 Hz, 2H, H_d), 2.44–2.23 (m, 9H, $H_{a,}$ H_f), 1.47–1.31 (m, 8H, H_g), 0.74–0.61 (m, 12H, H_h).

^{13}C NMR (126 MHz, DMSO): δ (ppm) = 145.54–119.32, 77.08, 69.75, 69.69, 69.47, 68.50, 66.67, 57.47, 36.44, 23.52, 13.11.

MALDI-TOF: *m/z* calcd. for $C_{186}H_{153}NO_{17}S_4$ 2800.00, found 2801.28 $[M+H]^+$, 2824.35 $[M+Na]^+$, 2840.31 $[M+K]^+$, 2862.25 $[M+NaK]^{2+}$, 2878.33 $[M+2K]^{2+}$.

Biotin-triazole-TEG-amide-biphenyl-G2-(PS)$_4$ **9a**

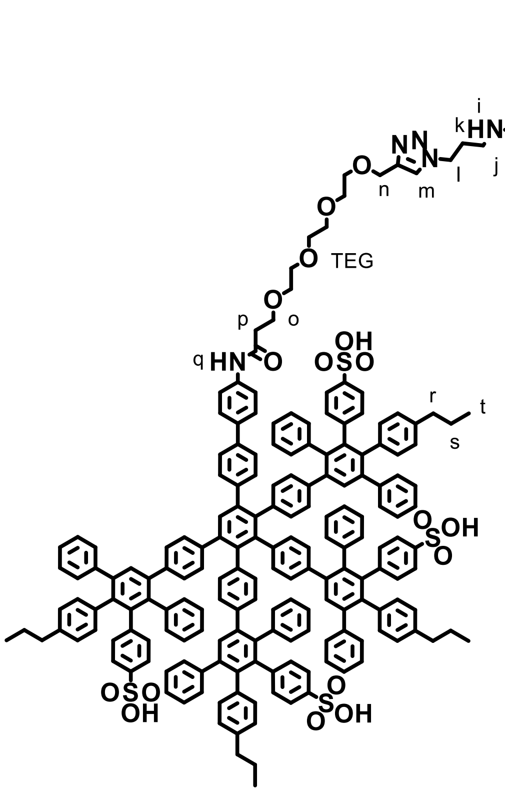

9a

Propargyl-TEG-amide-biphenyl-G2-(PS)$_4$ **8** (10 mg, 3.57 µmol) and azido-biotin derivative **17** (6.99 mg, 21.4 µmol) dissolved in each 0.5 mL DMF were combined and 1-(1-benzyltriazol-4-yl)-*N*,*N*-bis[(1-benzyltriazol-4-yl)methyl]methanamine (TBTA) (1.89 mg; 3.57 µmol) in 290 µL DMF was added. After degassing with argon, copper sulfate (0.57 mg, 3.57 µmol) in 136 µL ultrapure water and sodium ascorbate (1.41 mg, 7.14 µmol) in 224 µL ultrapure water were added. The reaction mixture was shaken at room temperature for 48 h under exclusion of light. The reaction mixture was purified via gel permeation chromatography (GPC) applying Sephadex LH-20 in DMF to obtain Biotin-triazole-TEG-amide-biphenyl-G2-(PS)$_4$ **9a** as a light brown solid (10.8 mg, 97%).

^{1}H NMR (700 MHz, DMSO-*d*$_6$) δ(ppm) = 10.03 (s, 1H, H_q), 8.08 (d, J = 3.0 Hz, 1H, H_{arom}), 7.88 (t, J = 4.5 Hz, 1H, H_i), 7.73–7.66 (m, 2H, H_{arom}), 7.60 (s, 2H, H_{arom}), 7.48–6.27 (m, 100H, H_{arom}, H_a, $H_{a'}$, H_m), 4.49 (s, 2H, H_n), 4.32 (t, J = 7.0 Hz, 2H, H_l), 4.28 (t, J = 6.5 Hz, 1H, $H_{b'}$), 4.13–4.09 (m, 1H, H_b), 3.71 (t, J = 6.2 Hz, 2H, H_o), 3.58–3.44 (m, 12H, H_{PEG}), 3.11–3.06 (m, 1H, H_d), 3.02 (q, J = 6.4 Hz, 2H, H_j), 2.79 (dd, J = 12.5, 5.1 Hz, 1H,H_c), 2.64–2.53 (m, 3H, $H_{c'}$, H_p), 2.45–2.22 (m, 8H, H_r), 2.06 (t, J = 7.5 Hz, 2H, H_h), 1.92 (p, J = 6.7 Hz, 2H, H_k), 1.68–1.23 (m, 14H, H_e, H_f, H_g, H_s), 0.74–0.62 (m, 12H, H_t).

^{13}C NMR (176 MHz, DMSO) δ(ppm) = 172.19, 162.68, 145.51-119.31, 69.76, 69.66, 68.95, 66.64, 63.53,

61.02, 59.19, 55.39, 47.15, 40.02,37.23, 36.44, 35.64, 35.17, 33.90, 33.60, 29.95, 28.19, 28.02, 25.23, 23.52, 13.12.

MALDI-TOF: *m/z* calcd. for $C_{199}H_{175}N_7O_{19}S_5$ 3126.15, found 3149.65 $[M+Na]^+$, 3171.62 $[M+2Na]^{2+}$, 3187.58 $[M+K]^+$, 3193.61 $[M+3Na]^{3+}$.

Cyanine 5-triazole-TEG-amide-biphenyl-G2-$(PS)_4$ **9b**

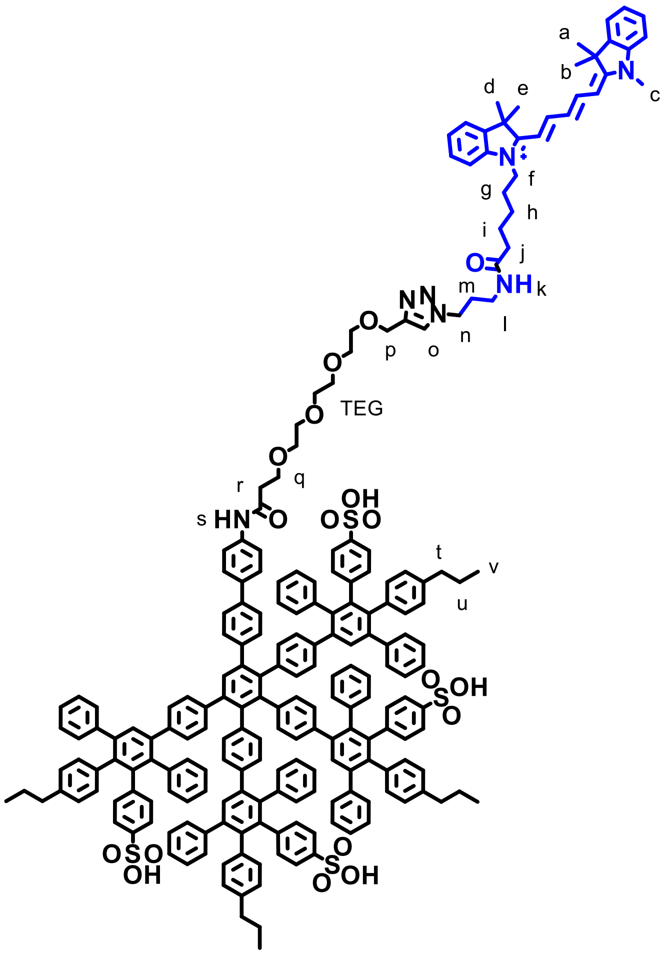

9b

Propargyl-TEG-amide-biphenyl-G2-$(PS)_4$ **8** (6.00 mg, 2.14 µmol) and Cy5-azide derivative **18** (3.86 mg, 6.42 µmol) dissolved in each 0.3 mL DMF were combined and TBTA (1.14 mg; 2.14 µmol) in 100 µL DMF was added. After degassing with argon, copper sulfate (0.34 mg, 2.14 µmol) in 46 µL ultrapure water and sodium ascorbate (0.85 mg, 4.28 µmol) in 54 µL ultrapure water were added. The reaction mixture was shaken at room temperature for 48 h under exclusion of light. The reaction mixture was purified via GPC applying Sephadex LH-20 in DMF to obtain Cyanine 5-triazole-TEG-amide-biphenyl-

G2-$(PS)_4$ **9b** as a blue solid (6.2 mg, 86%).

^{1}H NMR (700 MHz, DMSO-d_6) δ(ppm) = 10.00 (s, 1H, H_s), 8.35–8.23 (m, 2H, H_{arom}), 7.89–7.81 (m, 1H, H_k), 7.65 (d, J = 7.4 Hz, 2H, H_{arom}), 7.53 (dd, J = 14.7, 7.4 Hz, 2H, H_{arom}), 7.43–6.15 (m, 117H, H_{arom}), 4.45 (s, 2H, H_p), 4.26 (t, J = 7.0 Hz, 2H, H_n), 4.05–3.96 (m, 2H, H_f), 3.70–3.64 (m, 2H, H_r), 3.54–3.40 (m, 15H, $H_{TEG,}$ H_c), 2.98–2.93 (m, 2H, H_l), 2.35–2.22 (m, 8H, H_t), 2.00 (t, J = 7.1 Hz, 2H, H_j), 1.84 (q, J = 7.0 Hz, 2H, H_m), 1.64–1.54 (m, 12H, $H_{a,}$ $H_{b,}$ $H_{d,}$ H_e), 1.52–1.44 (m, 2H, H_i), 1.43–1.16 (m, 12H, $H_{g,}$ $H_{h,}$ H_u), 0.72–0.54 (m, 12H, H_v).

^{13}C NMR (176 MHz, DMSO) δ(ppm) = 154.33, 145.52–124.18, 123.88, 122.29, 118.68, 111.00, 69.80, 69.72, 68.50, 65.75, 63.57, 48.85, 47.15, 36.55, 36.45, 35.64, 34.40, 29.93, 27.11, 26.94, 24.84, 23.54, 13.14, 12.88.

MALDI-TOF: *m/z* calcd. for $C_{221}H_{198}N_7O_{18}S_4^+$ 3365.37, found 3365.28 $[M]^{\bullet+}$, 3387.26 $[M+Na]^+$, 3410.25 $[M+2Na]^{2+}$.

1.6 NMR-Spectra of key derivatives and final dendrons

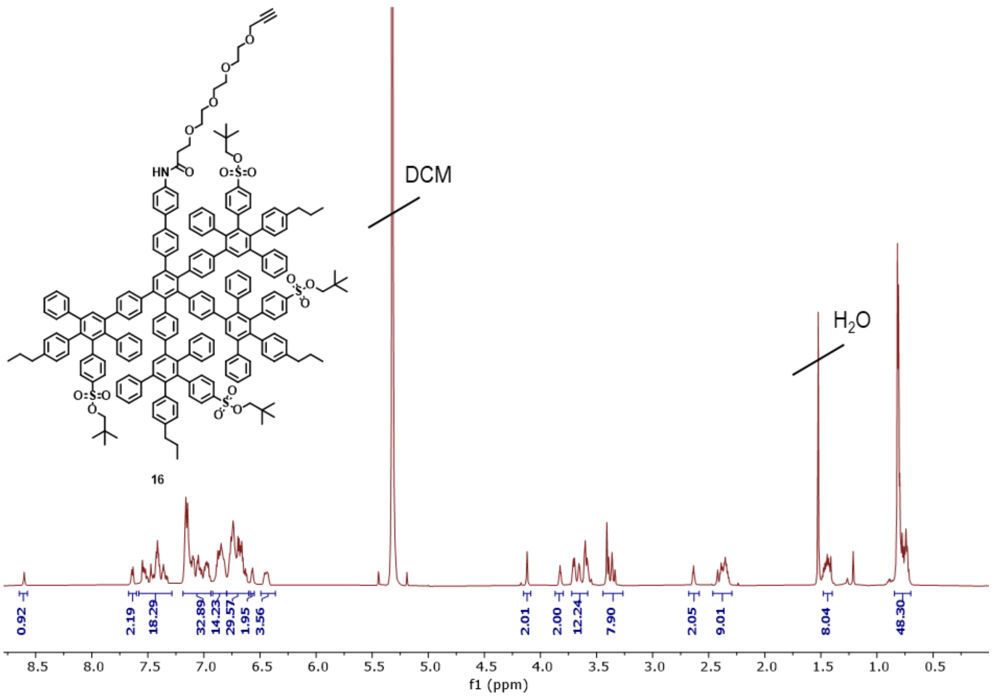

Figure S3. ^{1}H NMR spectrum (700 MHz) of protected dendron **16** recorded in deuterated dichloromethane.

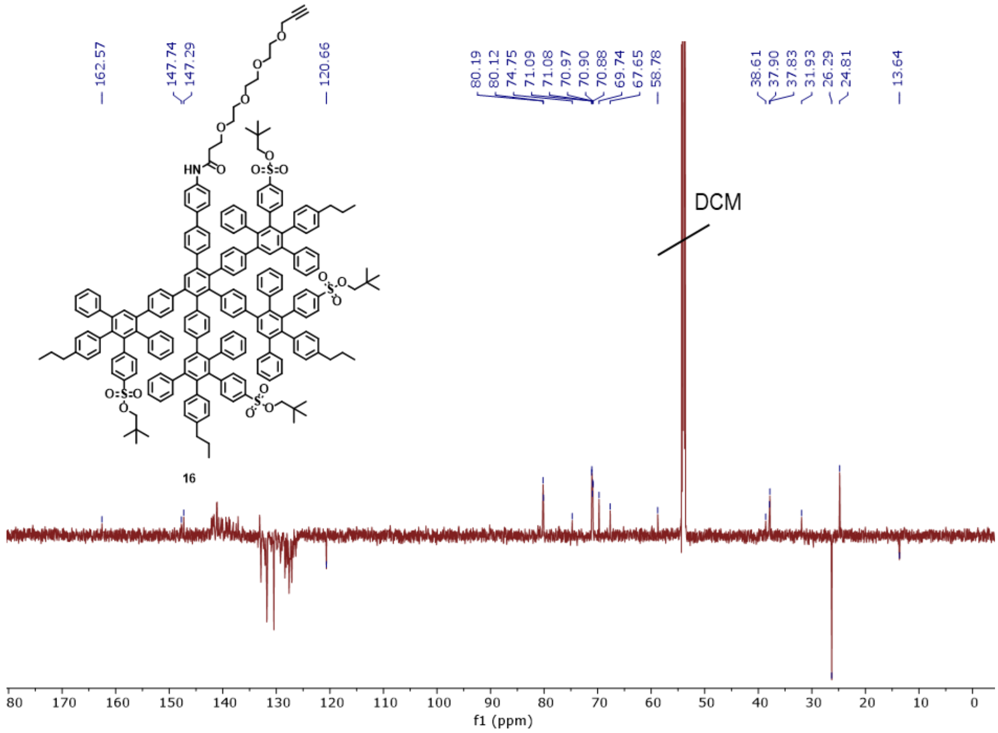

Figure S4. ^{13}C NMR spectrum (176 MHz) of protected dendron **16** recorded in deuterated dichloromethane.

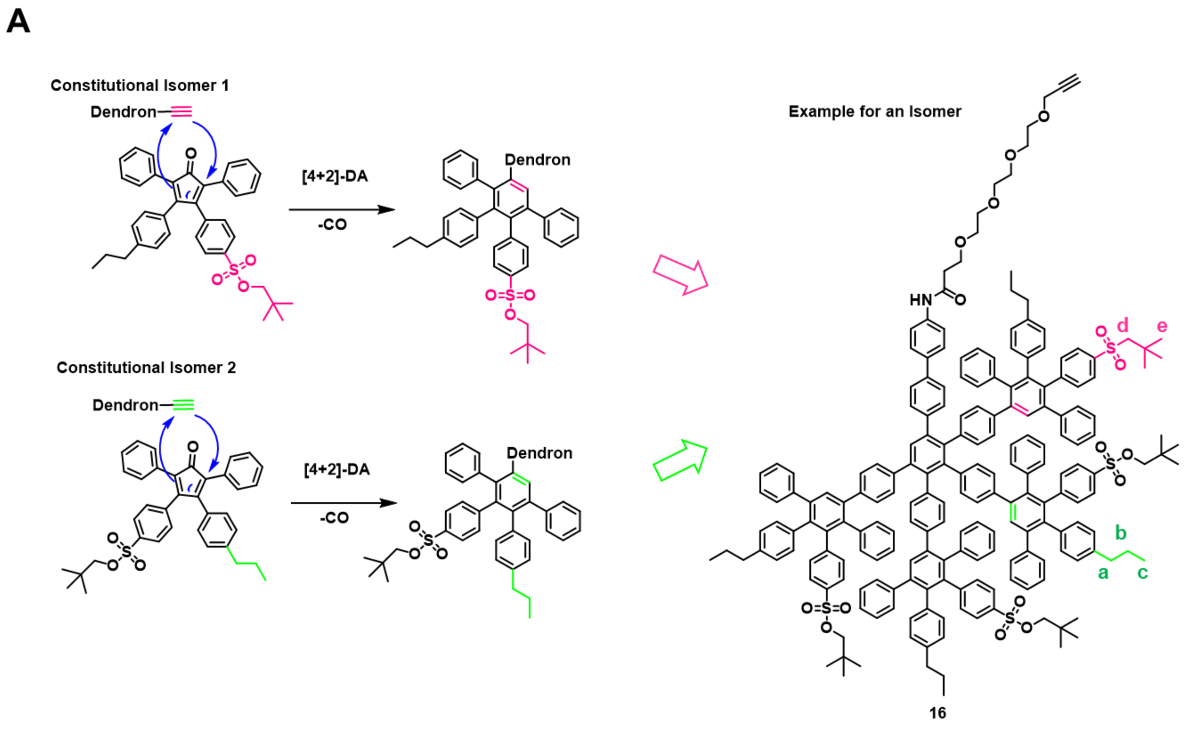

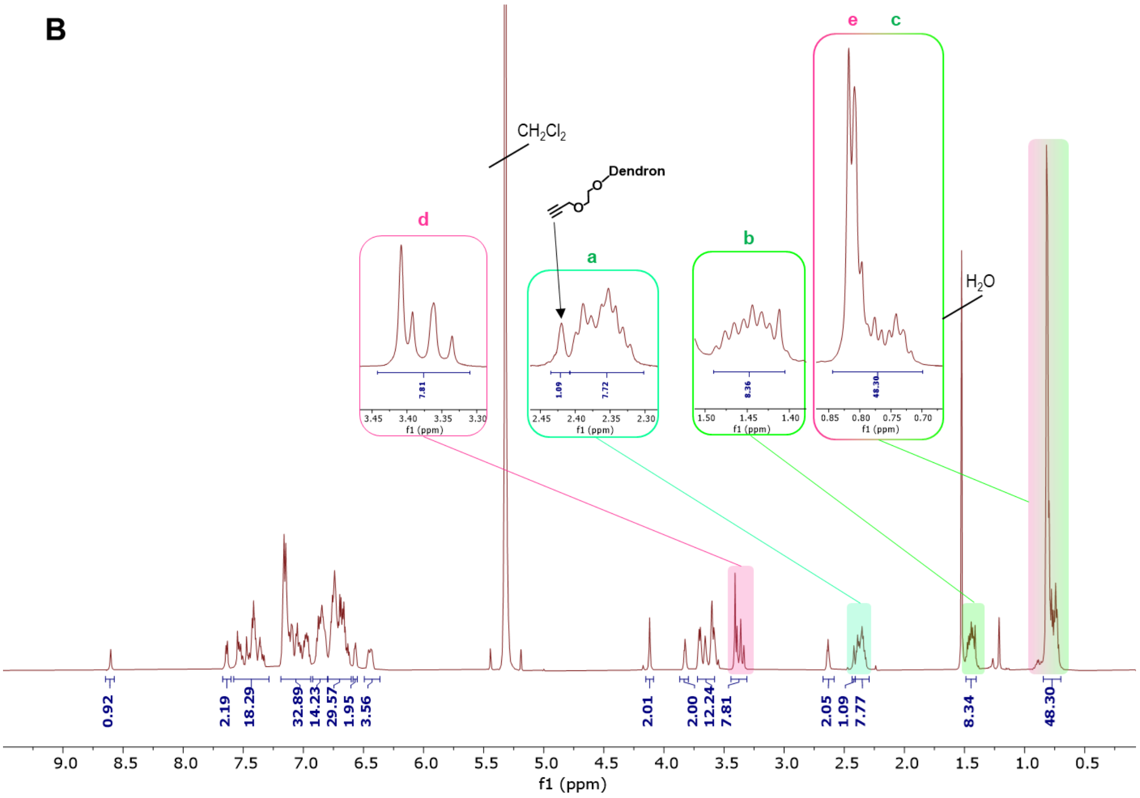

Figure S5. Constitutional isomers of deprotected dendron **16**. (**A**) shows the possibility to form two different constitutional isomers in each [4+2]-Diels-Alder reaction. (**B**) Constitutional isomers can be followed by ^{1}H NMR spectroscopy. The signal of the neopentyl-CH_2 group splits from a singlet to 4 signals with differences in intensity.

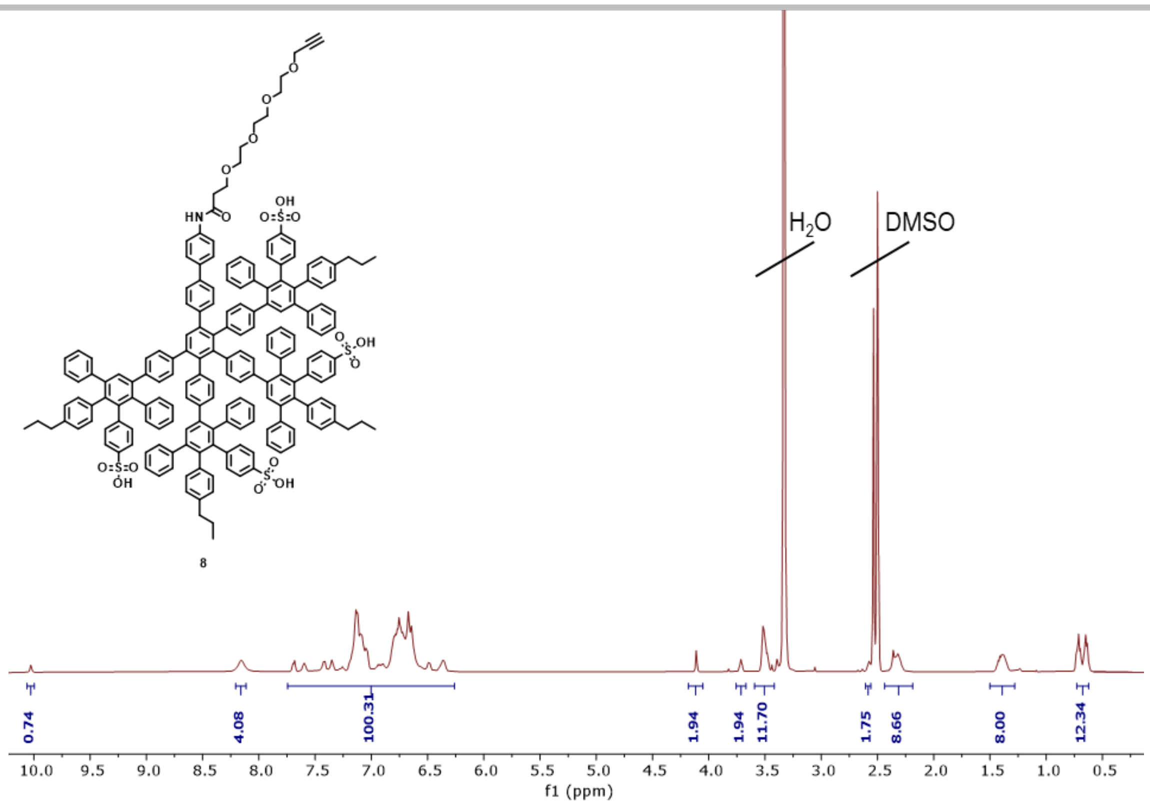

Figure S6. ^{1}H NMR spectrum (500 MHz) of deprotected dendron **8** recorded in deuterated dimethyl sulfoxide.

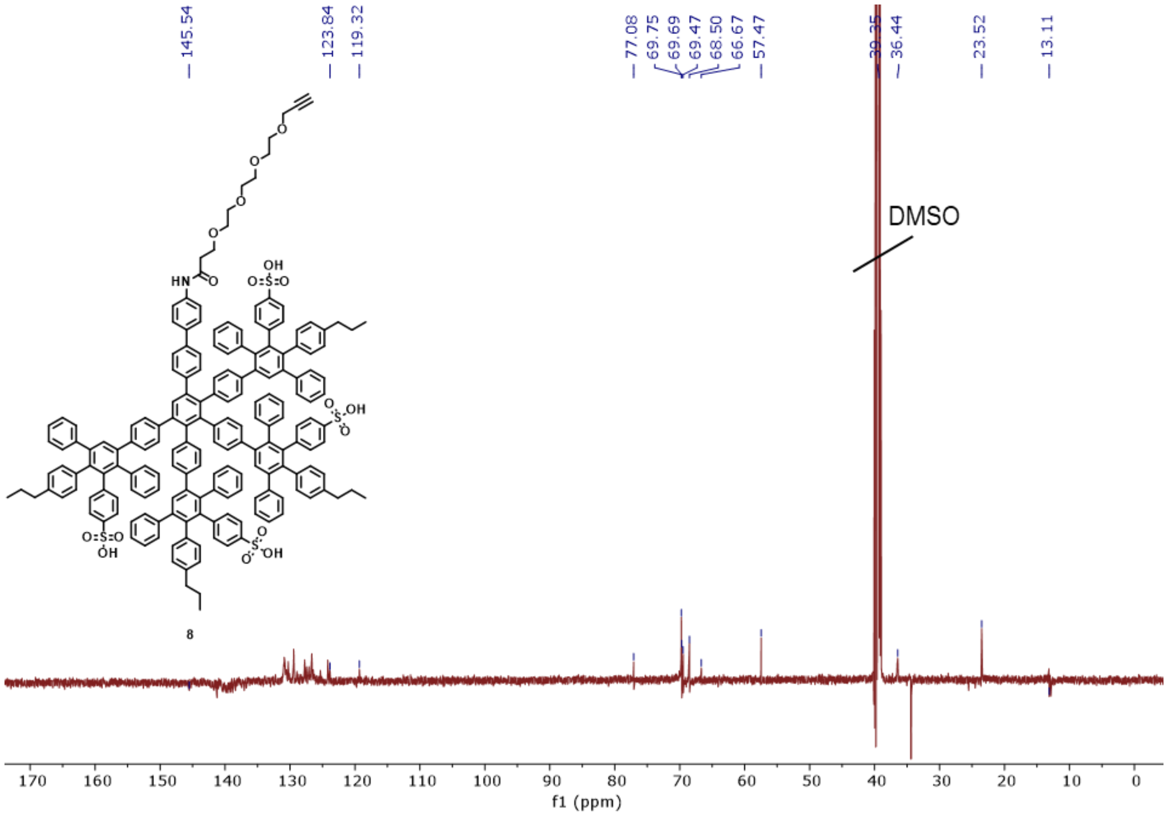

Figure S7. ^{13}C NMR spectrum (126 MHz) of protected dendron **8** recorded in deuterated dimethyl sulfoxide.

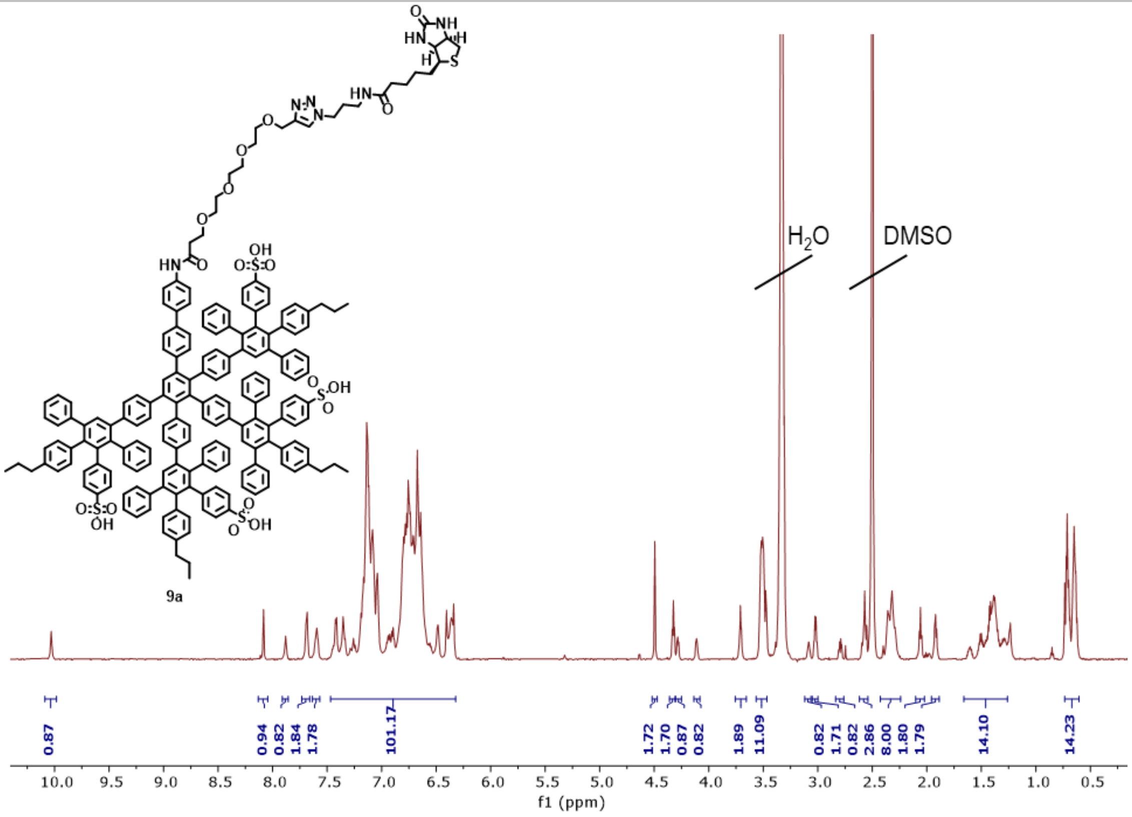

Figure S8. ^{1}H NMR spectrum (700 MHz) of biotin-dendron **9a** recorded in deuterated dimethyl sulfoxide.

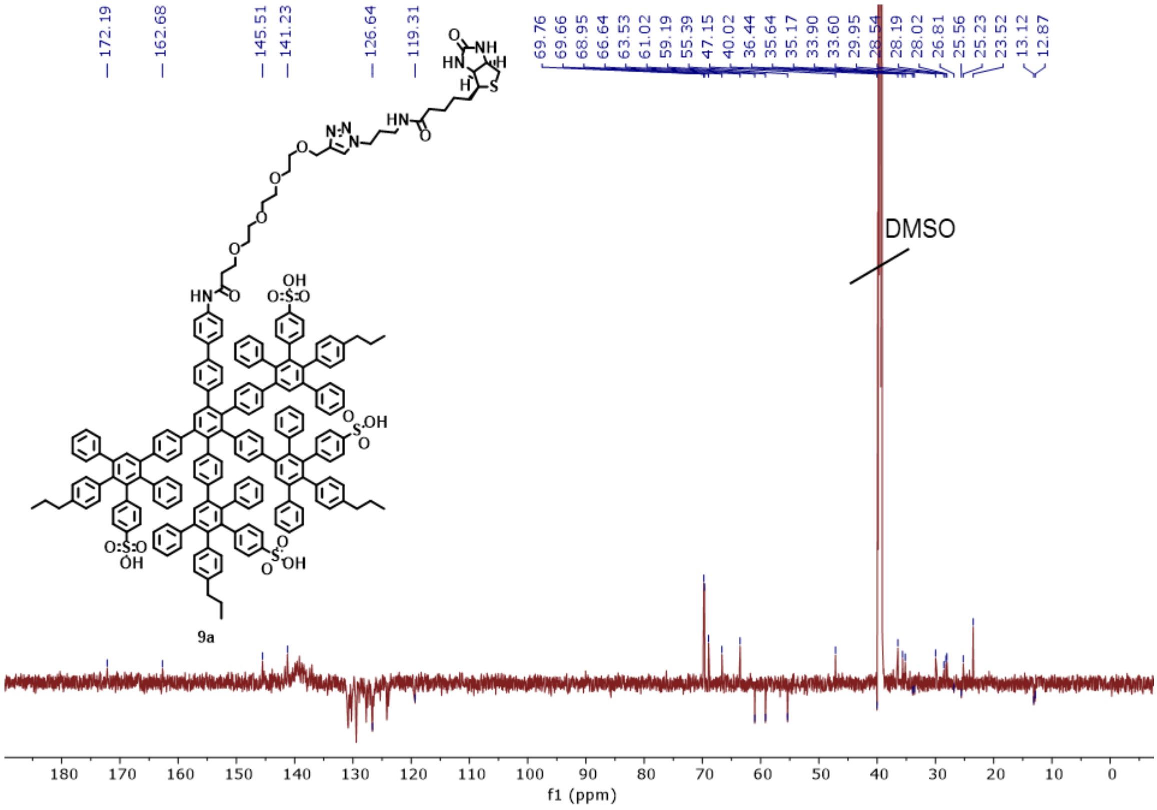

Figure S9. ^{13}C NMR spectrum (176 MHz) of biotin-dendron **9a** recorded in deuterated dimethyl sulfoxide.

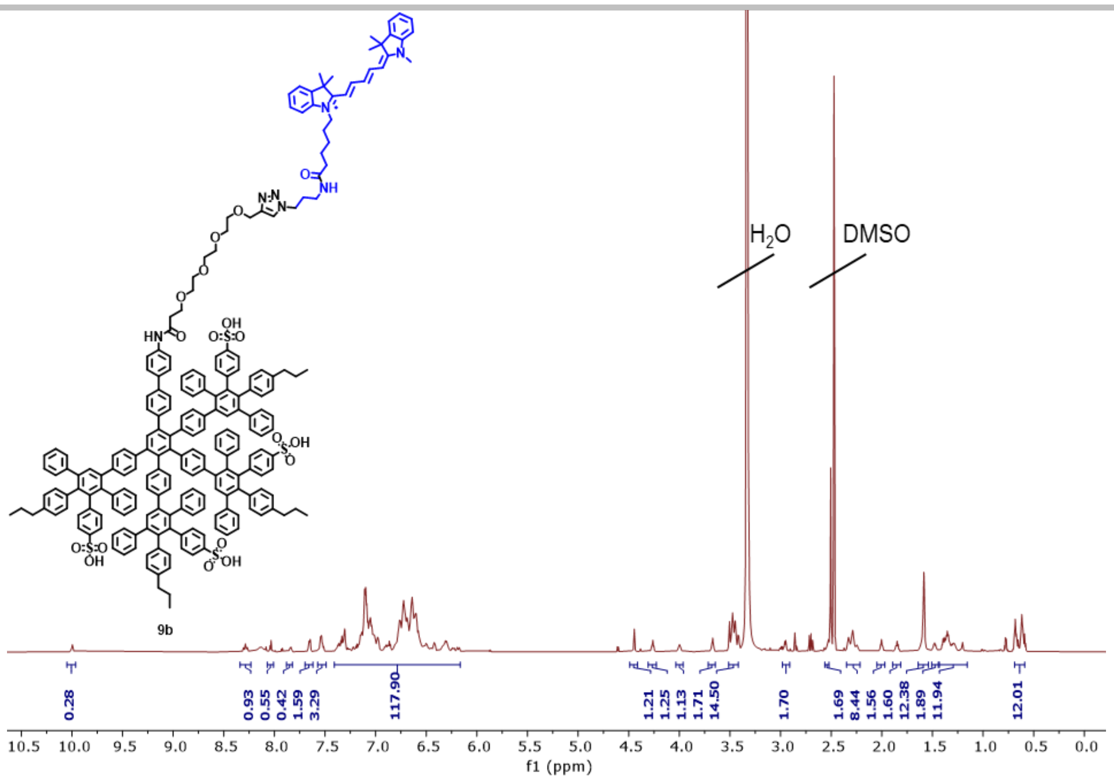

Figure S10. ^{1}H NMR spectrum (700 MHz) of Cy5-dendron **9b** recorded in deuterated dimethyl sulfoxide.

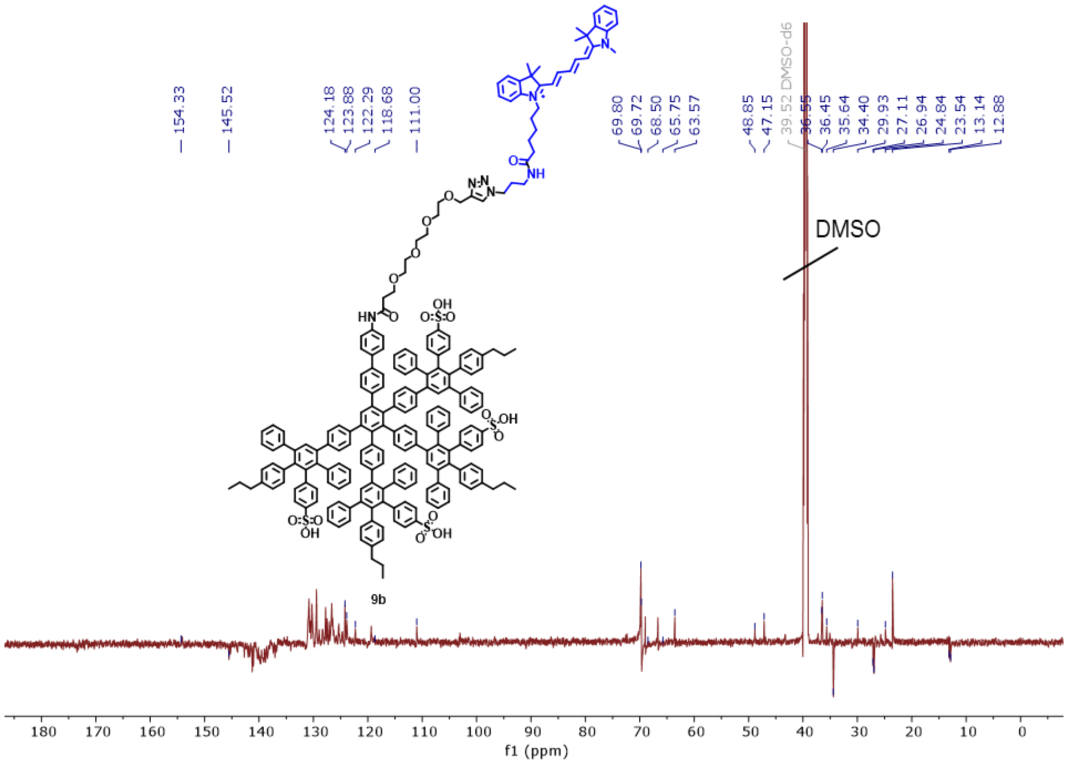

Figure S11. ^{13}C NMR spectrum (176 MHz) of Cy5-dendron **9b** recorded in deuterated dimethyl sulfoxide.

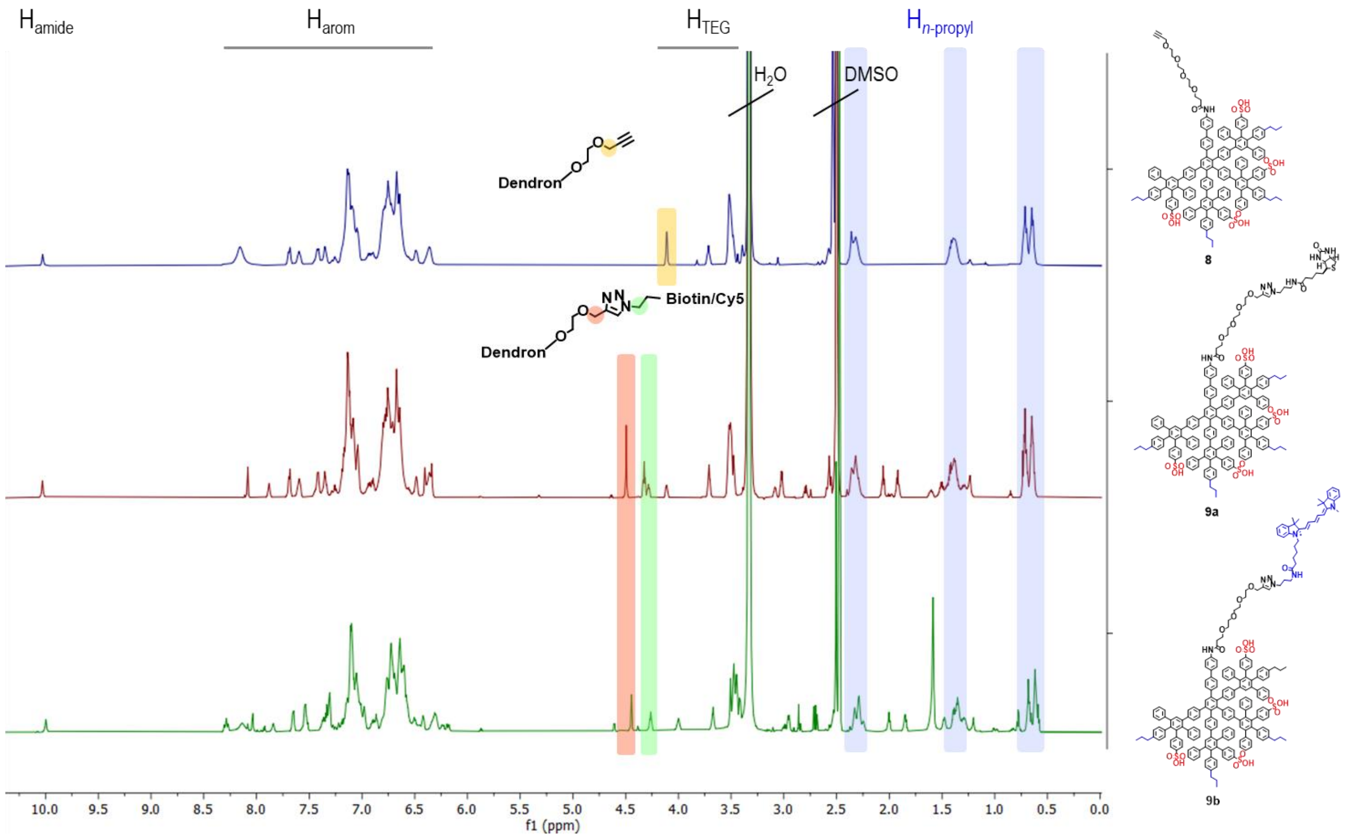

Figure S12. Summarized ^{1}H NMR spectra of final dendrons (**8**, **9a** and **9b**) showing significant shifts of signals (compare yellow and red highlighted signals) and appearance of a characteristic signal (green) after successful CuAAC.

1.7 MALDI-TOF mass spectra of final dendrons

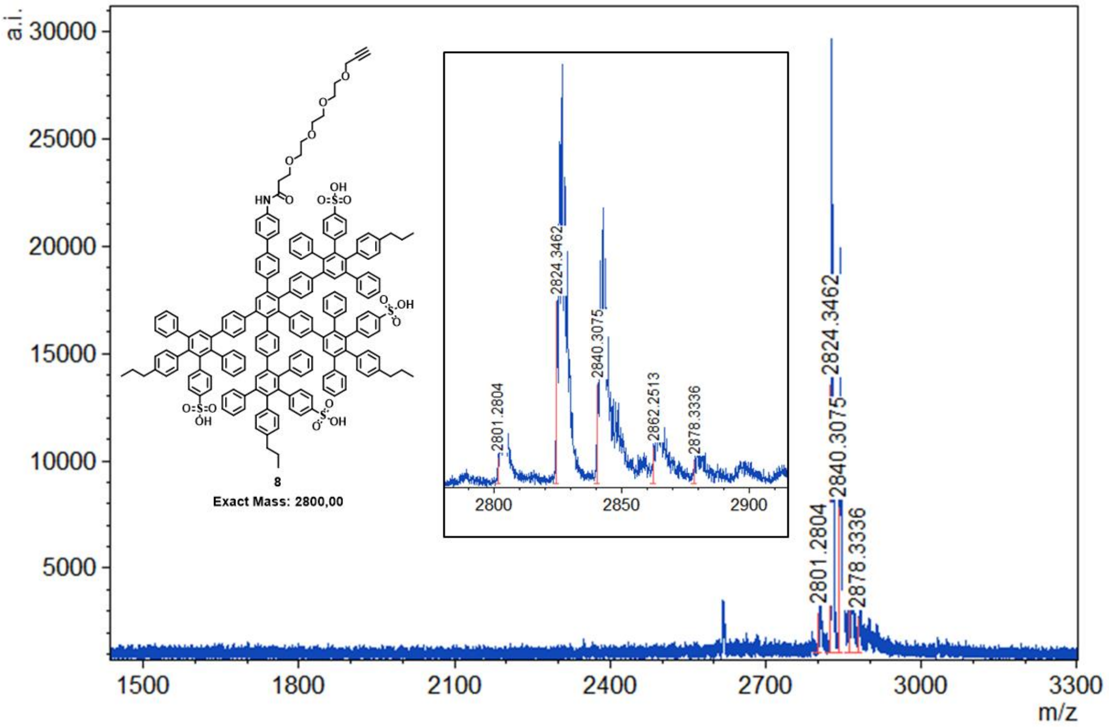

Figure S 13. MALDI-TOF mass spectrum of dendron **8**.

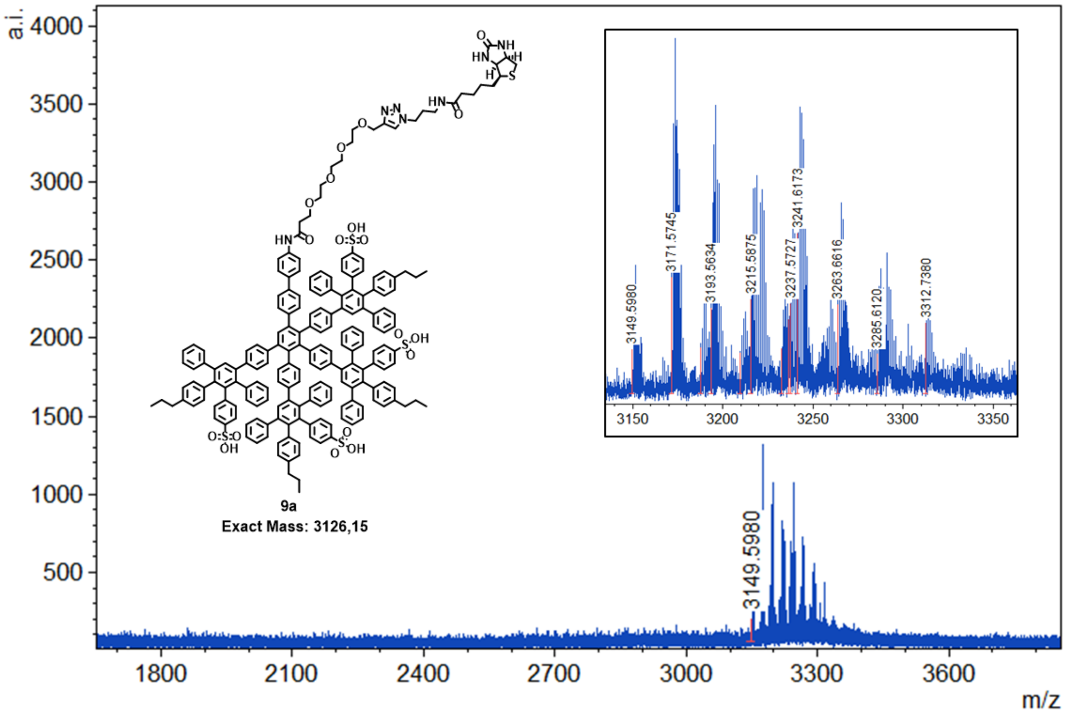

Figure S14. MALDI-TOF mass spectrum of biotin-dendron **9a**

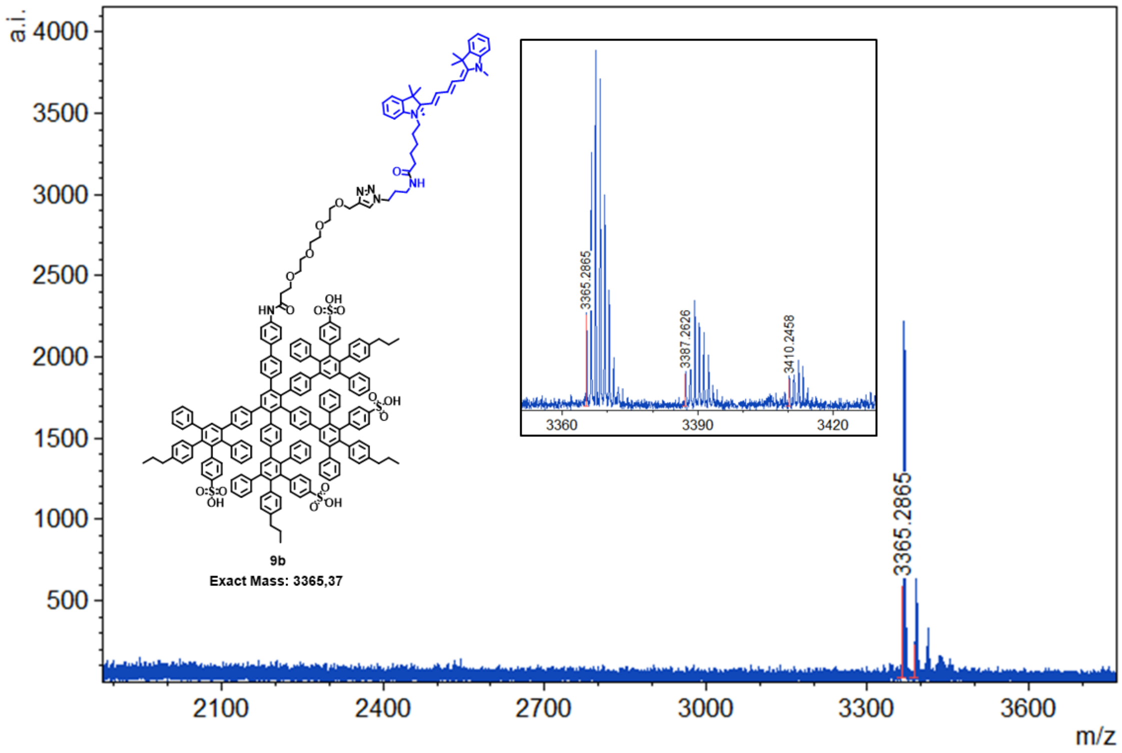

Figure S15. MALDI-TOF mass spectrum of Cy5-dendron **9b**

2 Interaction of dendrons with serum proteins

2.1 Synthesis of liposomes

Amine functionalized liposomes were prepared from 1,2-dioleoyl-*sn*-glycero-3-phosphoethanolamine (DOPE), L-*α*-phosphatidylcholine (egg PC) and cholesterol (Chol) with a molar ratio of egg PC:DOPE:Chol = 1:1:1 by film hydration followed by extrusion. All lipids and cholesterol stock solutions were prepared in chloroform at a concentration of 10 mg mL^{-1}. Afterwards, egg PC (835 µL), DOPE (767 µL) and Chol solutions (398 µL) were added into a 50 mL round-bottomed flask with 2 mL of chloroform containing 1 vol% EtOH. First, the mixture was dried with a rotary evaporator for 30 min at 450 mbar and then for an additional 30 min at 3 mbar and 42 °C. To remove organic solvent residues, the mixture was placed in a vacuum oven for 1 h. (Diameter Ø: 242 ± 6 nm, ζ–Potential: –49 mV ± 7.5 mV)

2.2 Synthesis of polystyrene nanoparticles

Amine functionalized polystyrene nanoparticles were synthesized via the previously reported direct miniemulsion protocol[6]. Cetyl trimethyl ammonium chloride was used a cationic surfactant to stabilize the dispersion and 2-aminoethyl methacrylate hydrochloride (2 wt% to styrene) was copolymerized with styrene. The dispersion was purified via centrifugation and dialysis. A detailed protocol is described in previous reports.[7] (Diameter Ø: 98 ± 10 nm, ζ–Potential: + 49 mV)

2.3 Coating of liposomes and nanoparticles with dendron 8 or dendrimer

Dendron **8** or the amphiphilic dendrimer was dissolved in DMSO at a concentration of 20 mg mL^{-1}. Liposomes-NH_2 (3 mg mL^{-1}, 333 µL) or PS-NH_2 nanoparticles (10 mg mL^{-1}, 100 µL) were incubated with dendron **8** or the dendrimer (20 mg mL^{-1}, 50 µL) for 1 h at room temperature. The mixture was centrifuged (20 000 g, 1 h, 4 °C). Liposomes were resuspended in 100 µL of PBS and polystyrene nanoparticles in 100 µL of water.

Table S1. Zeta Potential measurements of liposomes (20 µL) uncoated or coated with dendron/dendrimer in 1 mM KCl solution (1 mL). Dynamic light scattering measurements at an angle of 90°C of liposomes (20 µL) uncoated or coated with dendron/dendrimer in PBS (1 mL).

	**Zeta Potential (mV)**	**Size (nm)**
**Liposome**	–49 ± 7.5 mV	242 nm ± 6 nm
**Liposome+Dendron**	–32 ± 3.4 mV	863 nm ± 181 nm
**Liposome+Dendrimer**	–27 ± 3.7 mV	963 nm ± 60 nm

2.4 Human plasma/serum

Human blood serum and plasma was obtained from six (serum) or ten (plasma) healthy donors at the Transfusion Center of the University Clinic of Mainz, Germany, pooled and stored at −20 °C. Citrate was used as an anticoagulant for plasma preparation.

2.5 Protein corona preparation

Liposomes coated with dendron **8** and dendrimer as well as nanoparticles (1 mg) were incubated with human serum and plasma (1 mL) for 1 h at 37°C. Subsequently the dispersion was centrifuged (20 000 g, 1 h, 4 °C) and washed with PBS (3 times, 1 mL) to remove loosely and unbound proteins. To desorb the attached corona proteins, the liposome/nanoparticle pellet was resuspended in 100 µL of 2% SDS supplemented with 62.5 mM Tris hydrochloride solution and incubated for 5 min at 95 °C. Afterwards, the dispersion was centrifuged and the supernatant containing the desorbed corona proteins was analyzed by Pierce Assay, SDS PAGE and LC-MS.

2.5.1 Pierce assay

The Pierce 660 nm Protein Assay was used to determine the protein concentration. The assay was performed according to the manufacturer’s instruction. The absorbance was measured with a Tecan infinite plate reader.

2.5.2 SDS PAGE

Proteins (2-3 µg in 26 µL) were loaded on a NuPage 10% Bis-Tris protein gels. Samples were mixed with 4 µL of NuPage Sample Reducing Agent and 10 µL of NuPage LDS Sample Buffer. Electrophoresis was carried out for 1 h at 120 V and gels were stained with Pierce Silver Staining Kit according to the manufacturer’s instruction. All components were obtained from Thermo Fisher.

2.5.3 In solution digestion

Digestion of corona proteins was performed according to former instruction.[8],[9] Briefly, SDS was removed from the protein samples with Pierce detergent removal columns (Thermo Fisher). Afterwards, the proteins were precipitated overnight using ProteoExtract protein precipitation kit (CalBioChem) according to the manufacturer's instructions. The resulting proteins pellet was re-suspended in RapiGest SF (Waters Cooperation) dissolved in ammonium bicarbonate (50 mM) buffer. Proteins were reduced with dithiothreitol (Sigma, 5 mM, 45 min at 56 °C) and alkylated with idoacetoamide (Sigma, 15 mM, 60 min at room temperature). A ratio between protein:trypsin (50:1) was used and the digestion was carried out over 16 h at 37 °C. The reaction was quenched with 2 µL hydrochloric acid (Sigma).

2.5.4 Liquid chromatography coupled to mass spectrometry (LC-MS analysis)

Peptide samples were diluted with 0.1% formic acid and 50 fmol μL^{-1} Hi3 Ecoli (Waters Cooperation) was added for absolute protein quantification.[10] LC-MS measurements were performed with a Synapt G2- Si mass spectrometer coupled to a nanoACQUITY UPLC. A NanoLockSpray source was used in positive ion mode for electrospray ionization (ESI). Data-independent acquisition (MS^E) experiments were carried out and the Synapt G2-Si was operated in resolution mode. For data acquisition and processing MassLynx 4.1 and peptides/proteins were identified with Progenesis QI (2.0). The human database was downloaded from Uniprot modified with the sequence information of Hi3 Ecoli standard for absolute quantification. Processing parameters for peptide and protein identification were applied as described in detail in previous reports.[11] The absolute amount of each protein was determined in fmol based on the TOP3/Hi3.[12] Each measurement was performed in technical duplicates or triplicates.

2.6 Protein Corona: Supplementary figures

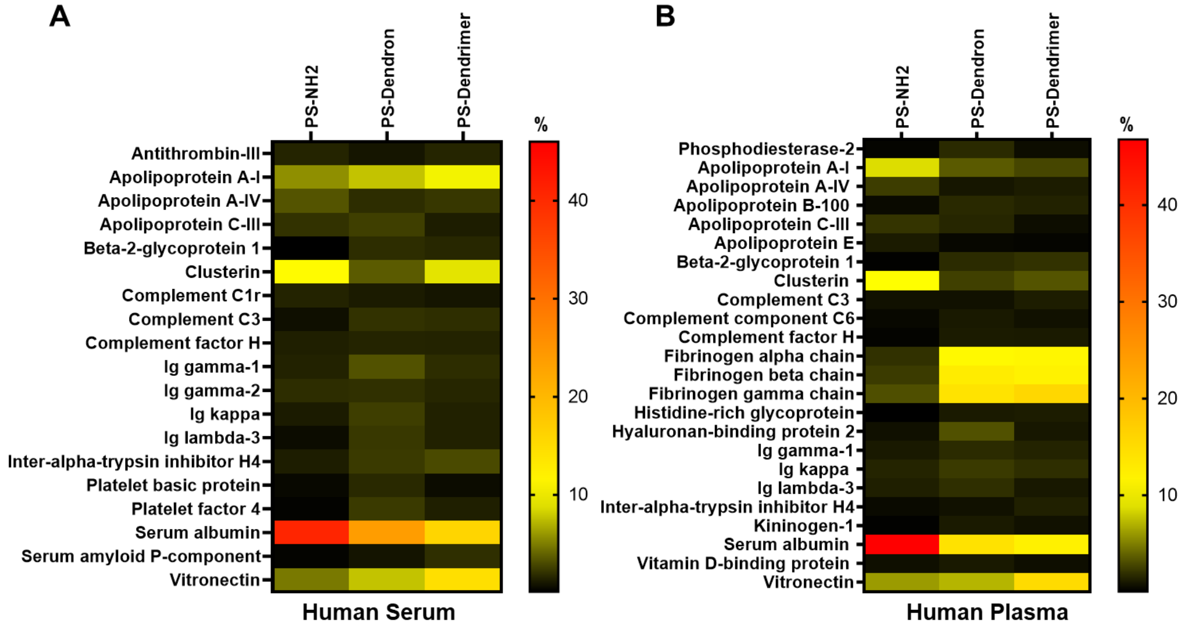

Figure S16. Heat map of adsorbed proteins to dendron **8** and dendrimer coated polystyrene nanoparticles in (**A**) blood serum and (**B**) blood plasma. The amount of each protein is given in % based on all identified corona proteins. A list of all identified proteins is supplemented at the end of the SI (Fig. S34-S37).

Table S2. Average amount in % and the standard deviation of identified proteins adsorbed to lipo-dendron and lipo-dendrimer mentioned in the main manuscript. A list of all identified proteins can be found at the end of this document (Fig. S34-S37).

	Liposome	Liposome + Dendron	Liposome + Dendrimer
**Vitronectin**	1 ± 0.7% serum	9 ± 0.8% serum	6 ± 0.5% serum
	0.2 ± 0.3% plasma	6 ± 0.5% plasma	3 ± 0.2% plasma
**ApoH**	0.5 ± 0.2% serum	8 ± 0.5% serum	5 ± 0.3% serum
	0.3 ± 0.5% plasma	11 ± 1.5% plasma	4 ± 0.3% plasma
**IgG kappa**	6 ± 1% serum	2 ± 0.2% serum	2 ± 0.1% serum

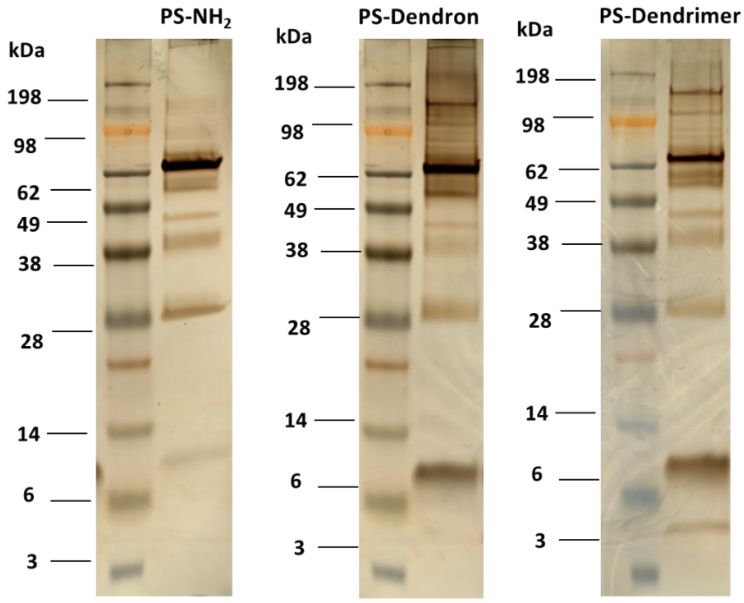

Figure S17. Hard protein corona analysis of polystyrene nanoparticles ($PS-NH_2$) coated with dendron **8** or dendrimer PPD3 after serum incubation. 2–3 µg of protein was applied to the SDS-PAGE (reducing conditions).

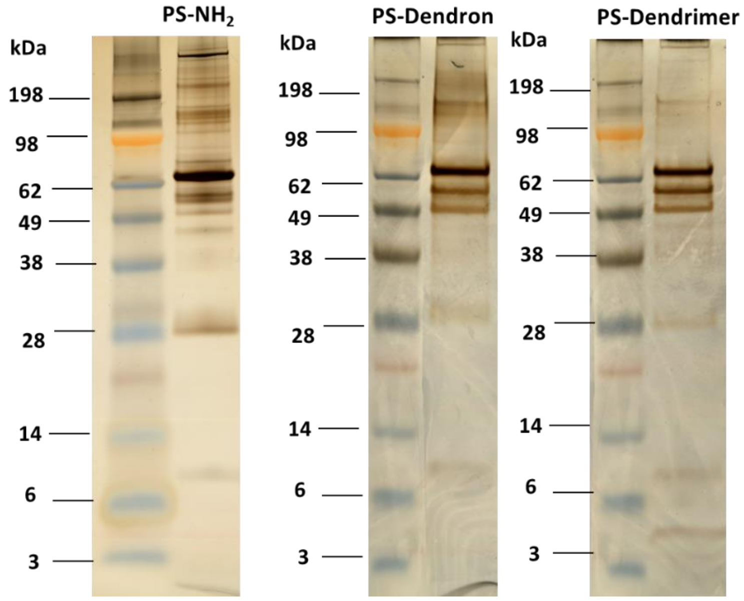

Figure S18. Hard protein corona analysis of polystyrene nanoparticles ($PS-NH_2$) coated with dendron **8** or dendrimer PPD3 after plasma incubation. 2–3 µg of protein was applied to the SDS-PAGE (reducing conditions).

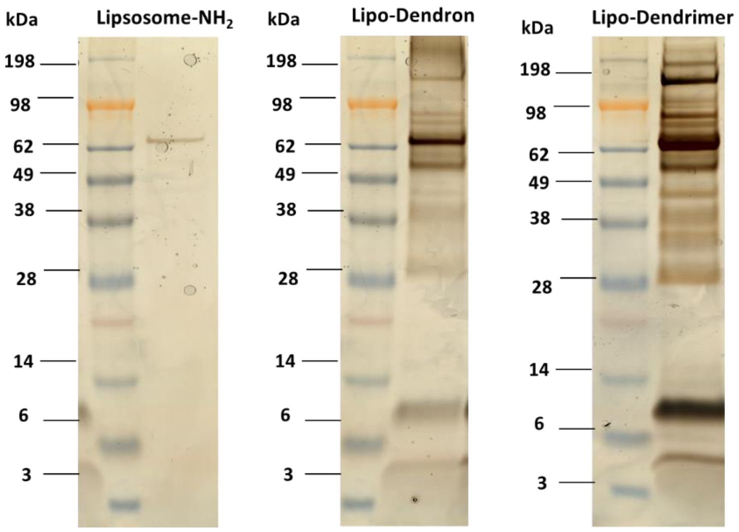

Figure S19. Hard protein corona analysis of liposomes (Lipo-NH_2) coated with dendron **8** or dendrimer PPD3 after serum incubation. 2–3 μg of protein was applied to the SDS-PAGE (reducing conditions).

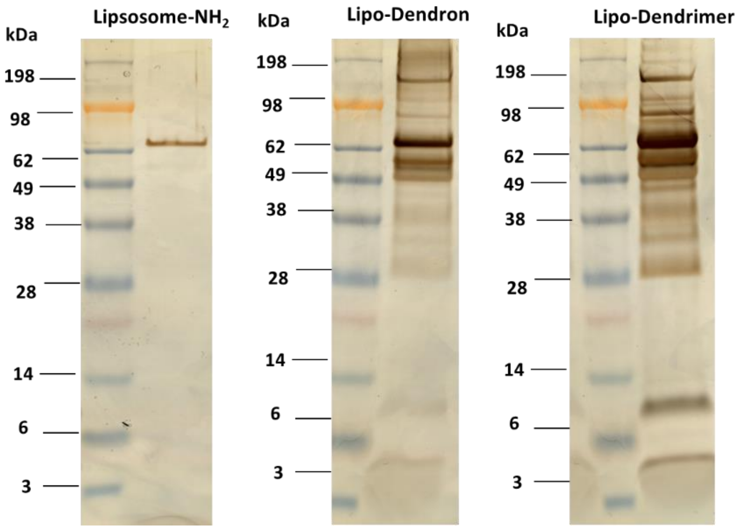

Figure S20. Hard protein corona analysis of liposomes (Lipo-NH_2) coated with dendron **8** or and dendrimer after plasma incubation. 2–3 μg of protein was applied to the SDS-PAGE (reducing conditions).

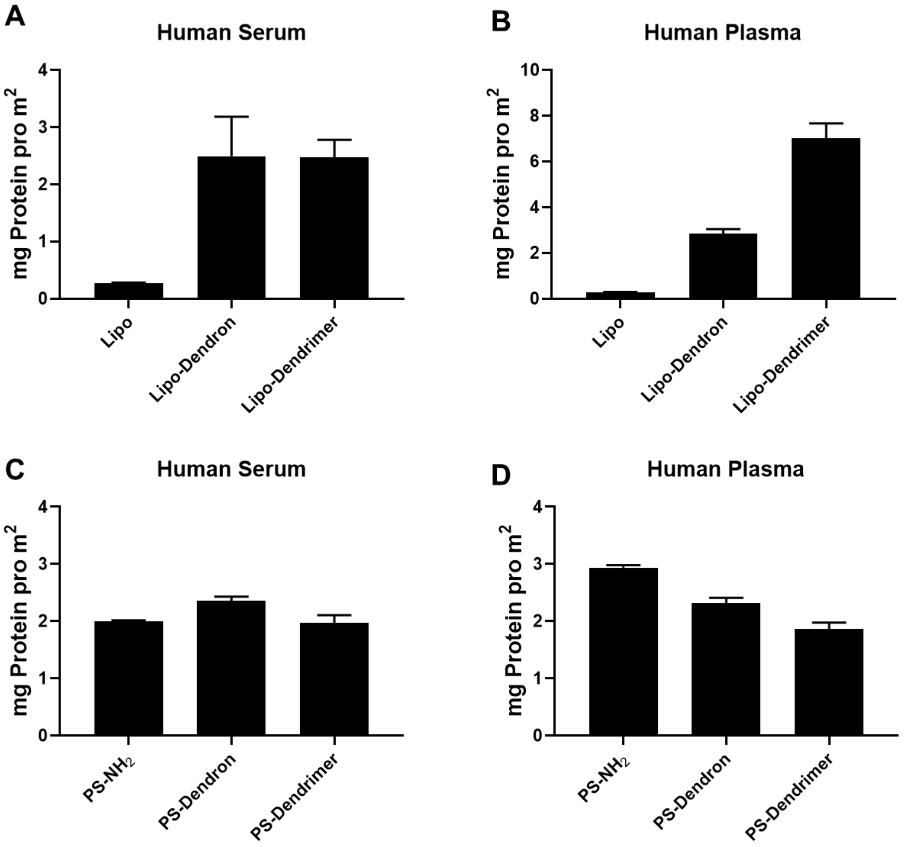

Figure S21. The absolute amount of adsorbed corona proteins (in mg) per surface area (m^2) of the liposomes (A,B) and polystyrene nanoparticles (C,D) was determined via Pierce Assay.

2.7 Interaction of Amphiphilic PPDs with lipid monolayer

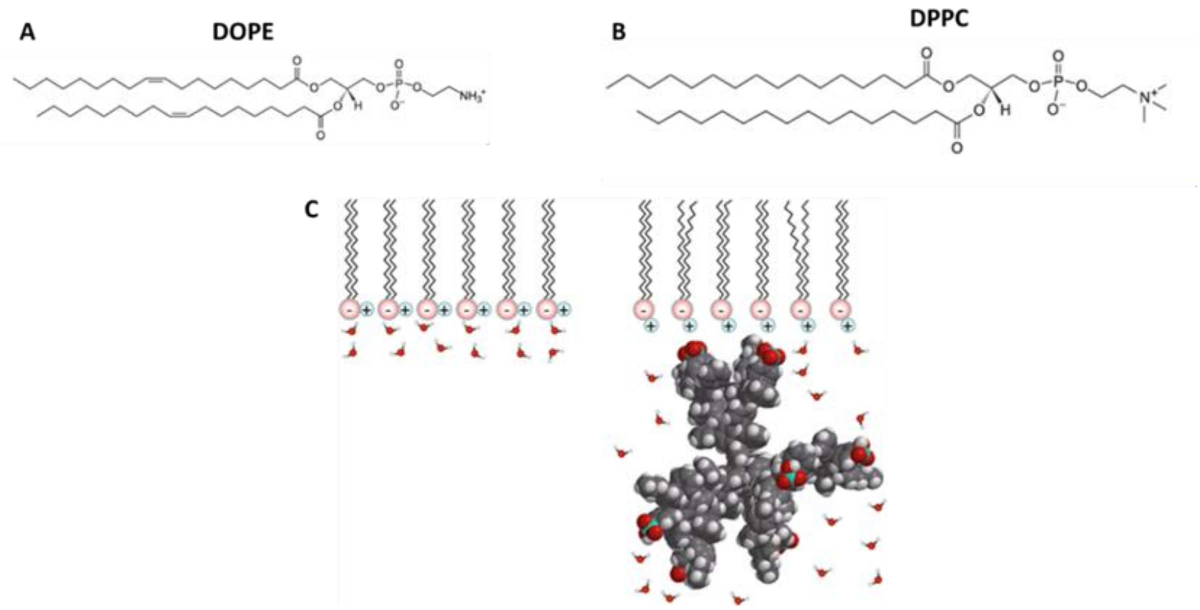

Figure S22. Assumption how the amphiphilic PPD surface motif might interact with the liposomes. (**A**), (**B**) Molecular structure of the lipids DOPE and DPPC. (**C**) Illustration of the molecular configuration of DPPC, water, and the dendrimer molecules before (left) and after (right) dendrimer injection (Figure adapted with permission from Langmuir **2015**, 31, 1980-1987; https://pubs.acs.org/doi/10.1021/la504252s; © 2015 American Chemical Society). The interactions between an amphiphilic PPD and a lipid monolayer consisting of zwitterionic lipids (DPPC, (**B**)) were studied. By X-ray reflectivity measurements it was found that the interaction between the amphiphilic dendrimers and the zwitterionic lipids is mainly electrostatic. They proved that upon adsorption of the dendrimer towards the lipid surface, the monolayer remains intact.[13] As DOPE is also a zwitterionic lipid with a similar structure, we assume that the interaction between the amphiphilic dendron or dendrimer and the DOPE liposomes are also electrostatically driven. An incorporation of the lipophilic n-propyl group was not observed.

3 Cellular uptake and cytotoxicity studies

3.1 Materials and Instruments

Confocal laser scanning microscopy was performed using Leica TCS SP5. CellTiter-Glo® Cell Viability Assay was purchased from Promega and luminescence intensities were measured on a Glomax Multi 96-well plate reader from Promega.

3.2 Methods

3.2.1 Cell culture

CHO-K1 (Chinese hamster ovary cell line) cells were obtained from DSMZ-German Collection of Microorganisms and Cell Cultures GmbH and cultured in DMEM/F12 medium (Gibco®) supplemented with 10% fetal bovine serum (FBS), 1% penicillin/streptomycin and 1x MEM (Minimum Essential Medium) non-essential amino acids at 37 °C in a humidified 5% CO_2-Incubator.

3.2.2 Cellular uptake of Cy5-dendron 9b

CHO-K1 cells at a density of 15 000 cells/well were seeded in a 8 well chambered µ-Slide coverslip (ibidi GmbH, Germany) in 200 µL medium and were incubated for 24 h to allow adhesion. Then, Cy5-dendron **9b** was predissolved in DMSO and diluted in ultrapure water to achieve a final concentration of 3 mg mL^{-1} (0.1% DMSO). The Cy5-dendron stock solution was diluted in medium to obtain a concentration of 1 µM.

200 µL of dendron solution were added to the well. As blank control, cells were incubated with fresh media. After incubation for 24 h at 37 °C and 5% CO_2 the medium containing the dendron was removed. Cell nuclei were stained with Hoechst 33258 for 15 min in medium and cells were washed for three times with PBS. After adding of fresh medium cells were imaged using a confocal laser scanning microscope (TCS SP5) equipped with a 63x oil immersion objective. The emission of Cy5 labelled dendron **9b** was recorded using a 633 HeNe laser for excitation with a detection bandwidth of 645-745 nm and Hoechst nucleus staining was recorded using a 405 Diode with a detection bandwidth of 415-500 nm. Acquired images were processed with ImageJ.

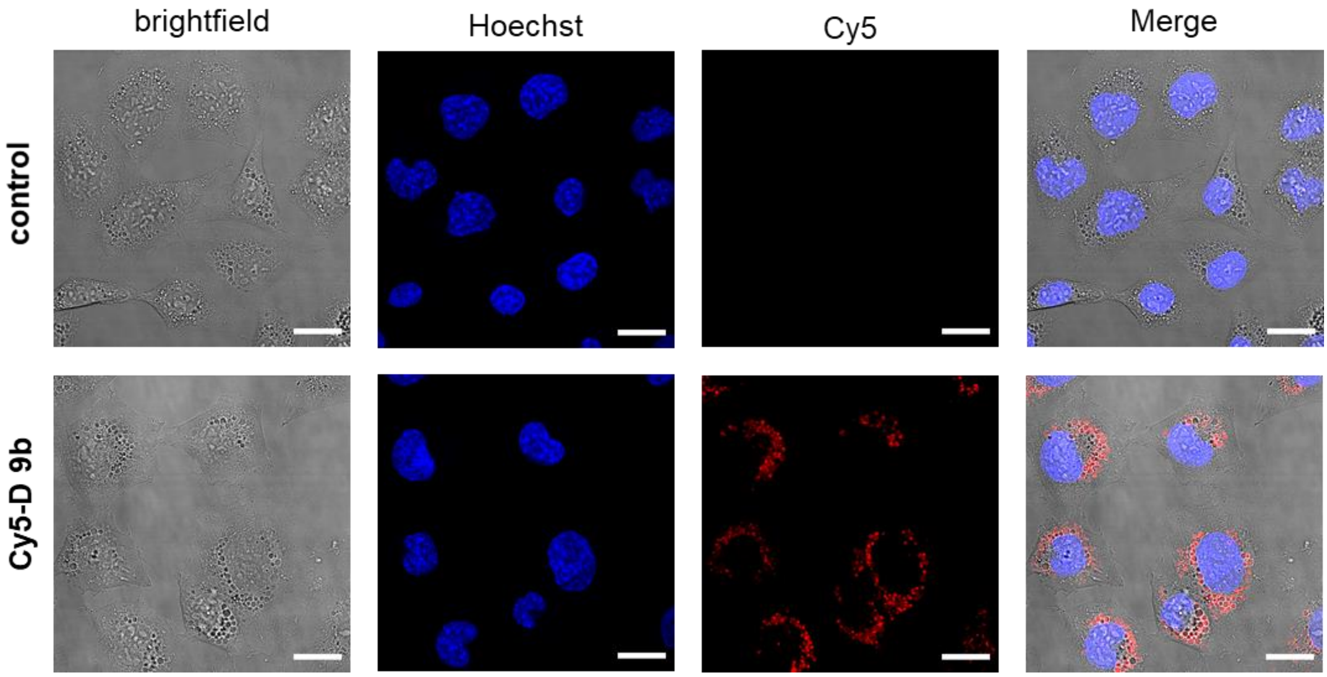

Figure S23. Cellular uptake of Cy5-dendron **9b** in CHO-K1 cells. Cells were treated with 1 µM dendron solution in medium for 24 h. As blank control cells were incubated with fresh medium for 24 h. After staining the nuclei with Hoechst 33258, cells were imaged by confocal laser scanning microscopy (scale bar = 20 µm).

3.2.3 Cell viability/cytotoxicity–CellTiter-Glo® luminescent cell viability assay

CHO-K1 cells at a density of 6000 cells/well were seeded in a white 96-well plate and incubated for 24 h to allow attachment. Propargyl-dendron **8**, biotin-dendron **9** as well as PPD3 were predissolved in DMSO at concentrations of 20 mg mL^{-1} and diluted in ultrapure water to achieve stock solutions with a final concentration of 1 mM for dendron conjugates **8** and **9a** as well as 100 µM for PPD3. The medium was removed, various concentrations (1-40 µM) dendron-conjugates **8** and **9a** as well as PPD3 in medium were added and the cells were incubated for further 24 h. As blank control cells were incubated with fresh media. After incubation, cell viability of CHO-K1 cells treated with dendron conjugates was determined applying CellTiter-Glo®-Assay from Promega (G7570) according to manufacturer's instructions.

4 Adenovirus 5 studies

4.1 Materials

Human adenovirus type 5 (Ad5, pAV[Exp]-CMV>EGFP) was purchased from Hanbio (China) and Cyagen Biosciences (China). CHO-K1 cell line was purchased from China Center for Type Culture Collection (Wuhan University). DME/F12 medium, fetal bovine serum (FBS) and phosphate buffered saline (PBS) were purchased from Hyclone (USA). Penicillin/streptomycin, (4-{[bis-(1-*tert*-butyl-1H-[1,2,3]triazol-4-ylmethyl)-amino]-methyl}-[1,2,3]triazol-1-yl)-acetic acid (BTTAA), $CuSO_4$ and sodium ascorbate were purchased from Sigma Aldrich (USA).

4.2 Instruments

Fluorescent imaging was carried out by a fluorescent microscope MF52 (Guangzhou Micro-shot Technology Co., Ltd., China). Transmission electron microscopy (TEM) was carried out by HT7700 (Hitachi, Japan). Flow cytometry was performed with FC500 (Beckman, USA). Fluorescence of coumarin was measured by microplate reader Varioskan LUX (Thermo Fisher Scientific, USA). Fluorescence spectra and intensities were measured on a SPARK 20M microplate reader from TECAN Group Ltd or Varioskan LUX (Thermo Fisher Scientific, USA). Dynamic light scattering and Zeta potential were measured by Zetasizer nano ZS90 (Malvern, UK).

4.3 Methods

4.3.1 Complex formation and characterization of dendron/Ad5 interaction

Complex formation of Ad5 and dendron conjugates or dendrimer PPD3 was performed in 5 µL of phosphate buffered saline (PBS). This volume contained 4×10^8 Ad5 virus particles (VP) (the concentration of Ad5 was quantified by measuring the absorbance at 260 nm and converted to number of particles with the equation: concentration (VP/mL) = $OD_{260} \times 1.1 \times 10^{12}$). Dendron-conjugates were dissolved in DMSO to obtain a stock solution of 1 mM and further diluted in 5 mM phosphate buffer, pH 7.4 to achieve a concentration of 50 µM. Dendron or dendrimer was added in defined ratios (for TEM, ratio of Ad5 to dendron is 1:100,000). After incubation of Ad5 and dendron for 40 min, 5 µL of Ad5 or Ad5/dendron was added on a copper grid for 5 min, followed by staining with uranyl acetate (3%) for 45 s. Copper grid was blot dry by filter paper and dried for 2 h before imaging in TEM.

This is the calculation for the Dendron/Ad5 ratio of 1:100,000 (N, number of particles; r, ratio between Dendron and Ad5; V, volume; N_A , Avogadro constant; c, concentration):

$$V = \frac{n}{c} = \frac{N/N_A}{c} = \frac{N \times r/N_A}{c} = \frac{4 \times 10^8 VP \times 1 \times 10^5/(6.02 \times 10^{23} mol^{-1})}{50 \times 10^{-6} mol/L} = 1.33 \times 10^{-6} L$$

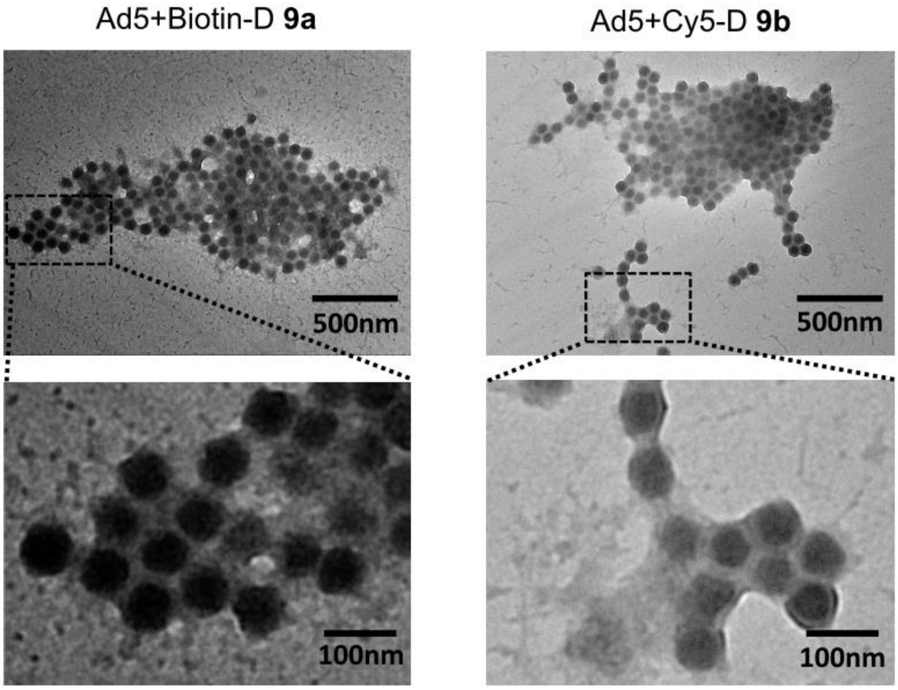

Figure S24. TEM images show binding of dendron-conjugates (Biotin-D **9a** and Cy5-D **9b**) to Ad5.

4.3.2 Dynamic light scattering (DLS) and zeta potential

4.3.2.1 DLS and zeta potential at diluted conditions

Dynamic light scattering was used to determine interaction between Ad5 and dendrons by means of measuring the polydispersity index (PDI) and the hydrodynamic diameter of the particles. Complex formation was performed in a volume of 30 µL phosphate buffer (5mM, pH7.4) with 5×10^8 Ad5 particles. Dendron was added in defined ratios to Ad5, then mixed and incubated for 40 min. After transfer to a cuvette, it was filled up with PB to a total volume of 0.9 mL. All samples were measured at 25 °C and

an angle θ = 90°. For intensive cleaning of the cuvette, ethanol and acetone was used to avoid measurement errors by dust particles.

Zeta potential was used to determine the charge on the surface of Ad5 or complexes of Ad5 and dendrons. All samples were prepared the same as DLS and measured at 25 °C.

Table S3. Size and zeta potential of Ad5/dendron complexes with an Ad5-concentration of 4.5 x 10^8 vp/mL in 5 mM phosphate buffer after incubation for 40min.

Sample	Size(nm)	PDI	Zeta potential (mV)
Only Ad5	109.4 ± 1.0	0.149	–18.1 ± 2.0
Ad5+Propargyl-D (1:1k)	113.8 ± 3.6	0.103	–19.6 ± 1.4
Ad5+Propargyl-D (1:20k)	159.2 ± 11.7	0.186	–29.0 ± 1.0
Ad5+Propargyl-D (1:100k)	684.5 ± 25.8	0.610	–37.5 ± 0.9
Ad5+Biotin-D (1:100k)	639.0 ± 31.3	0.608	–30.3 ± 1.6

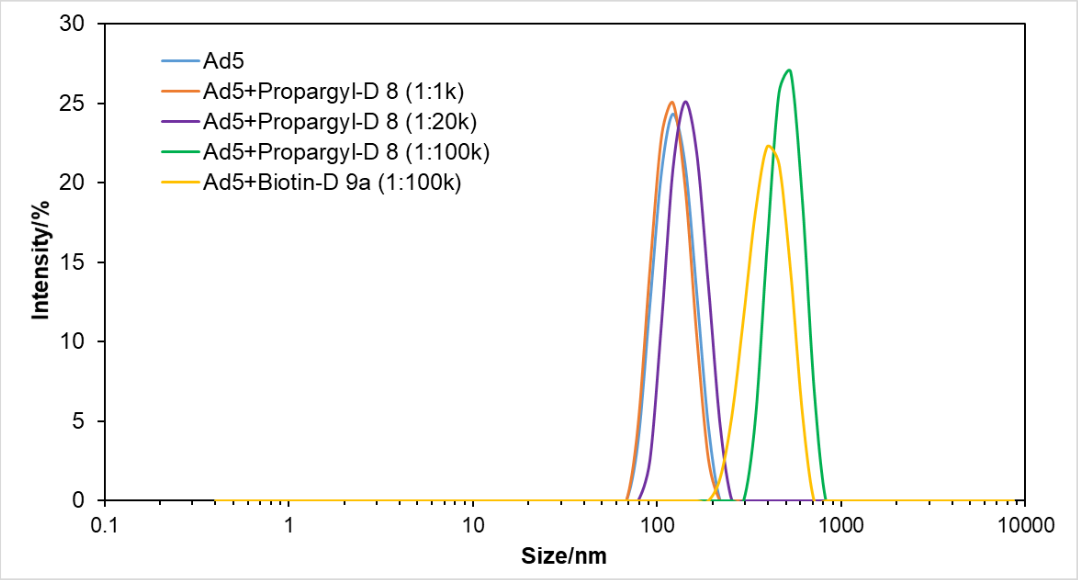

Figure S25. Size distribution by intensity. Ad5 vector with a concentration of 4.5x10^8 vp/mL in 5 mM phosphate buffer, pH 7.4 was incubated for 40 min with propargyl-dendron **8** at the ratios 1:1k–1:100k (Ad5:Dendron) as well as biotin-dendron **9a** at the ratio of 1:100k.

4.3.2.2 DLS and zeta potential at high Ad5 concentration and high ratios

The surface charge of vector particles was measured using a ZetaSizer Nano-ZS (Malvern, Worcestershire, UK) and analyzed with ZetaSizer 7.12 software. For the analysis an *E1*-deleted replication-incompetent human adenovirus type 5 vector (GenBank ID: AY339865.1, sequence from nt 1 to 440 and from nt 3523 to 35935) was used. The vector carried a CMV promoter-driven enhanced GFP expression cassette, subcloned from a pEGFP-N1 plasmid (Clontech 6085-1) that was inserted in reverse orientation in the deleted E1 region.

Dendron-conjugates (propargyl-dendron **8** and biotin-dendron **9a**) were dissolved in DMSO to achieve 20 mg mL^{-1} stock solutions. Then, 1×10^{11} Ad5 particles were dispersed in 1 mL 50 mM HEPES buffer, pH 7.4 and dendron was added at the ratio 1:200k (Ad5: Dendron) and 1:1000k respectively according to the calculation described in chapter 4.3.1. In order to determine the saturation of the Ad5 vector by dendrons DLS and zeta potential of propargyl-dendron **8** and biotin-dendron **9a** without Ad5 were measured as negative controls. A higher Ad5 concentration compared to the DLS measurements in 4.3.2.1 was applied to compare the size of Ad5/dendron complexes with the size of free dendron (free dendron is smaller and cannot be detected at lower concentrations). In addition the saturation of Ad5 at a certain ratio was studied.

Table S4. Size and zeta potential of Ad5/dendron complexes. Ad5 vector with a concentration of $1x10^{11}$ vp/mL was incubated with propargyl-dendron **8** at the ratios 1:200k (Ad5:Dendron) and 1:1000k as well as biotin-dendron **9a** at the ratio 1:200k in 50 mM HEPES buffer pH 7.4 for 15 min. We observed an increase in size when mixing dendron with Ad5 and for Ad5 + propargyl-dendron **8** at the ratio of 1:1000k and biotin-dendron **9a** at a ratio 1:200k a second peak was observed (Fig. S26).

Sample	Size (nm)			PDI	Zeta potential (mV)
	Z-average	Intensity Peak 1	Intensity Peak 2		
Only Ad5	116.6 ± 0.5	120.6 ± 0.5	-	0.01	–18.3 ± 1.5
Ad5+Propargyl-D **8** (1:200k)	202.4 ± 2.7	263.6 ± 4.5	-	0.23	–41.2 ± 1.9
Ad5+Propargyl-D **8** (1:1000k)	179.2 ± 1.2	473.9 ± 13.8	61.5 ± 1.78	0.48	–43.8 ± 2.8
Ad5+Biotin-D **9a** (1:200k)	241.6 ± 2.7	500.3 ± 40.5	80.7 ± 5.3	0.48	–38.2 ± 2.2

Table S5. Size and zeta potential of free dendrons as control. Propargyl-dendron **8** and biotin-dendron **9a** were incubated in 50 mM HEPES buffer pH 7.4 for 15 min (200k = 33.4 µM, 1000k = 167 µM). .

Sample	Size (nm)			PDI	Zeta potential (mV)
	Z-average	Intensity Peak 1	Intensity Peak 2		
Propargyl-D **8** (200k)	76.0 ± 1.9	111 ± 4.4	-	0.31	−22.6 ± 3.0
Propargyl-D **8** (1000k)	88.4 ± 1.5	127 ± 1.0	-	0.32	−39.7 ± 3.0
Biotin-D **9a** (200k)	64.9 ± 1.4	81.0 ± 2.4	-	0.28	−28.2 ± 2.6

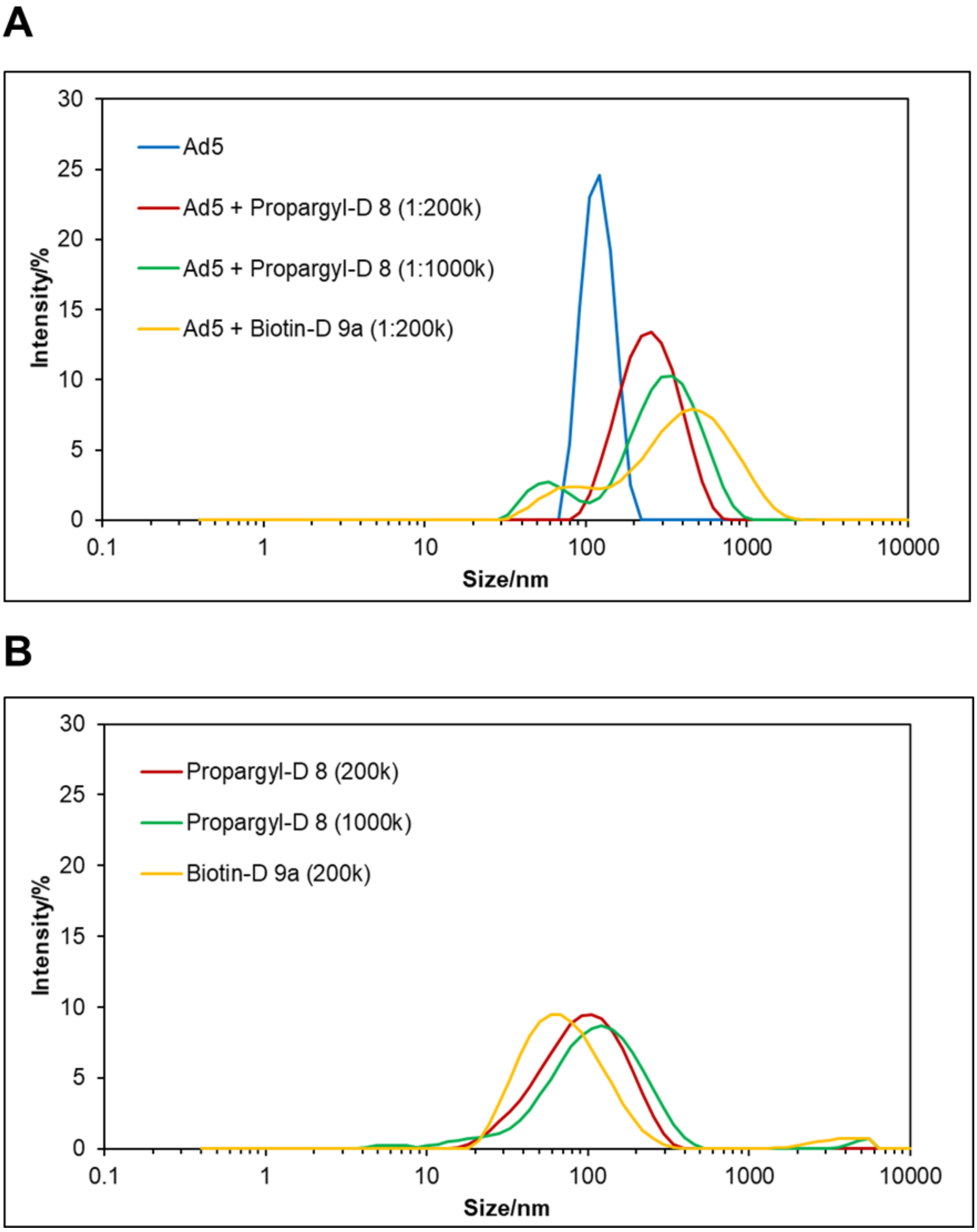

Figure S26. Size distribution by intensity. (**A**) Ad5 vector with a concentration of 1x10^{11} vp/mL was incubated with propargyl-dendron **8** at the ratios 1:200k (Ad5:Dendron) and 1:1000k as well as biotin-dendron **9a** at the ratio 1:200k in 50 mM HEPES buffer pH 7.4 for 15 min. Then, DLS was measured and the saturation of Ad5 by the dendrons was verified. We observed an increase in size when mixing dendron with Ad5 and for Ad5 + propargyl-dendron **8** at the ratio of 1:1000k and biotin-dendron **9a** at a ratio 1:200k a second peak was observed. To assess whether this peak is related to unbound dendron in the mixture, free dendron was measured at same concentrations, which is shown in (**B**). For free dendron-conjugates we observed a size of about 100 nm which can be explained by assembly processes of the dendron in buffer solution due to its amphiphilic nature. Thus, we assume that the second peak (for Ad5 + propargyl-dendron **8** at the ratio of 1:1000k and biotin-dendron **9a** at a ratio 1:200k) is related to unbound dendron which means that the Ad5 vector is saturated at these ratios.

4.3.3 Transduction in CAR-negative cell line CHO-K1

For transduction assays, 24-well plates were used containing 5 × 10^4 cells per well, which were seeded the day before transduction. Cells were cultured in DMEM/F12 medium with 10% FBS and 1% penicillin/streptomycin (PS, 10 000 IU penicillin and 10 000 µg/mL streptomycin) at 37 ºC in a humidified atmosphere containing 5% CO_2. Dendron conjugates were added to Ad5 in defined ratios (1:20k (1:20 × 10^3), 1:50k, 1:100k, 1:200k, 1:500k) and then incubated for 40 min. Cells were infected by Ad5 or Ad5/dendron with pMOI (particle multiplicity of infection) 200 unless otherwise specified and incubated for 4 h at 37 °C. Then, Ad5 or Ad5/dendron was removed, and cells were continued to culture for 24 h. EGFP positive cells and the overall mean fluorescence intensity (MFI) of EGFP expression was measured by fluorescent microscope (EGFP, excitation: 488nm, emission: 510nm) and flow cytometry (For EGFP, excitation: 488 nm, emission: 510 nm; for Cy5: excitation: 630 nm, emission: 670 nm).

This is a calculation example for a Dendron/Ad5 ratio of 1:500,000 (N, number of particles; N_{cell}, number of cells; pMOI, particle multiplicity of infection; r, ratio between Dendron and Ad5; V, volume; N_A , Avogadro constant; c, concentration):

$$V = \frac{n}{c} = \frac{N/N_A}{c} = \frac{N_{cell} \times pMOI \times r/N_A}{c} = \frac{5 \times 10^4 \times 200\ VP \times 5 \times 10^5/(6.02 \times 10^{23} mol^{-1})}{5 \times 10^{-6} mol/L}$$
$$= 1.66 \times 10^{-6} L$$

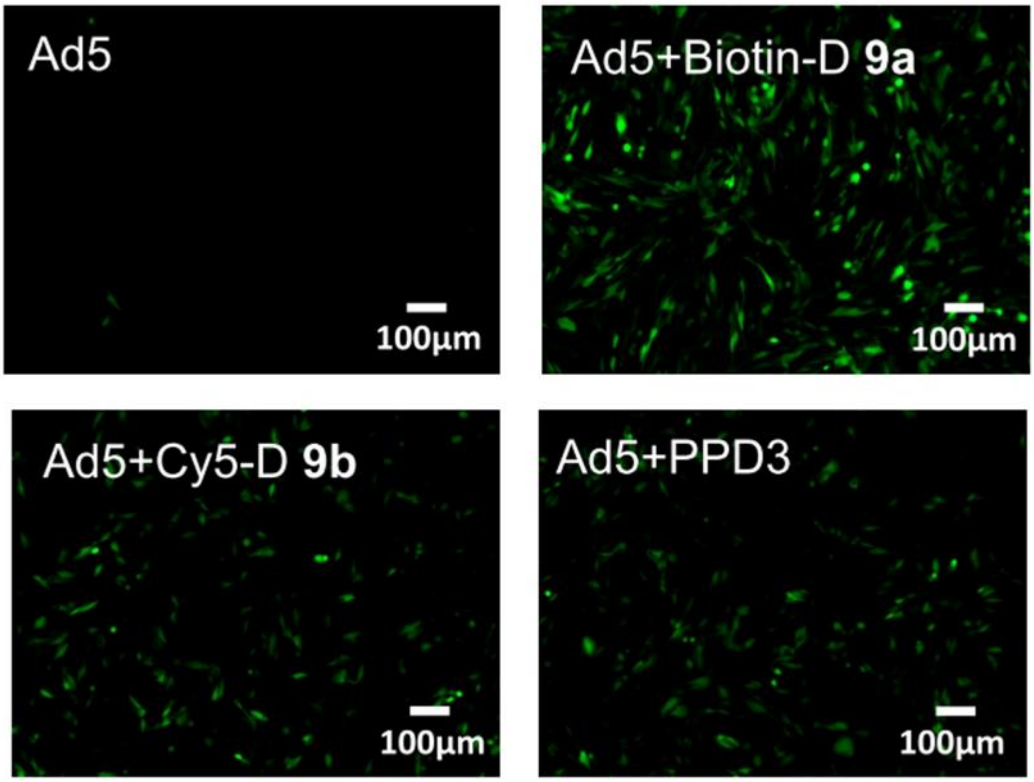

Figure S27. Fluorescent microscopy image of EGFP-transduction in CAR-negative CHO-K1 cells with infection by Ad5 (control), Ad5/dendron (Ad5: dendron=1: 500k) or Ad5/dendrimer (Ad5: dendrimer=1: 125k).

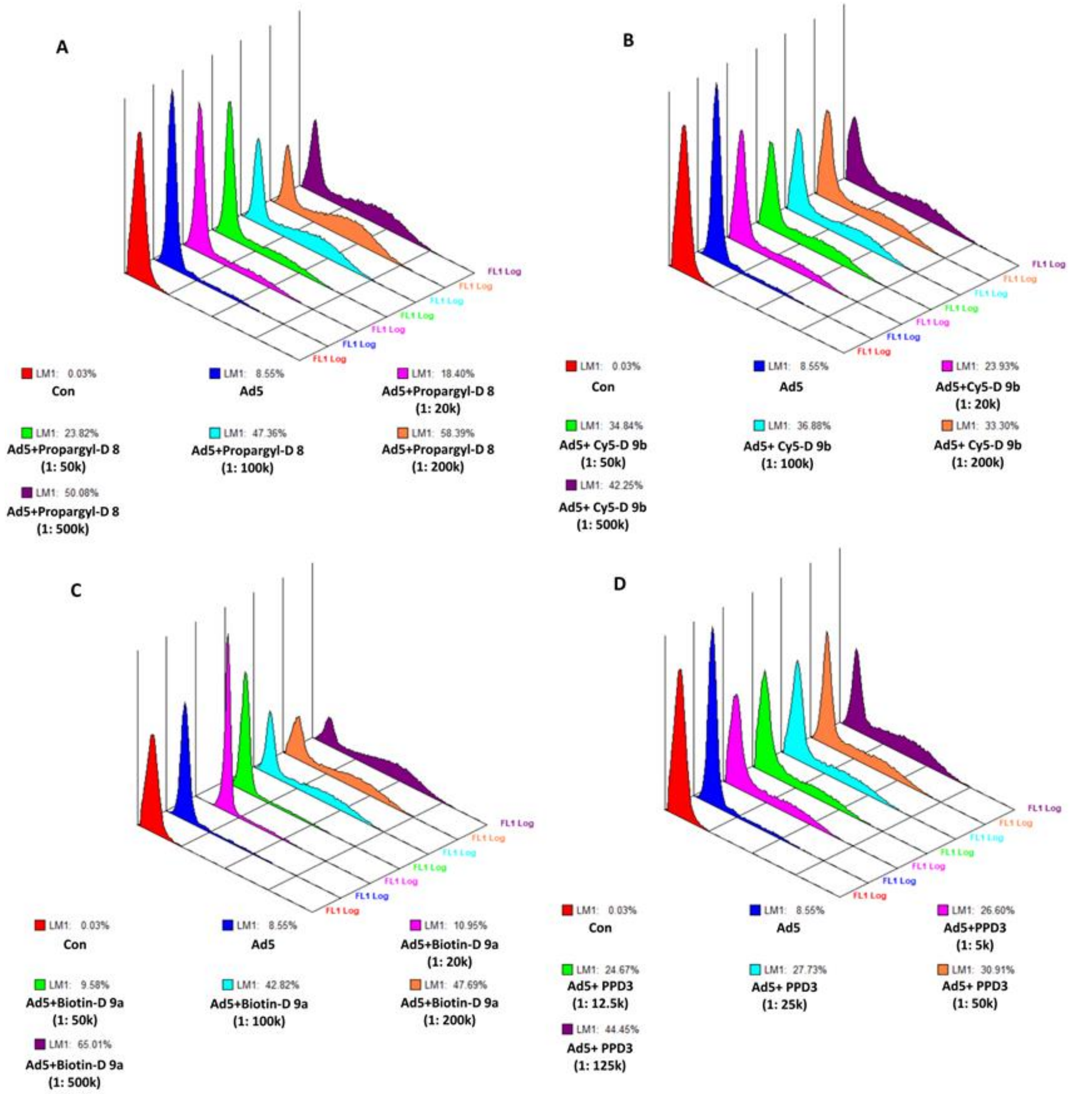

Figure S28. Flow cytometric quantification for CHO-K1 infected by Ad5 or Ad5/dendron. (**A**), (**B**), (**C**), (**D**) represent the results of Ad5/propargyl-D **8**, Ad5/Cy5-D **9b**, Ad5/biotin-D **9a** and Ad5/PPD3, respectively.

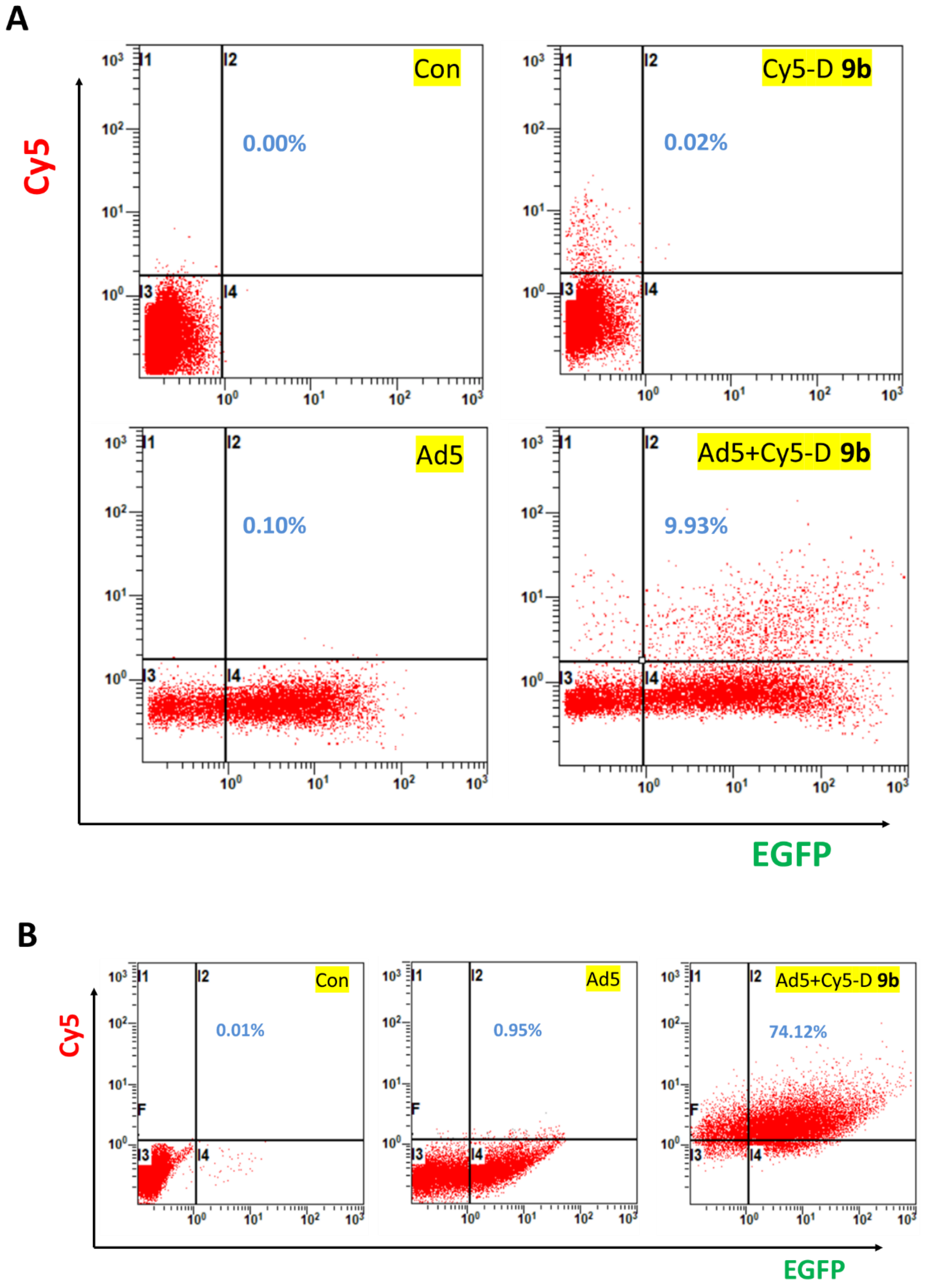

Figure S29. Flow cytometric quantification for CHO-K1 infected by Ad5 or Ad5/Cy5-D **9b**. (**A**) The pMOI is 500 and the ratio of Ad5 to Cy5-D **9b** is 1000k. (**B**) The pMOI is 2000 and the ratio of Ad5 to Cy5-D **9b** is 1000k. The difference between (**A**) and (**B**) is the Ad5 batch. Ad5 used for (**A**) was purchased from Hanbio, while (**B**) from Cyagen Biosciences. All Ad5 used for main text is the same with (**A**).

4.3.4 Copper(I)-catalyzed azide-alkyne cycloaddition (CuAAC) on dendron coated Ad5

4.3.4.1 Investigating the importance of a ligand in CuAAC

Stock solutions of propargyl-dendron **8** (1 mM in water, 10% DMSO), 7-hydroxy-3-azido-coumarin (6 mM in DMSO), $CuSO_4$ (1 mM in water), sodium ascorbate (NaAsc, 2 mM in water) and TBTA (1 mM in DMSO) were prepared. 10 µL of each propargyl-dendron **8**, 7-hydroxy-3-azido-coumarin and TBTA were added to 950 µL PBS. Then, $CuSO_4$ (10 µL) and NaAsc (10 µL) were added to obtain a 10 µM concentration of dendron. The reaction mixture was shaken for 1 h under protection from light. For the CuAAC without TBTA, 10 µL of PBS was added instead of the ligand. 6 µM coumarin in PBS was used as a control. The fluorescence spectra of coumarin (λ_{exc} = 375 nm; λ_{em} = 420-600 nm) was measured on a SPARK 20M microplate reader from TECAN Group Ltd.

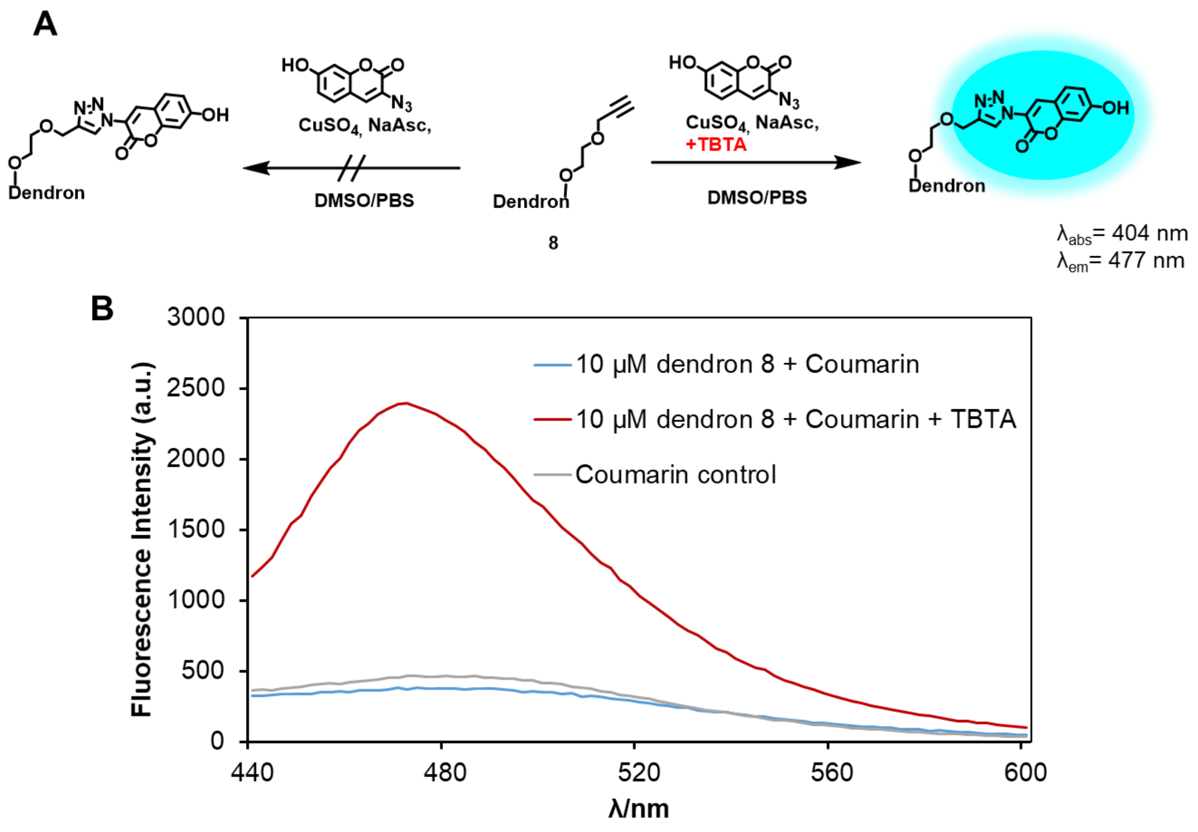

Figure S30. Investigation of ligand dependency of CuAAC. (**A**) Reaction scheme of CuAAC with and without TBTA (**B**) Fluorescence spectra of 10 µM dendron **8** incubated with 7-hydroxy-3-azido-coumarin and click reagents with and without addition of TBTA as well as 7-hydroxy-3-azido-coumarin as a control after incubation for 1 h. Only when adding TBTA, the fluorescence intensity at 477 nm was increased significantly indicating a successful CuAAC between dendron **8** and coumarin.

4.3.4.2 CuAAC on Ad5/dendron complexes

Stock solutions of dendrons **8** and **9a** (1 mM in DMSO), 7-hydroxy-3-azido-coumarin (6 mM in DMSO), $CuSO_4$ (1 mM in water), sodium ascorbate (NaAsc, 2 mM in water) and BTTAA (1mM in DMSO) were prepared. For CuAAC without Ad5, propargyl-dendron **8**, coumarin-azide and BTTAA were first added into PBS (final volume is 55 μL), then $CuSO_4$ and NaAsc were added, and shaked for 1 h (protect from light). 2.2 μL of every reagent were used that results in a concentration of 240 μM coumarin (the concentration of dendron is 40 μM). As negative control, coumarin alone was used as well as biotin-dendron **9a** that was treated under same CuAAC conditions.

For CuAAC on the Ad5 surface, Ad5 and propargyl-dendron **8** with an Ad5 concentration of 1.3×10^{11} vp/mL and an Ad5 to propargyl-dendron **8** ratio of 1:500k were first incubated for 1 h (the volume of Ad5 is 20.3 μL and the volume of propargyl-dendron **8** is 2.2 μL). Then, unbound dendron was removed by ultrafiltration (100 kDa) for 3 times (25 °C, 6000 rpm, 10 min). Subsequently, Ad5 or Ad5/dendron was incubated with CuAAC reagents for 1 h. As negative controls, biotin-dendron **9a** was treated under same conditions and propargyl-D **8** (without Ad5) was ultrafiltered for 3 times before incubated with other reagents in order to prove that free dendrons can be removed by ultrafiltration. The fluorescence intensity of coumarin was measured on a Varioskan LUX microplate reader (Thermo Fisher Scientific, USA).

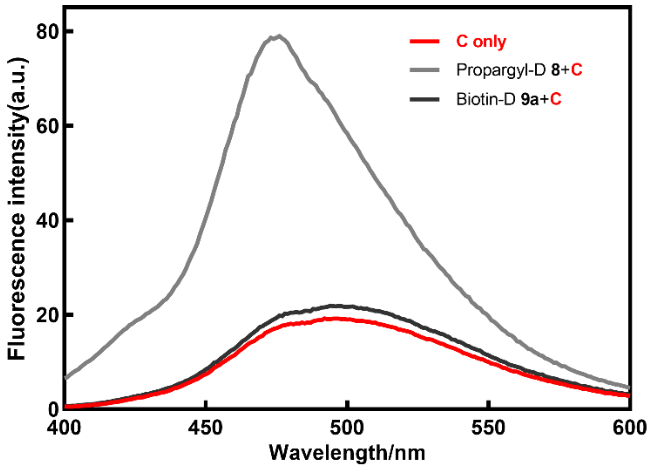

Figure S31. CuAAC with BTTAA. Propargyl-dendron **8** and biotin-dendron **9a** (negative control) were incubated with CuAAC reagents and BTTAA. 7-Hydroxy-3-azido-coumarin (C only) was used as control. Only for propargyl-dendron **8** treated with coumarin and CuAAC reagents a significant increase in fluorescence intensity at 477 nm was observed.

4.3.5 Kinetic binding analysis[14]

The following chapter is reproduced with permission from *ACS Nano* **2019**, *13*, 8749-8759, https://pubs.acs.org/doi/10.1021/acsnano.9b01484. Further permissions related to the material within this chapter excerpted should be directed to the ACS journal.

The interaction between biotin-dendron **9a** and Ad5 was studied by Bio-Layer Interferometry assays (BLI) from Octet96 (Pall ForteBio, CA, USA). In order to receive a significant signal for this binding event, we have immobilized biotin-dendron **9a** at the sensor surface and applied Ad5 as binding molecule. To immobilize the dendron at the surface of streptavidin-coated biosensors, we used biotin-dendron **9a**. The basic experiment contains four steps: Step 1 included hydration of the biosensor to record the baseline. Step 2: Immobilization of biotin-dendron **9a** on the streptavidin (SA) biosensor. Step 3: Washing and establishing the baseline. Step 4: Association of the Ad5. Step 5: dissociation (Fig. S32).

A significant interaction signal could be seen even in the presence of only 2 pM Ad5. The K_D (equilibrium dissociation constant) determined by this method is 1.27×10^{-12} M. We believe that this very strong binding could be a result of multivalent interactions between the large virus particles providing large numbers of binding sites and the sensor surface densely coated with dendrons. These results clearly support that there is a strong binding between biotin-dendron **9a** and Ad5 viruses.

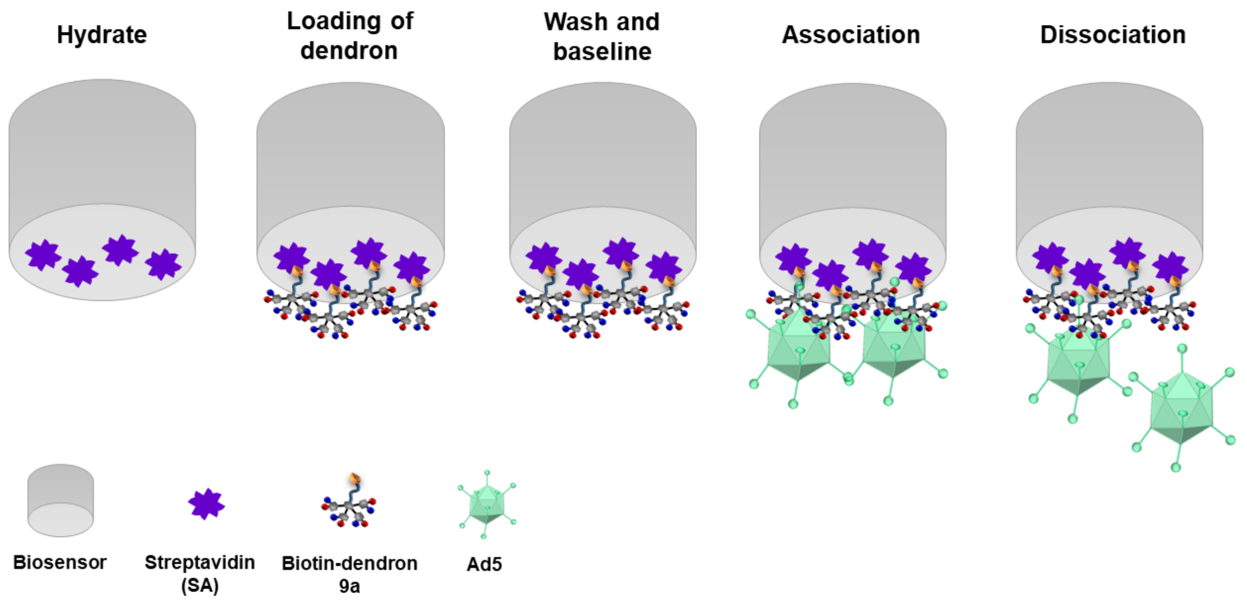

Figure S32. Workflow for dendron loading and dendron-Ad5 interaction assay[14] (adapted with permission from *ACS Nano* **2019**, *13*, 8749-8759, https://pubs.acs.org/doi/10.1021/acsnano.9b01484).

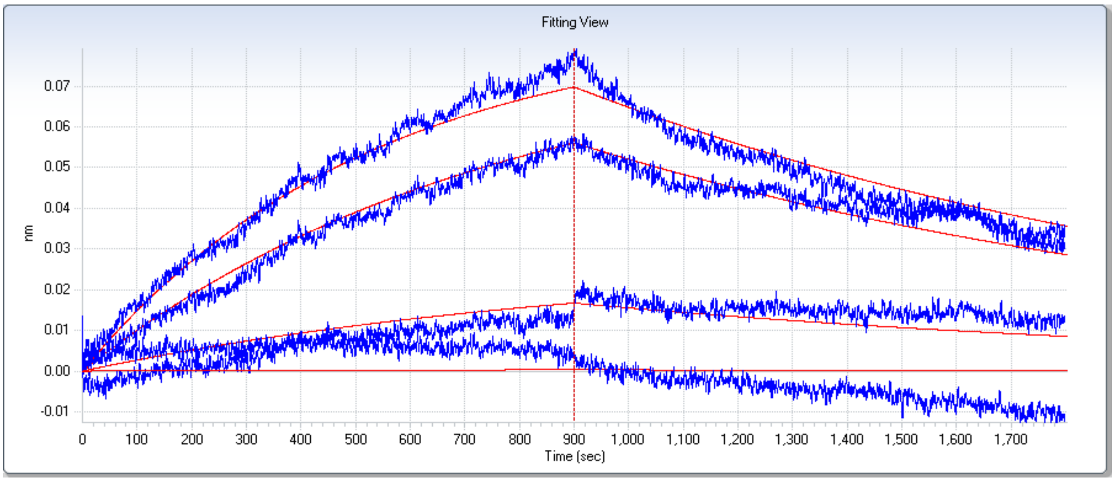

Figure S33: BLI analysis of Ad5 binding to biotin-dendron **9a** immobilized on streptavidin-coated biosensors. Association and dissociation curves are shown at different concentrations. Red lines represent regression modelling[14] (reprinted with permission from *ACS Nano* **2019**, *13*, 8749-8759, https://pubs.acs.org/doi/10.1021/acsnano.9b01484).

Table S6: Kinetic analysis results.[14] (reproduced with permission from *ACS Nano* **2019**, *13*, 8749-8759, https://pubs.acs.org/doi/10.1021/acsnano.9b01484).

Conc. (pM)	Response	K_D (M)	k_{on} (1/Ms)	k_{diss} (1/s)	k_{obs} (1/s)	Full R^2
**2**	0.076	$1.27{\cdot}10^{-12}$	$5.87{\cdot}10^{8}$	$7.47{\cdot}10^{-4}$	$1.92{\cdot}10^{-3}$	0.967526
**1**	0.0558	$1.27{\cdot}10^{-12}$	$5.87{\cdot}10^{8}$	$7.47{\cdot}10^{-4}$	$1.33{\cdot}10^{-3}$	0.967526
**0.5**	0.0129	$1.27{\cdot}10^{-12}$	$5.87{\cdot}10^{8}$	$7.47{\cdot}10^{-4}$	$1.04{\cdot}10^{-3}$	0.967526
**0.25**	0.0048	$1.27{\cdot}10^{-12}$	$5.87{\cdot}10^{8}$	$7.47{\cdot}10^{-4}$	$8.94{\cdot}10^{-4}$	0.967526

Conc.(nM): The molar concentration of the sample used in the association step. Response: Response calculated from the time window entered in the Steady State Analysis section. K_D (M): Equilibrium dissociation constant. k_{on} (1/Ms): Rate of association. k_{dis} (1/s): Rate of dissociation. k_{obs} (1/s): Observed binding rate. Full R^2: R^2 is the coefficient of determination which is an estimate of the goodness of the curve fit.

References

[1] F. Morgenroth, *Chem. Comm.* **1998**, 1139-1140.
[2] R. Stangenberg, I. Saeed, S. L. Kuan, M. Baumgarten, T. Weil, M. Klapper, K. Müllen, *Macromol. Rapid Commun.* **2014**, *35*, 152-160.
[3] M. L. Keshtov, E. I. Mal'tsev, D. V. Marochkin, A. V. Muranov, A. R. Khokhlov, *J. Polym. Sci. Part B Polym. Phys.* **2012**, *54*, 289-296.
[4] U. M. Wiesler, A. J. Berresheim, F. Morgenroth, G. Lieser, K. Müllen, *Macromolecules* **2001**, *34*, 187-199.
[5] R. Stangenberg, Y. Wu, J. Hedrich, D. Kurzbach, D. Wehner, G. Weidinger, S. L. Kuan, M. I. Jansen, F. Jelezko, H. J. Luhmann, D. Hinderberger, T. Weil, K. Müllen, *Adv. Healthcare Mater.* **2015**, *4*, 377-384.
[6] a) D. Baumann, D. Hofmann, S. Nullmeier, P. Panther, C. Dietze, A. Musyanovych, S. Ritz, K. Landfester, V. Mailänder, *Nanomedicine* **2012**, *8*, 699-713; b) S. Lerch, M. Dass, A. Musyanovych, K. Landfester, V. Mailänder, *Eur. J. Pharm. Biopharm.* **2013**, *84*, 265-274.
[7] J. Simon, T. Wolf, K. Klein, K. Landfester, F. R. Wurm, V. Mailänder, *Angew. Chem., Int. Ed.* **2018**, *57*, 5548-5553.
[8] D. Hofmann, S. Tenzer, M. B. Bannwarth, C. Messerschmidt, S.-F. Glaser, H. Schild, K. Landfester, V. Mailänder, *ACS Nano* **2014**, *8*, 10077-10088.
[9] S. Tenzer, *ACS Nano* **2011**, *5*, 7155-7167.
[10] R. A. Bradshaw, A. L. Burlingame, S. Carr, R. Aebersold, *Mol. Cell. Proteomics* **2006**, *5*, 787.
[11] a) S. Schöttler, G. Becker, S. Winzen, T. Steinbach, K. Mohr, K. Landfester, V. Mailänder, F. R. Wurm, *Nat. Nanotechnol.* **2016**, *11*, 372-377; b) M. Kokkinopoulou, J. Simon, K. Landfester, V. Mailander, I. Lieberwirth, *Nanoscale* **2017**, *9*, 8858-8870.
[12] J. C. Silva, M. V. Gorenstein, G. Z. Li, J. P. C. Vissers, S. J. Geromanos, *Mol. Cell. Proteomics* **2006**, *5*, 144-156.
[13] M. Okuno, M. Mezger, R. Stangenberg, M. Baumgarten, K. Müllen, M. Bonn, E. H. Backus, *Langmuir* **2015**, *31*, 1980-1987.
[14] Y. Wu, L. Li, L. Frank, J. Wagner, P. Andreozzi, B. Hammer, M. D'Alicarnasso, M. Pelliccia, W. Liu, S. Chakrabortty, S. Krol, J. Simon, K. Landfester, S. L. Kuan, F. Stellacci, K. Müllen, F. Kreppel, T. Weil, *ACS Nano* **2019**, *13*, 8749-8759.

Protein structure images used within this article were taken from rcsb.org:

PDB ID: 4NHH
Y. Wu, Anthony P. West, Helen J. Kim, Matthew E. Thornton, Andrew B. Ward, Pamela J. Bjorkman, Cell Reports 2013, 5, 1443-1455.

PDB ID: 1FZC
S. J. Everse, G. Spraggon, L. Veerapandian, M. Riley, R. F. Doolittle, Biochemistry 1998, 37, 8637-8642.

PDB ID: 5Z0B
J. Park, M. S. Kim, D. H. Shin (2018), Crystal structure of plasma-derived human serum albumin, doi: 10.2210/pdb5Z0B/pdb

Appendix: All identified Corona proteins

				Average (%), technical duplicates				Std, technical duplicates		
Accession	Peptide count	Unique peptides	Description	Liposome	Liposome-Dendron	Liposome-Dendrimer		Liposome	Liposome-Dendron	Liposome-Dendrimer
P04217	5	5	Alpha-1B-glycoprotein OS=Homo sapiens GN=A1BG PE=1 SV=4	0.10	0.11	0.27		0.07	0.01	0.02
P08697	7	7	Alpha-2-antiplasmin OS=Homo sapiens GN=SERPINF2 PE=1 SV=3	0.38	0.24	0.29		0.44	0.05	0.04
P02765	7	7	Alpha-2-HS-glycoprotein OS=Homo sapiens GN=AHSG PE=1 SV=1	0.66	0.72	0.28		0.07	0.14	0.00
P01019	3	3	Angiotensinogen OS=Homo sapiens GN=AGT PE=1 SV=1	0.00	0.14	0.13		0.00	0.01	0.01
P01008	37	37	Antithrombin-III OS=Homo sapiens GN=SERPINC1 PE=1 SV=1	0.69	3.82	1.76		0.17	0.25	0.39
P02647	32	30	Apolipoprotein A-I OS=Homo sapiens GN=APOA1 PE=1 SV=1	0.41	4.05	6.24		0.02	0.84	2.19
P02652	2	2	Apolipoprotein A-II OS=Homo sapiens GN=APOA2 PE=1 SV=1	0.00	0.17	0.22		0.00	0.06	0.01
P06727	33	31	Apolipoprotein A-IV OS=Homo sapiens GN=APOA4 PE=1 SV=3	0.54	2.55	3.14		0.02	0.41	0.10
P04114	66	64	Apolipoprotein B-100 OS=Homo sapiens GN=APOB PE=1 SV=2	2.21	0.79	1.01		0.27	0.14	0.14
P02649	19	19	Apolipoprotein E OS=Homo sapiens GN=APOE PE=1 SV=1	2.32	1.90	1.18		0.15	0.31	0.03
O14791	2	2	Apolipoprotein L1 OS=Homo sapiens GN=APOL1 PE=1 SV=5	0.08	0.06	0.10		0.05	0.01	0.00
P08519	4	3	Apolipoprotein(a) OS=Homo sapiens GN=LPA PE=1 SV=1	3.31	0.03	0.01		2.59	0.01	0.01
P02749	18	18	Beta-2-glycoprotein 1 OS=Homo sapiens GN=APOH PE=1 SV=3	0.52	7.83	4.95		0.18	0.54	0.34
P04003	16	14	C4b-binding protein alpha chain OS=Homo sapiens GN=C4BPA PE=1 SV=2	14.53	0.43	0.46		0.72	0.10	0.01
P10909	20	20	Clusterin OS=Homo sapiens GN=CLU PE=1 SV=1	6.14	5.71	4.28		2.06	0.59	0.19
P00740	7	6	Coagulation factor IX OS=Homo sapiens GN=F9 PE=1 SV=2	0.47	0.20	0.31		0.05	0.14	0.27
P00742	3	3	Coagulation factor X OS=Homo sapiens GN=F10 PE=1 SV=2	0.00	0.11	0.18		0.00	0.01	0.01
P00748	12	11	Coagulation factor XII OS=Homo sapiens GN=F12 PE=1 SV=3	2.02	0.52	0.67		0.00	0.07	0.02
P02745	3	3	Complement C1q subcomponent subunit A OS=Homo sapiens GN=C1QA PE=1 SV=2	0.22	0.50	0.47		0.02	0.10	0.01
P02746	8	8	Complement C1q subcomponent subunit B OS=Homo sapiens GN=C1QB PE=1 SV=3	0.01	0.81	1.58		0.00	0.08	0.10
P02747	5	4	Complement C1q subcomponent subunit C OS=Homo sapiens GN=C1QC PE=1 SV=3	0.02	1.03	1.62		0.01	0.20	0.38
P00736	21	19	Complement C1r subcomponent OS=Homo sapiens GN=C1R PE=1 SV=2	0.01	0.29	0.96		0.00	0.02	0.05
P09871	13	12	Complement C1s subcomponent OS=Homo sapiens GN=C1S PE=1 SV=1	0.01	0.80	1.10		0.00	0.34	0.18
P01024;O95568	109	106	Complement C3 OS=Homo sapiens GN=C3 PE=1 SV=2	5.93	1.99	3.22		2.66	0.15	0.31
P0C0L4	78	2	Complement C4-A OS=Homo sapiens GN=C4A PE=1 SV=2	0.14	0.73	1.56		0.03	0.06	0.10
P0C0L5	81	5	Complement C4-B OS=Homo sapiens GN=C4B PE=1 SV=2	0.43	2.21	4.71		0.08	0.19	0.31
P01031	26	26	Complement C5 OS=Homo sapiens GN=C5 PE=1 SV=4	0.03	0.14	0.49		0.00	0.01	0.05
P13671	26	26	Complement component C6 OS=Homo sapiens GN=C6 PE=1 SV=3	0.48	0.48	0.77		0.07	0.02	0.03
P10643	14	14	Complement component C7 OS=Homo sapiens GN=C7 PE=1 SV=2	0.11	0.49	0.80		0.08	0.06	0.04
P07357	2	2	Complement component C8 alpha chain OS=Homo sapiens GN=C8A PE=1 SV=2	0.02	0.03	0.09		0.02	0.01	0.01
P07358	6	6	Complement component C8 beta chain OS=Homo sapiens GN=C8B PE=1 SV=3	0.10	0.17	0.31		0.01	0.01	0.02
P02748	18	16	Complement component C9 OS=Homo sapiens GN=C9 PE=1 SV=2	0.24	0.55	0.69		0.03	0.05	0.11
P00751	12	11	Complement factor B OS=Homo sapiens GN=CFB PE=1 SV=2	0.46	0.49	0.44		0.13	0.02	0.13
P08603	68	58	Complement factor H OS=Homo sapiens GN=CFH PE=1 SV=4	0.09	2.32	2.79		0.02	0.26	0.21
Q03591	13	3	Complement factor H-related protein 1 OS=Homo sapiens GN=CFHR1 PE=1 SV=2	0.27	0.89	0.56		0.12	0.21	0.04
P36980	6	2	Complement factor H-related protein 2 OS=Homo sapiens GN=CFHR2 PE=1 SV=1	0.09	0.24	0.19		0.04	0.05	0.01
Q02985	5	2	Complement factor H-related protein 3 OS=Homo sapiens GN=CFHR3 PE=1 SV=2	0.00	0.05	0.05		0.00	0.01	0.01
Q9BXR6	3	2	Complement factor H-related protein 5 OS=Homo sapiens GN=CFHR5 PE=1 SV=1	0.04	0.04	0.01		0.05	0.02	0.01
P27105	4	3	Erythrocyte band 7 integral membrane protein OS=Homo sapiens GN=STOM PE=1 SV=3	0.76	0.01	0.00		0.04	0.00	0.00
P06396	38	37	Gelsolin OS=Homo sapiens GN=GSN PE=1 SV=1	0.79	3.60	1.84		0.05	0.45	0.19
P69905	3	3	Hemoglobin subunit alpha OS=Homo sapiens GN=HBA1 PE=1 SV=2	1.82	0.06	0.05		0.05	0.00	0.01
P68871	5	4	Hemoglobin subunit beta OS=Homo sapiens GN=HBB PE=1 SV=2	5.33	0.08	0.04		0.99	0.00	0.01
P02790	7	7	Hemopexin OS=Homo sapiens GN=HPX PE=1 SV=2	0.49	0.42	0.22		0.44	0.02	0.02
P04196	22	21	Histidine-rich glycoprotein OS=Homo sapiens GN=HRG PE=1 SV=1	0.70	2.34	2.15		0.03	0.14	0.20
Q14520	8	8	Hyaluronan-binding protein 2 OS=Homo sapiens GN=HABP2 PE=1 SV=1	0.17	0.71	0.55		0.05	0.11	0.06
P01876;P01877	8	7	Ig alpha-1 chain C region OS=Homo sapiens GN=IGHA1 PE=1 SV=2	0.41	0.25	0.40		0.05	0.02	0.00
P01857	11	4	Ig gamma-1 chain C region OS=Homo sapiens GN=IGHG1 PE=1 SV=1	2.49	2.49	3.17		0.12	0.07	0.19
P01859	9	3	Ig gamma-2 chain C region OS=Homo sapiens GN=IGHG2 PE=1 SV=2	1.15	0.44	0.43		0.09	0.03	0.04
P01860	14	6	Ig gamma-3 chain C region OS=Homo sapiens GN=IGHG3 PE=1 SV=2	0.12	0.93	1.36		0.09	0.45	0.49
P01861	8	2	Ig gamma-4 chain C region OS=Homo sapiens GN=IGHG4 PE=1 SV=1	0.01	0.01	0.01		0.01	0.00	0.00
P01834	7	7	Ig kappa chain C region OS=Homo sapiens GN=IGKC PE=1 SV=1	6.33	2.00	2.35		1.00	0.20	0.14
8Q6;P0CF74;P0(	6	4	Ig lambda-2 chain C regions OS=Homo sapiens GN=IGLC2 PE=1 SV=1	0.70	0.61	0.64		0.01	0.04	0.13
P01871	10	3	Ig mu chain C region OS=Homo sapiens GN=IGHM PE=1 SV=3	0.46	0.11	0.21		0.57	0.02	0.00
P17936	2	2	Insulin-like growth factor-binding protein 3 OS=Homo sapiens GN=IGFBP3 PE=1 SV=2	0.00	0.05	0.07		0.00	0.02	0.02
P35858	11	11	Insulin-like growth factor-binding protein complex acid labile subunit OS=Homo sapiens GN=IGFALS PE=1 SV=1	0.21	0.16	0.50		0.24	0.04	0.06
P08514	4	3	Integrin alpha-IIb OS=Homo sapiens GN=ITGA2B PE=1 SV=3	0.64	0.01	0.01		0.36	0.00	0.00
P05106	2	2	Integrin beta-3 OS=Homo sapiens GN=ITGB3 PE=1 SV=2	0.00	0.00	0.00		0.00	0.00	0.00
P19827	9	9	Inter-alpha-trypsin inhibitor heavy chain H1 OS=Homo sapiens GN=ITIH1 PE=1 SV=3	0.28	0.25	0.38		0.04	0.02	0.04
P19823	21	20	Inter-alpha-trypsin inhibitor heavy chain H2 OS=Homo sapiens GN=ITIH2 PE=1 SV=2	0.01	0.47	0.65		0.00	0.02	0.09
Q14624	44	44	Inter-alpha-trypsin inhibitor heavy chain H4 OS=Homo sapiens GN=ITIH4 PE=1 SV=4	0.40	2.06	1.17		0.03	0.17	0.18
P29622	5	5	Kallistatin OS=Homo sapiens GN=SERPINA4 PE=1 SV=3	0.11	0.14	0.20		0.04	0.03	0.00
P01042	23	22	Kininogen-1 OS=Homo sapiens GN=KNG1 PE=1 SV=2	0.21	1.48	1.26		0.00	0.07	0.07
P18428	7	6	Lipopolysaccharide-binding protein OS=Homo sapiens GN=LBP PE=1 SV=3	0.10	0.46	0.24		0.15	0.02	0.04
P51884	4	4	Lumican OS=Homo sapiens GN=LUM PE=1 SV=2	0.20	0.37	0.47		0.04	0.05	0.03
Q96PD5	2	2	N-acetylmuramoyl-L-alanine amidase OS=Homo sapiens GN=PGLYRP2 PE=1 SV=1	0.00	0.01	0.05		0.00	0.01	0.01
P36955	14	13	Pigment epithelium-derived factor OS=Homo sapiens GN=SERPINF1 PE=1 SV=4	1.56	0.46	0.44		0.54	0.03	0.02
P03952	19	18	Plasma kallikrein OS=Homo sapiens GN=KLKB1 PE=1 SV=1	0.64	0.45	0.56		0.03	0.05	0.01
P05155	14	13	Plasma protease C1 inhibitor OS=Homo sapiens GN=SERPING1 PE=1 SV=2	0.91	0.55	0.80		0.33	0.05	0.02
P05154	7	6	Plasma serine protease inhibitor OS=Homo sapiens GN=SERPINA5 PE=1 SV=3	0.11	0.29	0.20		0.08	0.01	0.03
P00747	47	43	Plasminogen OS=Homo sapiens GN=PLG PE=1 SV=2	0.07	2.02	2.69		0.03	0.06	0.47
P02775	4	4	Platelet basic protein OS=Homo sapiens GN=PPBP PE=1 SV=3	0.31	1.37	0.59		0.06	0.05	0.07
P02776;P10720	4	4	Platelet factor 4 OS=Homo sapiens GN=PF4 PE=1 SV=2	0.23	0.64	0.53		0.01	0.09	0.07
P02760	8	8	Protein AMBP OS=Homo sapiens GN=AMBP PE=1 SV=1	0.09	0.37	0.48		0.00	0.18	0.09
Q9UK55	3	3	Protein Z-dependent protease inhibitor OS=Homo sapiens GN=SERPINA10 PE=1 SV=1	0.00	0.13	0.21		0.00	0.01	0.02
P00734	31	29	Prothrombin OS=Homo sapiens GN=F2 PE=1 SV=2	1.00	3.15	2.18		0.27	0.16	0.11
P49908	5	5	Selenoprotein P OS=Homo sapiens GN=SELENOP PE=1 SV=3	0.03	0.61	0.73		0.03	0.06	0.07
P02787	12	12	Serotransferrin OS=Homo sapiens GN=TF PE=1 SV=3	0.23	0.24	0.13		0.15	0.04	0.02
P02768	67	63	Serum albumin OS=Homo sapiens GN=ALB PE=1 SV=2	25.98	12.72	15.44		3.39	0.67	0.99
P02743	9	9	Serum amyloid P-component OS=Homo sapiens GN=APCS PE=1 SV=2	0.34	1.90	0.35		0.05	0.17	0.04
P27169	6	6	Serum paraoxonase/arylesterase 1 OS=Homo sapiens GN=PON1 PE=1 SV=3	0.06	0.11	0.19		0.04	0.02	0.02
P02766	4	4	Transthyretin OS=Homo sapiens GN=TTR PE=1 SV=1	0.02	0.25	0.19		0.01	0.03	0.00
P02774	32	31	Vitamin D-binding protein OS=Homo sapiens GN=GC PE=1 SV=1	0.38	2.57	2.02		0.14	0.36	0.21
P04004	19	17	Vitronectin OS=Homo sapiens GN=VTN PE=1 SV=1	1.11	9.04	5.94		0.70	0.82	0.47
				100.00	100.00	100.00				

Figure S34. Average amount in % and the standard deviation of all identified proteins adsorbed to lipo-dendron and lipo-dendrimer in blood serum

				Average (%), technical triplicates				Std,technical triplicates		
Accession	Peptide count	Unique peptides	Description	PS-NH2	PS-Dendron	PS-Dendrimer		PS-NH2	PS-Dendron	PS-Dendrimer
;P63267;P68032;	9	2	Actin, aortic smooth muscle OS=Homo sapiens GN=ACTA2 PE=1 SV=1	0.25	0.30	0.34		0.04	0.04	0.09
3261;P60709;Q9B	15	6	Actin, cytoplasmic 2 OS=Homo sapiens GN=ACTG1 PE=1 SV=1	0.47	0.28	0.39		0.15	0.07	0.10
P61204	1	1	ADP-ribosylation factor 3 OS=Homo sapiens GN=ARF3 PE=1 SV=2	0.00	0.00	0.00		0.00	0.00	0.01
P01009	4	3	Alpha-1-antitrypsin OS=Homo sapiens GN=SERPINA1 PE=1 SV=3	0.14	0.25	0.13		0.05	0.02	0.06
P02765	6	5	Alpha-2-HS-glycoprotein OS=Homo sapiens GN=AHSG PE=1 SV=1	0.04	0.32	0.16		0.03	0.04	0.07
P01008	21	20	Antithrombin-III OS=Homo sapiens GN=SERPINC1 PE=1 SV=1	1.46	0.89	1.50		0.75	0.27	0.24
P02647	34	33	Apolipoprotein A-I OS=Homo sapiens GN=APOA1 PE=1 SV=1	5.65	7.66	9.51		0.58	0.69	1.12
P02652	4	4	Apolipoprotein A-II OS=Homo sapiens GN=APOA2 PE=1 SV=1	0.05	0.18	0.07		0.04	0.09	0.05
P06727	33	30	Apolipoprotein A-IV OS=Homo sapiens GN=APOA4 PE=1 SV=3	3.36	1.83	2.21		0.42	0.39	0.47
P04114	83	78	Apolipoprotein B-100 OS=Homo sapiens GN=APOB PE=1 SV=2	0.24	0.31	0.49		0.01	0.15	0.21
P02656	2	2	Apolipoprotein C-III OS=Homo sapiens GN=APOC3 PE=1 SV=1	2.04	2.51	1.22		1.34	0.92	0.36
P02649	20	20	Apolipoprotein E OS=Homo sapiens GN=APOE PE=1 SV=1	0.92	0.95	0.73		0.26	0.26	0.19
P02749	18	17	Beta-2-glycoprotein 1 OS=Homo sapiens GN=APOH PE=1 SV=3	0.10	1.84	1.61		0.05	0.32	0.28
Q562R1	6	3	Beta-actin-like protein 2 OS=Homo sapiens GN=ACTBL2 PE=1 SV=2	0.03	0.04	0.05		0.02	0.01	0.01
P04003	13	10	C4b-binding protein alpha chain OS=Homo sapiens GN=C4BPA PE=1 SV=2	0.41	0.44	0.46		0.02	0.07	0.07
Q9NP86	1	1	Calcium-binding protein 5 OS=Homo sapiens GN=CABP5 PE=1 SV=1	0.02	0.06	0.11		0.02	0.02	0.02
P10909	21	21	Clusterin OS=Homo sapiens GN=CLU PE=1 SV=1	10.56	3.60	8.92		2.82	0.34	1.34
P00740	6	5	Coagulation factor IX OS=Homo sapiens GN=F9 PE=1 SV=2	0.07	0.08	0.13		0.05	0.03	0.01
P12259	14	13	Coagulation factor V OS=Homo sapiens GN=F5 PE=1 SV=4	0.11	0.18	0.27		0.02	0.03	0.06
P00748	2	1	Coagulation factor XII OS=Homo sapiens GN=F12 PE=1 SV=3	0.11	0.13	0.10		0.04	0.06	0.05
P02745	1	1	Complement C1q subcomponent subunit A OS=Homo sapiens GN=C1QA PE=1 SV=2	0.00	0.05	0.04		0.00	0.05	0.04
P02746	7	7	Complement C1q subcomponent subunit B OS=Homo sapiens GN=C1QB PE=1 SV=3	0.10	0.62	0.57		0.05	0.12	0.08
P02747	5	4	Complement C1q subcomponent subunit C OS=Homo sapiens GN=C1QC PE=1 SV=3	0.39	0.75	0.70		0.14	0.18	0.18
P00736	19	19	Complement C1r subcomponent OS=Homo sapiens GN=C1R PE=1 SV=2	1.45	1.13	0.89		0.20	0.20	0.16
P09871	12	10	Complement C1s subcomponent OS=Homo sapiens GN=C1S PE=1 SV=1	0.14	0.27	0.34		0.04	0.04	0.04
P01024	103	99	Complement C3 OS=Homo sapiens GN=C3 PE=1 SV=2	0.65	2.02	1.86		0.02	0.14	0.18
P0C0L4	57	2	Complement C4-A OS=Homo sapiens GN=C4A PE=1 SV=2	0.28	0.34	0.38		0.14	0.07	0.22
P0C0L5	56	1	Complement C4-B OS=Homo sapiens GN=C4B PE=1 SV=2	0.41	0.50	0.56		0.21	0.10	0.33
P01031	13	13	Complement C5 OS=Homo sapiens GN=C5 PE=1 SV=4	0.02	0.07	0.10		0.02	0.02	0.03
P13671	11	11	Complement component C6 OS=Homo sapiens GN=C6 PE=1 SV=3	0.02	0.17	0.21		0.00	0.00	0.01
P10643	9	9	Complement component C7 OS=Homo sapiens GN=C7 PE=1 SV=2	0.03	0.11	0.19		0.02	0.03	0.05
P02748	17	16	Complement component C9 OS=Homo sapiens GN=C9 PE=1 SV=2	0.25	0.41	0.65		0.08	0.06	0.04
P00751	9	9	Complement factor B OS=Homo sapiens GN=CFB PE=1 SV=2	0.26	0.28	0.15		0.18	0.13	0.07
P08603;Q02985	53	47	Complement factor H OS=Homo sapiens GN=CFH PE=1 SV=4	1.27	1.48	1.44		0.38	0.24	0.36
Q03591	14	3	Complement factor H-related protein 1 OS=Homo sapiens GN=CFHR1 PE=1 SV=2	0.20	0.80	0.80		0.02	0.08	0.07
P36980	6	1	Complement factor H-related protein 2 OS=Homo sapiens GN=CFHR2 PE=1 SV=1	0.02	0.11	0.10		0.01	0.02	0.02
Q9BXR6	5	4	Complement factor H-related protein 5 OS=Homo sapiens GN=CFHR5 PE=1 SV=1	0.15	0.10	0.11		0.05	0.04	0.06
P02671	4	4	Fibrinogen alpha chain OS=Homo sapiens GN=FGA PE=1 SV=2	0.02	0.11	0.08		0.00	0.05	0.05
Q13643	1	1	Four and a half LIM domains protein 3 OS=Homo sapiens GN=FHL3 PE=1 SV=4	0.07	0.00	0.00		0.12	0.00	0.00
P06396	30	29	Gelsolin OS=Homo sapiens GN=GSN PE=1 SV=1	0.37	0.85	0.67		0.16	0.14	0.16
Q96EK6	1	1	Glucosamine 6-phosphate N-acetyltransferase OS=Homo sapiens GN=GNPNAT1 PE=1 SV=1	0.01	0.02	0.02		0.02	0.01	0.01
P22352	4	4	Glutathione peroxidase 3 OS=Homo sapiens GN=GPX3 PE=1 SV=2	0.37	0.38	0.53		0.33	0.23	0.44
P00738	7	7	Haptoglobin OS=Homo sapiens GN=HP PE=1 SV=1	0.12	0.30	0.13		0.01	0.03	0.02
P69905	2	2	Hemoglobin subunit alpha OS=Homo sapiens GN=HBA1 PE=1 SV=2	0.09	0.11	0.14		0.02	0.01	0.04
P68871	8	5	Hemoglobin subunit beta OS=Homo sapiens GN=HBB PE=1 SV=2	0.66	0.58	0.45		0.01	0.02	0.05
P02042	6	1	Hemoglobin subunit delta OS=Homo sapiens GN=HBD PE=1 SV=2	0.02	0.02	0.02		0.01	0.00	0.01
P69892;P69891	2	1	Hemoglobin subunit gamma-2 OS=Homo sapiens GN=HBG2 PE=1 SV=2	0.02	0.02	0.04		0.01	0.01	0.03
Q6ZVN8	1	1	Hemojuvelin OS=Homo sapiens GN=HFE2 PE=1 SV=1	0.21	0.10	0.24		0.05	0.02	0.02
P02790	9	8	Hemopexin OS=Homo sapiens GN=HPX PE=1 SV=2	0.11	0.51	0.19		0.04	0.03	0.01
P04196	12	10	Histidine-rich glycoprotein OS=Homo sapiens GN=HRG PE=1 SV=1	0.05	0.89	0.48		0.02	0.23	0.11
Q14520	3	2	Hyaluronan-binding protein 2 OS=Homo sapiens GN=HABP2 PE=1 SV=1	0.10	0.10	0.08		0.04	0.00	0.01
P01876;P01877	12	12	Ig alpha-1 chain C region OS=Homo sapiens GN=IGHA1 PE=1 SV=2	0.37	0.55	0.30		0.08	0.10	0.11
P01857	10	4	Ig gamma-1 chain C region OS=Homo sapiens GN=IGHG1 PE=1 SV=1	1.42	3.28	1.81		0.11	0.24	0.10
P01859	11	6	Ig gamma-2 chain C region OS=Homo sapiens GN=IGHG2 PE=1 SV=2	1.82	1.93	1.56		0.22	0.29	0.34
P01860	13	6	Ig gamma-3 chain C region OS=Homo sapiens GN=IGHG3 PE=1 SV=2	0.15	0.62	0.45		0.09	0.16	0.08
P01861	7	1	Ig gamma-4 chain C region OS=Homo sapiens GN=IGHG4 PE=1 SV=1	0.03	0.12	0.04		0.00	0.07	0.01
P01834	6	5	Ig kappa chain C region OS=Homo sapiens GN=IGKC PE=1 SV=1	1.13	2.47	1.36		0.20	1.22	0.66
;B9A064;P0CF74	5	5	Ig lambda-3 chain C regions OS=Homo sapiens GN=IGLC3 PE=1 SV=1	0.54	2.26	1.35		0.35	0.84	0.07
P01871	17	5	Ig mu chain C region OS=Homo sapiens GN=IGHM PE=1 SV=3	0.91	0.80	0.90		0.14	0.05	0.10
P04220	12	1	Ig mu heavy chain disease protein OS=Homo sapiens PE=1 SV=1	0.04	0.04	0.04		0.01	0.00	0.00
0B4J1X5;A0A0C4D	3	3	Immunoglobulin heavy variable 3-74 OS=Homo sapiens GN=IGHV3-74 PE=3 SV=1	0.14	0.11	0.10		0.03	0.03	0.06
A0A075B6S2	2	2	Immunoglobulin kappa variable 2D-29 OS=Homo sapiens GN=IGKV2D-29 PE=3 SV=1	0.49	0.46	0.44		0.02	0.04	0.10
P08514	6	6	Integrin alpha-IIb OS=Homo sapiens GN=ITGA2B PE=1 SV=3	0.04	0.04	0.04		0.03	0.01	0.01
Q14624	42	41	Inter-alpha-trypsin inhibitor heavy chain H4 OS=Homo sapiens GN=ITIH4 PE=1 SV=4	1.20	2.34	2.94		0.23	0.41	0.73
P13645	9	8	Keratin, type I cytoskeletal 10 OS=Homo sapiens GN=KRT10 PE=1 SV=6	0.11	0.07	0.09		0.04	0.03	0.04
P35527	15	12	Keratin, type I cytoskeletal 9 OS=Homo sapiens GN=KRT9 PE=1 SV=3	0.66	0.20	0.16		0.25	0.08	0.06
P04264	16	14	Keratin, type II cytoskeletal 1 OS=Homo sapiens GN=KRT1 PE=1 SV=6	0.54	0.26	0.28		0.04	0.02	0.01
Q7Z794	2	1	Keratin, type II cytoskeletal 1b OS=Homo sapiens GN=KRT77 PE=2 SV=3	0.94	0.66	0.49		0.43	0.36	0.41
P35908	5	4	Keratin, type II cytoskeletal 2 epidermal OS=Homo sapiens GN=KRT2 PE=1 SV=2	0.13	0.07	0.08		0.03	0.02	0.02
P04259	4	3	Keratin, type II cytoskeletal 6B OS=Homo sapiens GN=KRT6B PE=1 SV=5	0.11	0.08	0.08		0.02	0.04	0.04
P01042	21	20	Kininogen-1 OS=Homo sapiens GN=KNG1 PE=1 SV=2	0.40	1.26	0.82		0.13	0.15	0.05
P18428	8	8	Lipopolysaccharide-binding protein OS=Homo sapiens GN=LBP PE=1 SV=3	0.07	0.44	0.76		0.05	0.05	0.03
Q96A32	1	0	Myosin regulatory light chain 2, skeletal muscle isoform OS=Homo sapiens GN=MYLPF PE=2 SV=1	0.00	0.00	0.00				
Q9NRC9	1	1	Otoraplin OS=Homo sapiens GN=OTOR PE=1 SV=1	0.00	0.01	0.02		0.00	0.02	0.04
Q8NEN9	1	1	PDZ domain-containing protein 8 OS=Homo sapiens GN=PDZD8 PE=1 SV=1	0.06	0.03	0.07		0.05	0.01	0.01
P42336	1	1	Phosphatidylinositol 4,5-bisphosphate 3-kinase catalytic subunit alpha isoform OS=Homo sapiens GN=PIK3CA PE=1 SV=2	0.02	0.02	0.02		0.01	0.00	0.01
P05155	7	7	Plasma protease C1 inhibitor OS=Homo sapiens GN=SERPING1 PE=1 SV=2	0.10	0.39	0.43		0.04	0.01	0.06
P00747	22	21	Plasminogen OS=Homo sapiens GN=PLG PE=1 SV=2	0.52	0.92	0.65		0.32	0.34	0.38
P02775	5	5	Platelet basic protein OS=Homo sapiens GN=PPBP PE=1 SV=3	0.41	1.68	0.56		0.15	0.21	0.13
P02776	5	3	Platelet factor 4 OS=Homo sapiens GN=PF4 PE=1 SV=2	0.21	2.34	1.27		0.10	0.23	0.17
P10720	3	1	Platelet factor 4 variant OS=Homo sapiens GN=PF4V1 PE=1 SV=1	0.10	1.08	0.60		0.05	0.23	0.15
Q6S8J3;A5A3E0	9	4	POTE ankyrin domain family member E OS=Homo sapiens GN=POTEE PE=2 SV=3	0.14	0.07	0.17		0.11	0.04	0.08
P27918	7	6	Properdin OS=Homo sapiens GN=CFP PE=1 SV=2	0.23	0.36	0.39		0.03	0.08	0.03
Q92954	6	6	Proteoglycan 4 OS=Homo sapiens GN=PRG4 PE=1 SV=2	0.01	0.11	0.14		0.01	0.03	0.05
P00734	17	16	Prothrombin OS=Homo sapiens GN=F2 PE=1 SV=2	0.67	0.41	0.73		0.08	0.06	0.09
A6NIZ1	1	1	Ras-related protein Rap-1b-like protein OS=Homo sapiens PE=2 SV=1	0.01	0.02	0.04		0.01	0.01	0.01
Q99969	2	2	Retinoic acid receptor responder protein 2 OS=Homo sapiens GN=RARRES2 PE=1 SV=1	0.01	0.03	0.04		0.01	0.00	0.02
Q9H2S5	2	2	RING finger protein 39 OS=Homo sapiens GN=RNF39 PE=2 SV=2	0.22	0.04	0.30		0.25	0.03	0.26
P02787	30	29	Serotransferrin OS=Homo sapiens GN=TF PE=1 SV=3	0.42	0.35	0.38		0.19	0.09	0.22
P02768	65	63	Serum albumin OS=Homo sapiens GN=ALB PE=1 SV=2	40.66	23.77	15.79		5.46	0.42	1.02
P0DJI8	6	3	Serum amyloid A-1 protein OS=Homo sapiens GN=SAA1 PE=1 SV=1	0.14	0.75	0.69		0.02	0.06	0.01
P0DJI9	4	1	Serum amyloid A-2 protein OS=Homo sapiens GN=SAA2 PE=1 SV=1	0.13	0.53	0.48		0.07	0.01	0.17
P02743	9	8	Serum amyloid P-component OS=Homo sapiens GN=APCS PE=1 SV=2	0.24	0.85	1.92		0.05	0.05	0.13
Q15527	2	2	Surfeit locus protein 2 OS=Homo sapiens GN=SURF2 PE=1 SV=3	0.77	0.18	0.24		0.24	0.11	0.09
P03986	1	0	T-cell receptor gamma-2 chain C region OS=Homo sapiens GN=TRGC2 PE=1 SV=1	0.00	0.00	0.00				
P07996	8	6	Thrombospondin-1 OS=Homo sapiens GN=THBS1 PE=1 SV=2	0.01	0.05	0.08		0.00	0.01	0.02
P02766	7	7	Transthyretin OS=Homo sapiens GN=TTR PE=1 SV=1	0.18	0.39	0.48		0.03	0.09	0.10
P62256	1	1	Ubiquitin-conjugating enzyme E2 H OS=Homo sapiens GN=UBE2H PE=1 SV=1	1.67	2.44	2.77		0.62	0.39	0.45
Q8WVN8	1	1	Ubiquitin-conjugating enzyme E2 Q2 OS=Homo sapiens GN=UBE2Q2 PE=1 SV=1	0.00	0.02	0.02		0.00	0.03	0.03
P02774	25	23	Vitamin D-binding protein OS=Homo sapiens GN=GC PE=1 SV=1	0.54	0.75	0.80		0.15	0.13	0.13
P04004	24	24	Vitronectin OS=Homo sapiens GN=VTN PE=1 SV=1	4.79	7.65	14.41		0.36	0.79	3.01
Q6ZN30	1	1	Zinc finger protein basonuclin-2 OS=Homo sapiens GN=BNC2 PE=1 SV=1	2.61	1.44	1.76		0.69	0.78	1.39
Q49AA0	1	1	Zinc finger protein ZFP69 OS=Homo sapiens GN=ZFP69 PE=2 SV=2	0.01	0.23	0.10		0.02	0.24	0.13
				100.00	100.00	100.00				

Figure S35. Average amount in % and the standard deviation of all identified proteins adsorbed to PS-dendron and PS-dendrimer in blood serum

SUPPORTING INFORMATION

				Average (%), technical duplicates				Std, technical duplicates		
Accession	Peptide count	Unique peptides	Description	Liposome	Liposome-Dendron	Liposome-Dendrimer		Liposome	Liposome-Dendron	Liposome-Dendrimer
P04217	6	5	Alpha-1B-glycoprotein OS=Homo sapiens GN=A1BG PE=1 SV=4	0.05	0.06	0.13		0.08	0.01	0.01
P02765	10	10	Alpha-2-HS-glycoprotein OS=Homo sapiens GN=AHSG PE=1 SV=1	0.19	0.87	0.24		0.23	0.21	0.01
P01008	8	8	Antithrombin-III OS=Homo sapiens GN=SERPINC1 PE=1 SV=1	0.06	0.55	0.39		0.09	0.08	0.01
P02647	27	27	Apolipoprotein A-I OS=Homo sapiens GN=APOA1 PE=1 SV=1	0.59	2.79	3.05		0.29	1.24	0.17
P06727	32	31	Apolipoprotein A-IV OS=Homo sapiens GN=APOA4 PE=1 SV=3	0.70	1.16	1.69		0.63	0.69	0.00
P02656	2	2	Apolipoprotein C-III OS=Homo sapiens GN=APOC3 PE=1 SV=1	0.35	0.85	0.15		0.47	0.15	0.00
P02649	20	20	Apolipoprotein E OS=Homo sapiens GN=APOE PE=1 SV=1	0.75	1.20	0.85		1.07	0.28	0.02
P02749	20	20	Beta-2-glycoprotein 1 OS=Homo sapiens GN=APOH PE=1 SV=3	0.34	10.46	3.83		0.48	1.49	0.26
P04003	11	9	C4b-binding protein alpha chain OS=Homo sapiens GN=C4BPA PE=1 SV=2	4.00	0.34	0.36		5.66	0.05	0.03
Q9HA72	1	1	Calcium homeostasis modulator protein 2 OS=Homo sapiens GN=CALHM2 PE=2 SV=1	11.38	0.35	0.21		16.10	0.31	0.02
P49747	3	3	Cartilage oligomeric matrix protein OS=Homo sapiens GN=COMP PE=1 SV=2	0.00	0.81	0.16		0.00	0.62	0.04
O43866	3	3	CD5 antigen-like OS=Homo sapiens GN=CD5L PE=1 SV=1	0.00	0.02	0.05		0.00	0.01	0.01
P10909	20	19	Clusterin OS=Homo sapiens GN=CLU PE=1 SV=1	1.19	4.13	3.35		1.68	0.26	0.02
P00740	5	5	Coagulation factor IX OS=Homo sapiens GN=F9 PE=1 SV=2	0.00	0.06	0.10		0.00	0.01	0.00
P00748	14	13	Coagulation factor XII OS=Homo sapiens GN=F12 PE=1 SV=3	0.21	0.51	0.59		0.29	0.05	0.01
P02745	5	5	Complement C1q subcomponent subunit A OS=Homo sapiens GN=C1QA PE=1 SV=2	0.74	0.99	1.09		1.05	0.06	0.00
P02746	8	8	Complement C1q subcomponent subunit B OS=Homo sapiens GN=C1QB PE=1 SV=3	0.43	0.48	1.22		0.60	0.06	0.08
P02747	5	5	Complement C1q subcomponent subunit C OS=Homo sapiens GN=C1QC PE=1 SV=3	0.04	0.48	1.02		0.05	0.13	0.02
P00736	18	17	Complement C1r subcomponent OS=Homo sapiens GN=C1R PE=1 SV=2	0.40	0.20	0.45		0.08	0.12	0.04
P09871	17	17	Complement C1s subcomponent OS=Homo sapiens GN=C1S PE=1 SV=1	0.00	0.82	0.44		0.00	0.32	0.01
P01024	99	97	Complement C3 OS=Homo sapiens GN=C3 PE=1 SV=2	1.21	1.05	2.00		1.58	0.13	0.08
P0C0L4	59	1	Complement C4-A OS=Homo sapiens GN=C4A PE=1 SV=2	11.97	0.33	0.51		16.90	0.02	0.02
P0C0L5	60	2	Complement C4-B OS=Homo sapiens GN=C4B PE=1 SV=2	6.94	0.19	0.30		9.80	0.01	0.01
P13671	27	26	Complement component C6 OS=Homo sapiens GN=C6 PE=1 SV=3	0.00	0.30	0.34		0.00	0.09	0.01
P10643	11	10	Complement component C7 OS=Homo sapiens GN=C7 PE=1 SV=2	0.29	0.12	0.18		0.41	0.02	0.01
P07358	14	12	Complement component C8 beta chain OS=Homo sapiens GN=C8B PE=1 SV=3	1.35	0.33	0.32		1.57	0.09	0.00
P07360	4	4	Complement component C8 gamma chain OS=Homo sapiens GN=C8G PE=1 SV=3	0.00	0.03	0.13		0.00	0.01	0.00
P02748	5	5	Complement component C9 OS=Homo sapiens GN=C9 PE=1 SV=2	0.00	0.11	0.14		0.00	0.02	0.00
P00751	15	14	Complement factor B OS=Homo sapiens GN=CFB PE=1 SV=2	3.93	0.26	0.27		5.45	0.17	0.05
P08603;Q02985	66	59	Complement factor H OS=Homo sapiens GN=CFH PE=1 SV=4	0.64	1.92	2.32		0.75	0.62	0.07
Q03591	11	1	Complement factor H-related protein 1 OS=Homo sapiens GN=CFHR1 PE=1 SV=2	0.24	0.53	0.32		0.08	0.08	0.02
P36980	7	2	Complement factor H-related protein 2 OS=Homo sapiens GN=CFHR2 PE=1 SV=1	0.01	0.21	0.19		0.02	0.04	0.02
Q9BXR6	6	4	Complement factor H-related protein 5 OS=Homo sapiens GN=CFHR5 PE=1 SV=1	0.01	0.04	0.02		0.02	0.02	0.00
Q9NQ30	2	1	Endothelial cell-specific molecule 1 OS=Homo sapiens GN=ESM1 PE=1 SV=2	0.00	0.02	0.03		0.00	0.01	0.00
P02671	54	53	Fibrinogen alpha chain OS=Homo sapiens GN=FGA PE=1 SV=2	0.52	7.43	8.74		0.15	1.40	0.38
P02675	54	53	Fibrinogen beta chain OS=Homo sapiens GN=FGB PE=1 SV=2	0.67	10.68	11.93		0.95	1.08	0.24
P02679	38	33	Fibrinogen gamma chain OS=Homo sapiens GN=FGG PE=1 SV=3	4.12	11.74	18.63		0.67	1.62	0.13
P06396	33	33	Gelsolin OS=Homo sapiens GN=GSN PE=1 SV=1	0.04	1.24	1.24		0.06	0.20	0.00
P02790	19	18	Hemopexin OS=Homo sapiens GN=HPX PE=1 SV=2	0.00	0.70	0.95		0.00	0.09	0.05
P05546	8	8	Heparin cofactor 2 OS=Homo sapiens GN=SERPIND1 PE=1 SV=3	1.65	0.28	0.25		1.43	0.09	0.01
P04196	20	20	Histidine-rich glycoprotein OS=Homo sapiens GN=HRG PE=1 SV=1	0.00	2.71	1.62		0.01	0.11	0.26
Q14520	8	7	Hyaluronan-binding protein 2 OS=Homo sapiens GN=HABP2 PE=1 SV=1	0.00	0.41	0.43		0.00	0.04	0.00
P01876;P01877	9	8	Ig alpha-1 chain C region OS=Homo sapiens GN=IGHA1 PE=1 SV=2	1.14	0.17	0.18		0.28	0.06	0.02
P01857	10	5	Ig gamma-1 chain C region OS=Homo sapiens GN=IGHG1 PE=1 SV=1	1.56	1.68	1.75		1.33	0.67	0.20
P01859	7	3	Ig gamma-2 chain C region OS=Homo sapiens GN=IGHG2 PE=1 SV=2	0.33	0.25	0.23		0.40	0.05	0.01
P01860	12	5	Ig gamma-3 chain C region OS=Homo sapiens GN=IGHG3 PE=1 SV=2	1.22	0.51	0.76		0.77	0.21	0.09
P01861	7	1	Ig gamma-4 chain C region OS=Homo sapiens GN=IGHG4 PE=1 SV=1	0.15	0.09	0.09		0.17	0.03	0.00
P01834	5	5	Ig kappa chain C region OS=Homo sapiens GN=IGKC PE=1 SV=1	2.72	1.91	1.60		3.49	0.05	0.15
;A0M8Q6;B9A064;P0CG04;	6	3	Ig lambda-2 chain C regions OS=Homo sapiens GN=IGLC2 PE=1 SV=1	0.61	0.67	0.69		0.62	0.06	0.01
P0CF74	4	1	Ig lambda-6 chain C region OS=Homo sapiens GN=IGLC6 PE=4 SV=1	0.29	0.19	0.15		0.05	0.08	0.01
P01871;P04220	9	9	Ig mu chain C region OS=Homo sapiens GN=IGHM PE=1 SV=3	0.66	0.79	0.95		0.67	0.26	0.01
P17936	4	4	Insulin-like growth factor-binding protein 3 OS=Homo sapiens GN=IGFBP3 PE=1 SV=2	0.03	0.09	0.13		0.04	0.02	0.00
P35858	9	9	Insulin-like growth factor-binding protein complex acid labile subunit OS=Homo sapiens GN=IGFALS PE=1 SV=1	0.09	0.19	0.17		0.06	0.05	0.01
P19823	21	20	Inter-alpha-trypsin inhibitor heavy chain H2 OS=Homo sapiens GN=ITIH2 PE=1 SV=2	0.00	0.18	0.36		0.00	0.02	0.02
Q14624	42	42	Inter-alpha-trypsin inhibitor heavy chain H4 OS=Homo sapiens GN=ITIH4 PE=1 SV=4	0.15	1.71	0.95		0.21	0.78	0.04
P13645	14	14	Keratin, type I cytoskeletal 10 OS=Homo sapiens GN=KRT10 PE=1 SV=6	8.96	0.11	0.05		10.63	0.04	0.00
P19012	2	2	Keratin, type I cytoskeletal 15 OS=Homo sapiens GN=KRT15 PE=1 SV=3	1.52	0.07	0.05		2.15	0.01	0.01
P08779	1	1	Keratin, type I cytoskeletal 16 OS=Homo sapiens GN=KRT16 PE=1 SV=4	0.00	0.02	0.00		0.00	0.01	0.00
Q04695	2	2	Keratin, type I cytoskeletal 17 OS=Homo sapiens GN=KRT17 PE=1 SV=2	0.00	0.06	0.04		0.00	0.02	0.00
P35527	13	12	Keratin, type I cytoskeletal 9 OS=Homo sapiens GN=KRT9 PE=1 SV=3	0.40	0.19	0.04		0.57	0.01	0.00
P04264;P35908	20	18	Keratin, type II cytoskeletal 1 OS=Homo sapiens GN=KRT1 PE=1 SV=6	1.39	0.95	0.83		1.97	0.58	0.00
P01042	25	24	Kininogen-1 OS=Homo sapiens GN=KNG1 PE=1 SV=2	0.16	1.57	1.28		0.23	0.17	0.07
P18428	7	7	Lipopolysaccharide-binding protein OS=Homo sapiens GN=LBP PE=1 SV=3	0.00	0.31	0.18		0.00	0.12	0.01
P36955	13	13	Pigment epithelium-derived factor OS=Homo sapiens GN=SERPINF1 PE=1 SV=4	0.27	0.39	0.29		0.38	0.22	0.01
P03952	25	23	Plasma kallikrein OS=Homo sapiens GN=KLKB1 PE=1 SV=1	0.09	0.50	0.38		0.12	0.09	0.00
P05155	10	10	Plasma protease C1 inhibitor OS=Homo sapiens GN=SERPING1 PE=1 SV=2	0.32	0.46	0.54		0.45	0.35	0.02
P05154	5	5	Plasma serine protease inhibitor OS=Homo sapiens GN=SERPINA5 PE=1 SV=3	0.00	0.15	0.11		0.00	0.01	0.00
P00747;Q02325;Q15195	56	54	Plasminogen OS=Homo sapiens GN=PLG PE=1 SV=2	3.69	1.88	2.29		5.11	0.11	0.11
P02760	5	5	Protein AMBP OS=Homo sapiens GN=AMBP PE=1 SV=1	0.37	0.11	0.22		0.52	0.02	0.02
Q9HD43	1	0	Receptor-type tyrosine-protein phosphatase H OS=Homo sapiens GN=PTPRH PE=1 SV=3	0.00	0.00	0.00		0.00	0.00	0.00
P49908	4	4	Selenoprotein P OS=Homo sapiens GN=SELENOP PE=1 SV=3	0.00	0.26	0.23		0.00	0.07	0.00
P02768	55	54	Serum albumin OS=Homo sapiens GN=ALB PE=1 SV=2	16.92	9.14	9.66		20.95	0.78	0.49
P27169	10	10	Serum paraoxonase/arylesterase 1 OS=Homo sapiens GN=PON1 PE=1 SV=3	1.48	0.25	0.39		2.10	0.04	0.06
Q9H1Z9	1	1	Tetraspanin-10 OS=Homo sapiens GN=TSPAN10 PE=2 SV=1	0.00	0.43	1.04		0.00	0.20	0.22
Q9H0R3	3	2	Transmembrane protein 222 OS=Homo sapiens GN=TMEM222 PE=1 SV=2	0.00	0.40	0.36		0.00	0.03	0.03
P02774	29	28	Vitamin D-binding protein OS=Homo sapiens GN=GC PE=1 SV=1	0.29	1.07	0.58		0.13	0.05	0.02
P04004	20	19	Vitronectin OS=Homo sapiens GN=VTN PE=1 SV=1	0.17	5.48	3.21		0.25	0.53	0.19
				100.00	100.00	100.00				

Figure S36. Average amount in % and the standard deviation of all identified proteins adsorbed to lipo-dendron and lipo-dendrimer in blood plasma

SUPPORTING INFORMATION

				Average (%), technical triplicates				Std,technical triplicates		
Accession	Peptide count	Unique peptides	Description	PS-NH2	PS-Dendron	PS-Dendrimer		PS-NH2	PS-Dendron	PS-Dendrimer
P16885	6	6	1-phosphatidylinositol 4,5-bisphosphate phosphodiesterase gamma-2 OS=Homo sapiens GN=PLCG2 PE=1 SV=4	0.23	1.67	0.64		0.03	0.23	0.45
P43652	2	2	Afamin OS=Homo sapiens GN=AFM PE=1 SV=1	0.03	0.05	0.02		0.00	0.01	0.01
P01009	6	6	Alpha-1-antitrypsin OS=Homo sapiens GN=SERPINA1 PE=1 SV=3	0.06	0.11	0.06		0.01	0.01	0.05
P02765	9	9	Alpha-2-HS-glycoprotein OS=Homo sapiens GN=AHSG PE=1 SV=1	0.18	0.91	0.28		0.01	0.02	0.03
P03950	2	2	Angiogenin OS=Homo sapiens GN=ANG PE=1 SV=1	0.01	0.07	0.09		0.00	0.01	0.04
P02647	35	30	Apolipoprotein A-I OS=Homo sapiens GN=APOA1 PE=1 SV=1	8.66	3.55	3.02		0.92	0.06	1.11
P02652	5	5	Apolipoprotein A-II OS=Homo sapiens GN=APOA2 PE=1 SV=1	0.19	0.10	0.05		0.03	0.01	0.00
P06727	35	35	Apolipoprotein A-IV OS=Homo sapiens GN=APOA4 PE=1 SV=3	2.41	0.89	1.22		0.05	0.04	0.47
P04114	135	129	Apolipoprotein B-100 OS=Homo sapiens GN=APOB PE=1 SV=2	0.42	1.62	1.35		0.03	0.09	0.06
P02656	2	2	Apolipoprotein C-III OS=Homo sapiens GN=APOC3 PE=1 SV=1	2.04	1.52	0.68		0.28	0.11	0.54
P02649	22	22	Apolipoprotein E OS=Homo sapiens GN=APOE PE=1 SV=1	1.07	0.31	0.25		0.15	0.04	0.14
P02730	12	12	Band 3 anion transport protein OS=Homo sapiens GN=SLC4A1 PE=1 SV=3	0.13	0.15	0.22		0.01	0.01	0.04
P02749	16	15	Beta-2-glycoprotein 1 OS=Homo sapiens GN=APOH PE=1 SV=3	0.11	1.71	1.93		0.01	0.03	0.20
P04003	12	9	C4b-binding protein alpha chain OS=Homo sapiens GN=C4BPA PE=1 SV=2	0.28	0.21	0.14		0.03	0.01	0.04
P15169	3	3	Carboxypeptidase N catalytic chain OS=Homo sapiens GN=CPN1 PE=1 SV=1	0.40	0.26	0.23		0.05	0.04	0.14
O43866	5	5	CD5 antigen-like OS=Homo sapiens GN=CD5L PE=1 SV=1	0.05	0.04	0.04		0.00	0.00	0.01
P10909	24	23	Clusterin OS=Homo sapiens GN=CLU PE=1 SV=1	10.01	2.51	3.43		0.25	0.07	0.49
P00740	11	10	Coagulation factor IX OS=Homo sapiens GN=F9 PE=1 SV=2	0.20	0.36	0.19		0.03	0.02	0.08
P03951	15	15	Coagulation factor XI OS=Homo sapiens GN=F11 PE=1 SV=1	0.02	0.22	0.23		0.00	0.00	0.01
P05160	4	4	Coagulation factor XIII B chain OS=Homo sapiens GN=F13B PE=1 SV=3	0.00	0.04	0.02		0.00	0.00	0.01
P00736	6	6	Complement C1r subcomponent OS=Homo sapiens GN=C1R PE=1 SV=2	0.01	0.04	0.06		0.00	0.00	0.02
P06681	5	4	Complement C2 OS=Homo sapiens GN=C2 PE=1 SV=2	0.02	0.09	0.16		0.00	0.01	0.04
P01024	84	82	Complement C3 OS=Homo sapiens GN=C3 PE=1 SV=2	0.69	0.69	1.21		0.03	0.04	0.24
P0C0L5	45	7	Complement C4-B OS=Homo sapiens GN=C4B PE=1 SV=2	0.30	0.27	0.37		0.03	0.03	0.07
P13671	14	13	Complement component C6 OS=Homo sapiens GN=C6 PE=1 SV=3	0.35	1.01	0.76		0.04	0.08	0.31
P02748	12	12	Complement component C9 OS=Homo sapiens GN=C9 PE=1 SV=2	0.10	0.15	0.15		0.00	0.00	0.01
P00751	5	5	Complement factor B OS=Homo sapiens GN=CFB PE=1 SV=2	0.01	0.06	0.03		0.00	0.01	0.00
P08603;Q02985	45	40	Complement factor H OS=Homo sapiens GN=CFH PE=1 SV=4	0.02	0.54	0.74		0.00	0.05	0.14
Q03591;P36980	13	7	Complement factor H-related protein 1 OS=Homo sapiens GN=CFHR1 PE=1 SV=2	0.16	1.09	1.10		0.01	0.09	0.27
Q7Z5Q1	3	3	Cytoplasmic polyadenylation element-binding protein 2 OS=Homo sapiens GN=CPEB2 PE=2 SV=3	0.00	0.05	0.03		0.00	0.01	0.01
Q5TAQ9	4	2	DDB1- and CUL4-associated factor 8 OS=Homo sapiens GN=DCAF8 PE=1 SV=1	0.10	0.14	0.08		0.01	0.05	0.03
Q3B726	3	3	DNA-directed RNA polymerase I subunit RPA43 OS=Homo sapiens GN=TWISTNB PE=1 SV=1	0.02	0.05	0.01		0.01	0.01	0.01
P02671	52	49	Fibrinogen alpha chain OS=Homo sapiens GN=FGA PE=1 SV=2	1.96	10.78	10.82		0.03	0.41	1.69
P02675	55	55	Fibrinogen beta chain OS=Homo sapiens GN=FGB PE=1 SV=2	2.31	12.63	12.03		0.05	0.21	0.73
P02679	34	30	Fibrinogen gamma chain OS=Homo sapiens GN=FGG PE=1 SV=3	3.10	13.94	15.11		0.13	0.54	2.12
P02751	16	15	Fibronectin OS=Homo sapiens GN=FN1 PE=1 SV=4	0.08	0.37	0.13		0.01	0.02	0.09
P06396	26	26	Gelsolin OS=Homo sapiens GN=GSN PE=1 SV=1	0.11	0.47	0.60		0.01	0.03	0.07
Q9H0R5	4	3	Guanylate-binding protein 3 OS=Homo sapiens GN=GBP3 PE=1 SV=3	0.09	0.05	0.03		0.03	0.01	0.02
P00738;P00739	3	3	Haptoglobin OS=Homo sapiens GN=HP PE=1 SV=1	0.06	0.09	0.07		0.00	0.00	0.03
Q96MM6	2	2	Heat shock 70 kDa protein 12B OS=Homo sapiens GN=HSPA12B PE=1 SV=2	0.06	0.27	0.07		0.04	0.21	0.08
P02790	11	11	Hemopexin OS=Homo sapiens GN=HPX PE=1 SV=2	0.09	0.40	0.40		0.02	0.01	0.08
Q04756	4	4	Hepatocyte growth factor activator OS=Homo sapiens GN=HGFAC PE=1 SV=1	0.01	0.07	0.10		0.00	0.01	0.01
P04196	14	14	Histidine-rich glycoprotein OS=Homo sapiens GN=HRG PE=1 SV=1	0.04	1.04	1.06		0.01	0.01	0.18
Q14520	10	10	Hyaluronan-binding protein 2 OS=Homo sapiens GN=HABP2 PE=1 SV=1	0.63	3.19	1.06		0.04	0.16	0.48
P01876	12	5	Ig alpha-1 chain C region OS=Homo sapiens GN=IGHA1 PE=1 SV=2	0.40	0.48	0.44		0.02	0.01	0.03
P01877	9	2	Ig alpha-2 chain C region OS=Homo sapiens GN=IGHA2 PE=1 SV=3	0.04	0.06	0.04		0.00	0.00	0.01
P01857	11	4	Ig gamma-1 chain C region OS=Homo sapiens GN=IGHG1 PE=1 SV=1	0.98	1.70	1.42		0.04	0.08	0.13
P01859	11	5	Ig gamma-2 chain C region OS=Homo sapiens GN=IGHG2 PE=1 SV=2	0.77	0.67	0.66		0.03	0.01	0.04
P01860	13	4	Ig gamma-3 chain C region OS=Homo sapiens GN=IGHG3 PE=1 SV=2	0.15	0.30	0.39		0.01	0.02	0.08
P01861	11	2	Ig gamma-4 chain C region OS=Homo sapiens GN=IGHG4 PE=1 SV=1	0.02	0.05	0.04		0.00	0.00	0.01
P01834	6	6	Ig kappa chain C region OS=Homo sapiens GN=IGKC PE=1 SV=1	1.44	2.29	1.83		0.06	0.15	0.12
[illegible]M8Q6;P0CF74;P0C[illegible]	7	2	Ig lambda-3 chain C regions OS=Homo sapiens GN=IGLC3 PE=1 SV=1	1.22	1.86	1.00		0.23	0.06	0.09
P01871	14	5	Ig mu chain C region OS=Homo sapiens GN=IGHM PE=1 SV=3	0.70	0.41	0.50		0.02	0.01	0.02
P04220	11	2	Ig mu heavy chain disease protein OS=Homo sapiens PE=1 SV=1	0.22	0.31	0.24		0.00	0.04	0.10
P17936	2	2	Insulin-like growth factor-binding protein 3 OS=Homo sapiens GN=IGFBP3 PE=1 SV=2	0.01	0.05	0.05		0.01	0.00	0.04
P19823	4	2	Inter-alpha-trypsin inhibitor heavy chain H2 OS=Homo sapiens GN=ITIH2 PE=1 SV=2	0.07	0.10	0.06		0.02	0.00	0.01
Q14624	41	40	Inter-alpha-trypsin inhibitor heavy chain H4 OS=Homo sapiens GN=ITIH4 PE=1 SV=4	0.40	0.67	1.21		0.07	0.02	0.24
P01042	22	22	Kininogen-1 OS=Homo sapiens GN=KNG1 PE=1 SV=2	0.11	1.04	0.70		0.01	0.03	0.05
P18428	10	10	Lipopolysaccharide-binding protein OS=Homo sapiens GN=LBP PE=1 SV=3	0.05	0.26	0.53		0.01	0.01	0.10
P51884	6	6	Lumican OS=Homo sapiens GN=LUM PE=1 SV=2	0.17	0.06	0.08		0.02	0.01	0.02
Q14934	2	2	Nuclear factor of activated T-cells, cytoplasmic 4 OS=Homo sapiens GN=NFATC4 PE=1 SV=2	0.09	0.03	0.02		0.00	0.01	0.01
Q9UG56	2	2	Phosphatidylserine decarboxylase proenzyme, mitochondrial OS=Homo sapiens GN=PISD PE=2 SV=4	0.15	0.35	0.25		0.02	0.01	0.05
P03952	13	12	Plasma kallikrein OS=Homo sapiens GN=KLKB1 PE=1 SV=1	0.02	0.12	0.10		0.00	0.00	0.06
P00747	26	26	Plasminogen OS=Homo sapiens GN=PLG PE=1 SV=2	0.02	0.34	0.25		0.00	0.01	0.07
Q6S8J3	5	2	POTE ankyrin domain family member E OS=Homo sapiens GN=POTEE PE=2 SV=3	0.08	0.04	0.02		0.01	0.00	0.01
Q6S5H5	3	2	POTE ankyrin domain family member G OS=Homo sapiens GN=POTEG PE=2 SV=5	0.04	0.07	0.03		0.07	0.06	0.03
P27918	5	5	Properdin OS=Homo sapiens GN=CFP PE=1 SV=2	0.06	0.12	0.07		0.01	0.01	0.03
P02760	3	3	Protein AMBP OS=Homo sapiens GN=AMBP PE=1 SV=1	0.01	0.03	0.02		0.00	0.00	0.00
Q92954	15	12	Proteoglycan 4 OS=Homo sapiens GN=PRG4 PE=1 SV=2	0.60	0.34	0.36		0.25	0.02	0.00
P00734	3	2	Prothrombin OS=Homo sapiens GN=F2 PE=1 SV=2	0.04	0.05	0.04		0.01	0.00	0.01
Q99969	3	3	Retinoic acid receptor responder protein 2 OS=Homo sapiens GN=RARRES2 PE=1 SV=1	0.01	0.08	0.08		0.00	0.01	0.02
Q13103	5	4	Secreted phosphoprotein 24 OS=Homo sapiens GN=SPP2 PE=1 SV=1	0.15	0.03	0.02		0.02	0.01	0.01
P49908	4	3	Selenoprotein P OS=Homo sapiens GN=SELENOP PE=1 SV=3	0.01	0.09	0.03		0.00	0.01	0.02
P02787	22	20	Serotransferrin OS=Homo sapiens GN=TF PE=1 SV=3	0.05	0.29	0.14		0.00	0.01	0.07
P02768;Q92985	65	58	Serum albumin OS=Homo sapiens GN=ALB PE=1 SV=2	46.77	14.04	11.69		1.29	0.56	0.91
P0DJI8	6	2	Serum amyloid A-1 protein OS=Homo sapiens GN=SAA1 PE=1 SV=1	0.02	0.05	0.05		0.00	0.00	0.02
P0DJI9	5	2	Serum amyloid A-2 protein OS=Homo sapiens GN=SAA2 PE=1 SV=1	0.04	0.13	0.09		0.00	0.02	0.02
P27169	11	11	Serum paraoxonase/arylesterase 1 OS=Homo sapiens GN=PON1 PE=1 SV=3	0.19	0.08	0.08		0.00	0.00	0.01
Q92563	4	4	Testican-2 OS=Homo sapiens GN=SPOCK2 PE=1 SV=1	0.23	0.59	0.66		0.03	0.04	0.12
P02766	8	8	Transthyretin OS=Homo sapiens GN=TTR PE=1 SV=1	0.18	0.38	0.59		0.01	0.02	0.00
P02774	30	29	Vitamin D-binding protein OS=Homo sapiens GN=GC PE=1 SV=1	0.61	1.01	0.56		0.04	0.08	0.04
P04070	4	3	Vitamin K-dependent protein C OS=Homo sapiens GN=PROC PE=1 SV=1	0.05	0.00	0.00		0.01	0.00	0.00
P04004	26	25	Vitronectin OS=Homo sapiens GN=VTN PE=1 SV=1	6.07	7.08	14.96		0.06	0.17	0.56
Q8N9V3	3	2	WD repeat, SAM and U-box domain-containing protein 1 OS=Homo sapiens GN=WDSUB1 PE=1 SV=3	0.10	0.04	0.02		0.01	0.02	0.01
Q6ZN30	3	3	Zinc finger protein basonuclin-2 OS=Homo sapiens GN=BNC2 PE=1 SV=1	0.82	0.57	0.39		0.42	0.05	0.20
				100.00	100.00	100.00	0.00	5.27	4.35	13.96

Figure S 37. Average amount in % and the standard deviation of all identified proteins adsorbed to PS-dendron and PS-dendrimer in blood plasma.
